# CRYSTAL23: A Program for Computational Solid State Physics and Chemistry

**DOI:** 10.1021/acs.jctc.2c00958

**Published:** 2022-12-11

**Authors:** Alessandro Erba, Jacques K. Desmarais, Silvia Casassa, Bartolomeo Civalleri, Lorenzo Donà, Ian J. Bush, Barry Searle, Lorenzo Maschio, Loredana Edith-Daga, Alessandro Cossard, Chiara Ribaldone, Eleonora Ascrizzi, Naiara L. Marana, Jean-Pierre Flament, Bernard Kirtman

**Affiliations:** † Dipartimento di Chimica, Università di Torino, via Giuria 5, 10125 Torino, Italy; ‡ STFC Rutherford Appleton Laboratory, Chilton Didcot, Oxfordshire OX11 0QX, United Kingdom; § SFTC Daresbury Laboratory, Daresbury, Cheshire WA4 4AD, United Kingdom; ∥ Université de Lille, CNRS, UMR 8523 — PhLAM — Physique des Lasers, Atomes et Molécules, 59000 Lille, France; ⊥ Department of Chemistry and Biochemistry, University of California, Santa Barbara, California 93106, United States

## Abstract

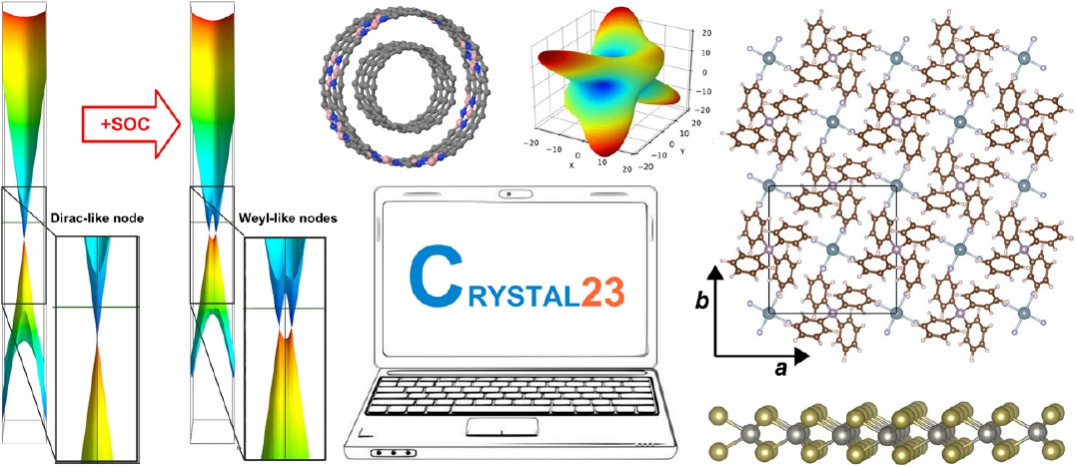

The Crystal program for quantum-mechanical simulations of materials has been bridging the realm of molecular quantum chemistry to the realm of solid state physics for many years, since its first public version released back in 1988. This peculiarity stems from the use of atom-centered basis functions within a linear combination of atomic orbitals (LCAO) approach and from the corresponding efficiency in the evaluation of the exact Fock exchange series. In particular, this has led to the implementation of a rich variety of hybrid density functional approximations since 1998. Nowadays, it is acknowledged by a broad community of solid state chemists and physicists that the inclusion of a fraction of Fock exchange in the exchange-correlation potential of the density functional theory is key to a better description of many properties of materials (electronic, magnetic, mechanical, spintronic, lattice-dynamical, etc.). Here, the main developments made to the program in the last five years (i.e., since the previous release, Crystal17) are presented and some of their most noteworthy applications reviewed.

## 1 Introduction

The past decade has witnessed a fast growth of the community of condensed-matter computational physicists and chemists. Such a large, diverse, vibrant community relies on robust and efficient simulation software programs that should be able to evolve in order to reflect (or ideally anticipate) its needs. Arguably, density functional theory (DFT) represents the method of choice in the calculation, interpretation, and prediction of properties of materials.^1−4^ Several DFT-based software packages are available, which provide implemented algorithms for a rich spectrum of possible applications in materials science, to name a few, Vasp, Quantum-Espresso, Abinit, Castep, Wien2k, NWChem, CP2K, Turbomole, PySCF, and others.^5−13^

In this context, the Crystal package has been bringing some diversity to the field of computational condensed matter science since its first public release back in 1988, thanks to several distinctive features. Among others, (i) the use of atom-centered local basis functions versus plane waves, (ii) a quantum-chemistry perspective with the first ever periodic implementation of the Hartree–Fock (HF) method, and (iii) an extensive exploitation of space symmetries as well as point symmetries at all steps of the calculation. The combination of these three factors allowed for the very efficient implementation of the infinite Fock exchange series, which then resulted to be key a few years later to an effective implementation of so-called “hybrid” exchange-correlation (xc) density functional approximations (DFAs). Back in 1998, Crystal provided the community with the first periodic implementation of global hybrid xc functionals and remains unchallenged in terms of their computational efficiencies. Nowadays, the effectiveness of hybrid xc functionals, with inclusion of a fraction of exact Fock exchange, over plain DFAs is widely acknowledged for the description of a variety of properties of materials (electronic structure, elasticity, linear and nonlinear optical response, lattice dynamics, etc.). In particular, Fock exchange proves crucial in the description of magnetism (collinear and noncollinear magnetization, spin–orbit coupling, spintronics, etc.).^14−25^

As developers, we aim at extending the application domain of DFT methodologies in a solid state context toward larger (i.e., more realistic) structural models and toward the description of more complex physical phenomena, with a higher accuracy. In this review paper, we illustrate the developments made to the program since its last major release, namely, Crystal17.^26^

## 2 Two-Component Density Functional Theory and Spin–Orbit Coupling

### 2.1 Two-Component Spinor Basis

Relativistic effects in quantum chemistry and materials physics refer to corrections to the Schrödinger equation from an account that the speed of light is finite and constant. Such corrections become increasingly important moving down the periodic table, to heavier elements, in which the effective velocities of the electrons becomes non-negligeable when compared to the speed of light. Relativistic effects can be classified into two categories, depending on whether or not they are described through scalar operators in the Hamiltonian. The first category comprises scalar-relativistic (SR) effects, and the second is here loosely referred to as spin–orbit coupling (SOC) effects. While SR effects have been treatable in Crystal since 1988,^27−29^ a treatment of SOC was still lacking.

The reformulation of the (one-component) Schrödinger equation that is consistent with the postulates of special relativity is the four-component Dirac equation. In the Dirac equation, a crystalline orbital (CO) is a 4 × 1 vector function (a four-component “spinor”), rather than a 1 × 1 scalar function ψ_**k**_^Schröd^(**r**) of space (a one-component, 1c, spinor), as well as the electron quasi-momentum **k** (i.e., the sampling point in the first-Brillouin zone, FBZ). More explicitly, a Schrödinger CO ket reads
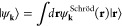
1while a Dirac ket is decomposed as
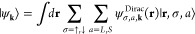
2in which σ is a spin index, and *a* denotes the so-called “large” and “small” components of the Dirac wave function, principally related to positive (electronic) and negative (positronic) energy solutions of the Dirac equation, respectively.

In quantum chemistry and materials physics, interest is dominated by the electronic solution of the Dirac equation. It is therefore common practice to write |ψ_**k**_⟩ in a basis of two-component (2c, or Pauli) spinors,
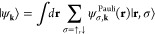
3where ψ_σ,**k**_^Pauli^(**r**) are components of the 2 × 1 CO vector **ψ**_**k**_^Pauli^(**r**),
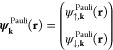
4Comparing eq 1 and eq 3, one immediate consequence is that the Kohn–Sham equation
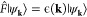
5in Pauli spinor representation leads to a 2 × 2 Fock operator, *F̂*, instead of a 1 × 1 Fock in the Schrödinger spinor basis. A relativistic theory including spin is then associated with a two-component self-consistent field (2c-SCF), rather than 1c-SCF, procedure. Such a 2c-SCF strategy has recently been implemented in the Crystal code.^30−32^

In matrix form, eq 5 in the Schrödinger spinor basis reads
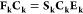
6and in the Pauli spinor basis
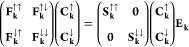
7where **E**_**k**_ is the diagonal matrix of band structure energies ϵ_*i*_(**k**), having size *N* × *N* for a calculation with *N* basis functions in the Schrödinger spinor basis, or size 2*N* × 2*N* in the Pauli spinor basis. **C**_**k**_^σ^ is the *N* × 2*N* matrix of expansion coefficients *C*_μ,*i*_^σ^(**k**) of the Pauli COs in Bloch functions,
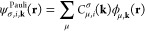
8while for Schrödinger spinors, we make use of the *N* × *N* matrix **C**_**k**_ with elements *C*_μ,*i*_(**k**),
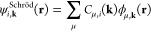
9Finally, in eq 7, **S**_**k**_^*σσ*′^ and **F**_**k**_^*σσ*′^ are the *N* × *N* spin blocks of the Bloch function overlap and Fock matrices.

For Crystal, in eqs 8 and 9, the Bloch functions are the inverse Fourier transform of *pure-real* atom-centered local functions (termed atomic orbitals, AOs),
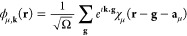
10with **a**_μ_ being the position of the atom on which χ_μ_ is centered in the reference cell **0**, 1 is the imaginary unit, and Ω is the volume of the FBZ. In eq 10, the sum over **g** is henceforth understood to extend over the full set of lattice vectors. More specifically, an AO χ_μ_ is here a linear combination of normalized real-solid-spherical harmonic Gaussian type functions (RSSH-GTF, see Section 4.1 for an exact definition).^33^

### 2.2 Spin–Orbit Coupling

#### 2.2.1 Relativistic Effective Potentials

In the present implementation, relativistic operators are represented as effective potentials (REP). In this approach, the many-electron problem is partitioned into one involving only the core electrons and one describing the core–valence and valence–valence interactions. The core electron problem has already been solved using a sufficiently accurate variant of the four-component Dirac equation (i.e., HF or post-HF Dirac-Coulomb, Dirac-Coulomb-Breit, or more accurate variants, possibly including further contributions to the electron–electron interaction from quantum electrodynamics). The solution of the core electron problem allows one to extract a relativistic effective core potential *Ŵ*. See refs (34−38) for exact details on the extraction procedure of *Ŵ*.

In practice, it is customary (and convenient) to express *Ŵ* as a sum of atom-centered monoelectronic operators *ŵ*,
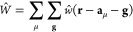
11For notational convenience, we drop the dependence of *ŵ* on the center μ, lattice vector **g**, and the electron coordinate **r** and simply write *ŵ* for one of the terms in eq 11. In a Pauli spinor basis *ŵ* is again a 2 × 2 matrix with elements *ŵ*^*σσ*′^.

As first suggested in ref (34), it is convenient for computational purposes to write *ŵ* as a sum of two terms. The first term *v̂* (representing SR effects) is a spin-averaged operator (the so-called averaged REP, AREP), while the second term *û* accounts for all spin-dependent relativistic effects (the so-called spin–orbit REP, SOREP),
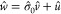
12where σ̂_0_ is a 2 × 2 unit matrix. In the end, both the AREP *v̂* and SOREP *û* are written using a sufficiently large sum of products of angular and radial operators. For the AREP,
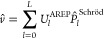
13awhile for the SOREP,
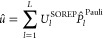
13bIn the present implementation, *L* has a maximum value of 4. The radial operators appearing in eqs 13a and 13b consist of a linear combination of solid Gaussian functions,
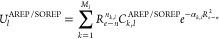
14with *R*_*e*–*n*_ being the electron–core distance. That is to say *R*_*e*–*n*_ is a shorthand notation for *R*_*e*–*n*_ = |**r** – **a**_μ_– **g**| for one of the terms in eq 11. *n*_*k*,*l*_ = 0,1,2,···, as well as *C*_*k*,*l*_^AREP/SOREP^ and α_*k*,*l*_ are parameters that are obtained from the solution of the core electron problem. In the case of the AREP *v̂*^σσ^, the operators *P̂*_*l*_^Schröd^, being angular projectors onto Schrödinger spinors, are *pure-real*. For the SOREP *û*^*σσ*′^, the angular projectors onto Pauli spinors *P̂*_*l*_^Pauli^, are instead *complex*. Matrix elements of the AREP/SOREP in an AO basis are written as, for instance, for the SOREP operator,
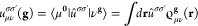
15where we have made use of the overlap distribution,
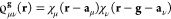
16Following from eq 16, it is expedient to also introduce the current overlap distribution **ζ**_*μν*_^**g**^(**r**),
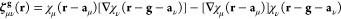
17The integrals involved in AREP matrix elements are calculated using an approach by McMurchie and Davidson.^39^ The procedure for SOREP integrals discussed in refs (40 and 41) is closely related to the same scheme of ref (39). The routines implemented in Crystal for the SOREP integrals are based on those of ref (41), and similar routines have also been implemented in the Epciso program of ref (42), in part by one of the present authors. These integrals are evaluated directly in a Cartesian GTF basis before a final transformation to the RSSH-GTF basis. This is in contrast to all other integrals in Crystal, which are instead evaluated directly in the RSSH-GTF basis, using a scheme first described by Saunders in ref (33), as is briefly reviewed in Section 4.1.

For extended periodic systems, as explained in refs (27−29), integrals of the form given in eq 15 are selected based on a screening criterion, employing the adjoined Gaussian χ̃_μ_ of shell μ (i.e., an *s*-type Gaussian with an exponent coinciding with the lowest one in shell μ, see the Crystal manual for a more ample discussion). The screening criterion for REPs makes use of the adjoined Gaussian overlap distribution,
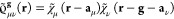
18and AREP, as well as SOREP integrals, are only evaluated if the overlap between  and the most diffuse Gaussian defining the AREP/SOREP in eq 14 is larger than a preset threshold 1 × 10^–TOLPSEUD^.

In relation to eq 14, different authors use variable definitions for the coefficients *C*_*k,l*_^SOREP^, which can differ by combinations of factors such as 2/*l*, 2/(2*l* + 1), as well as *R*_*e*–*n*_^2^. A series of keywords (INTERNAL, STUTTGART, COLUMBUS, and TOULOUSE) are provided to help the user define a SOREP from the Crystal input, using some of the definitions appearing in the literature.

#### 2.2.2 Internal Libraries of REPs

**Figure 1:**
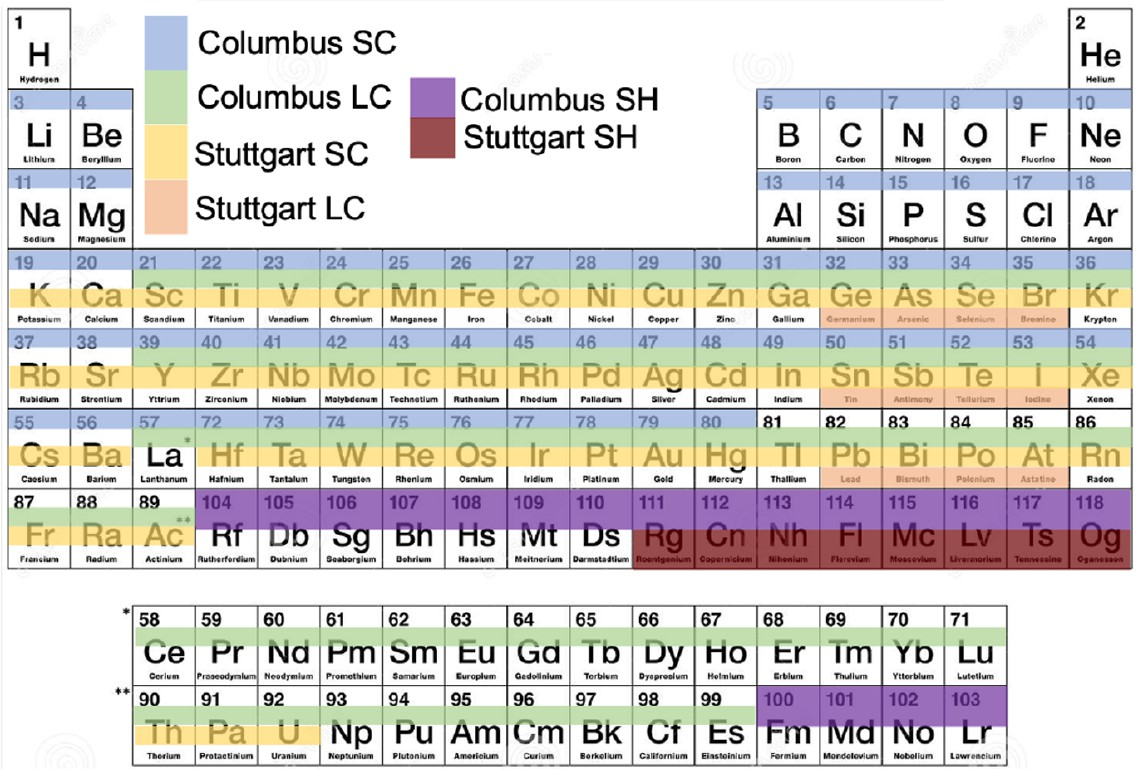
Availability of relativistic effective small core (SC) and large core (LC) potentials, as well as potentials for super heavy (SH) elements, including AREP and SOREP operators, for calculations with spin–orbit coupling and associated keywords from the Crystal input.

New internal libraries of AREP and SOREP operators are accessible from the Crystal input, using one of six keywords (STUTSC, STUTLC, STUTSH, COLUSC, COLULC, and COLUSH), as displayed in Figure 1. These nearly complete REP libraries have been implemented, based on the “shape-consistent” potentials of refs (43−49) and the “energy-consistent” potentials of refs (36 and 50−62). Accompanying molecular basis sets are available in clickable periodic tables at the cited web addresses.^63,64^ Corresponding basis sets for solids or low-dimensional periodic systems to go along with the REPs can be generated by decontracting the molecular sets and possibly also removing the most diffuse Gaussian functions. Some periodic basis sets for use with the STUTSC potentials are also available on the Crystal website.^65^

With regard to the STUTSC and STUTLC REPs, these are the ECPXXMDF ones (where XX is the number of core electrons effectively treated by the REP, and MDF indicates “multiconfigurational Dirac-Fock”, usually employing either the low-frequency or frequency-dependent Dirac–Coulomb–Breit Hamiltonians), for which most AREP operators are also available in Crystal format by following the link at ref (63). A few additional remarks follow:

- The STUTSH potentials correspond to the ECPXXMDFQ (not the ECPXXMDFB) ones, whose AREP parts can be found at the same web address.
- For the lanthanide series, “energy-consistent” potentials including spin–orbit operators are also available from ref (66), to go along with the small-core ECPXXMWB AREPs available in Crystal format at ref (63). These were, however, obtained from a very different approach to the potentials included in the present internal library.
- Finally, we note that the many-body core-polarization operators of the STUTLC potentials, which provide a correction for the frozen-core approximation, have not been implemented.

#### 2.3.3 Spin–Orbit Coupling Operator

Returning to eq 13b, the symmetries of the complex operators *P̂*_*l*_^Pauli^ permit one to derive the following relations for the SOREP matrix elements of eq 15 in an AO basis. For the pure imaginary diagonal spin-blocks,^67,68^
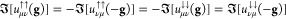
19where  denotes the imaginary part, and for the complex off-diagonal spin-blocks,
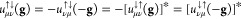
20The complex nature of *u*_*μν*_^*σσ*^(**g**) leads to a need for restructuring the calculation of the other contributions to the Fock operator, as is explored in the following section.

### 2.3 Hamiltonian Operator in a Pauli Spinor Basis

For the 2c-SCF program, generally, we consider the following class of Hamiltonian operators:
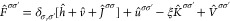
21ain which *V̂*^*σσ*′^ is defined as one of
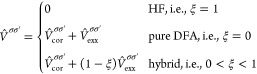
21bfor exchange and correlation (xc) potentials *V̂*_cor_^*σσ*′^ and *V̂*_exx_^*σσ*′^ from an as-of-yet unspecified density functional approximation (DFA), see Sections 2.4 and 2.6 for more details. In eq 21, *ĥ* is the scalar monoelectronic valence operator (containing the valence electronic kinetic and electron–nuclear terms), and *v̂* and *û*^*σσ*′^ (if included in the calculation) are the AREP and SOREP operators of Section 2.2. *Ĵ*^σσ^ and *K̂*^*σσ*′^ are the Coulomb and Fock exchange operators (ξ is the dimensionless *global* fraction of Fock exchange). As we see, if written in terms of Pauli spinors, this leads to so-called “generalized Hartree–Fock” and “2c DFT” approaches.^69^ For the Coulomb operator,
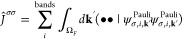
22aand for the Fock exchange operator,
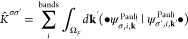
22bwhere Ω_*F*_ is the subvolume inside Ω for which band energies are below the Fermi level ϵ_*i*_(**k**) < ε_*F*_. In eq 22, we have made use of Mulliken shorthand notation for bielectronic integrals, and in eq 22a, for instance, the bullet points are interpreted in the sense that their matrix-elements read, in a Bloch function basis,
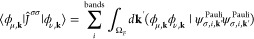
23By inserting eq 22 in eq 21 and using also eqs 8 and 10, it is possible to express the matrix elements of the Hamiltonian in the Bloch function basis *F*_*μν*_^*σσ*^(**k**) as an inverse Fourier transform of the corresponding matrix in the AO basis *F*_*μν*_^*σσ′*^(**g**),
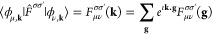
24where Hermitian matrix elements of the Hamiltonian (and any other) operator are defined as in eq 15 in an AO basis,
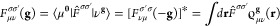
25To reduce the cost of constructing *F*_*μν*_^*σσ*^(**g**), the following direct-space Hermiticity relations are exploited, along with those already discussed in eqs 19 and 20. For *A*_*μν*_^*σσ*^(**g**) = *h*_*μν*_^*σσ*^(**g**) or *J*_*μν*_^*σσ*^(**g**) or *v*_*μν*_^*σσ*^(**g**),
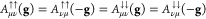
26For the xc potential,
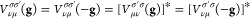
27while for the Fock exchange operator, the only relation is
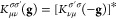
28In furthering the analysis, it is expedient to write out the Coulomb and exchange matrices in the AO basis by introducing the *complex* single-particle density matrix,
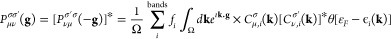
29where θ is the Heaviside step function, ε_*F*_ is the Fermi energy, and 0 < *f*_*i*_ < 1 is the fractional occupation of band *i*. It is convenient to also introduce the following compact notation for linear combinations of spin-blocks of **P**(**g**):
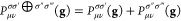
30aand
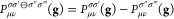
30bThen, using eqs 22, 25, 29, and 30, the Coulomb AO matrix is written as
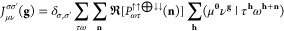
31while the exchange AO matrix reads
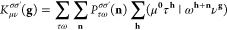
32and in eqs 31 and 32, we have again made use of the Mulliken shorthand notation for bielectronic integrals. The Coulomb AO integral of eq 31, for instance, is invariant to the following permutations of the AOs:
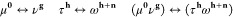
33where the first of these permutations, for instance, expresses the equivalence of the following two integrals:
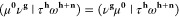
and likewise for the other two permutation relations of eq 33. The first two permutational symmetries in eq 33 are imposed through the Hermiticity of the Coulomb, exchange, and density matrices in eqs 26, 28, and 29. The third permutational symmetry of eq 33 must be imposed when contracting AO bielectronic integrals with the density matrix in eqs 31 and 32. In the present implementation, this contraction is performed independently for the Coulomb and exchange series. All permutational symmetries in eq 33 are exploited for the exchange term, but the third one is not used for the Coulomb term, for reasons that are explained in the paragraph that follows.

As in the SR program, the Coulomb series in the 2c-SCF program is evaluated using a scheme based on Ewald summation and by approximating the electrostatic Coulomb potential by a distributed point multipole model, as explained in ref (70). In this scheme, explicit bielectronic integrals as in eq 31 are only needed if the overlap between
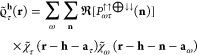
and the adjoined Gaussian overlap distribution  of eq 18 is smaller than a preset tolerance 10^–T2^ (see Crystal manual and ref (70) for a more ample discussion). Thus, most of the bielectronic integrals that would be generated by the third permutation in eq 33 are not needed for the Coulomb series, and this permutation has been disregarded entirely in the present implementation.

**Figure 2:**
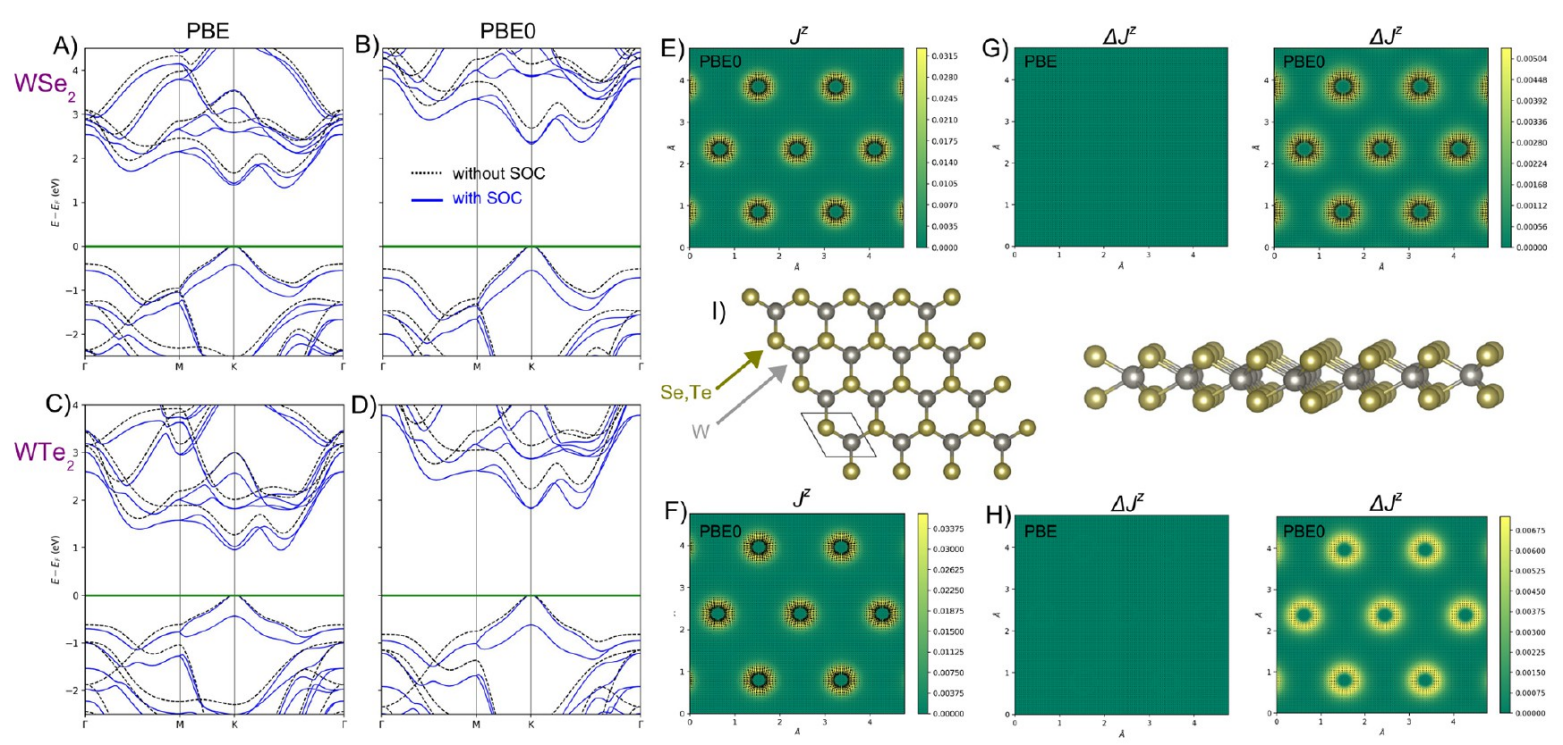
Electronic band structures of W-dichalcogenide monolayers for (A, B) WSe_2_ with the PBE and PBE0 functionals (C, D) WTe_2_ with the PBE and PBE0 functionals. (E, F) PBE0 *z* component spin-current densities (I) 2D W-dichalcogenide structure. (G, H) *z* component spin-current density differences with respect to second variational values for PBE (center panel) and PBE0 (rightmost panel) xc functionals.

Figure 2(A–D) provides electronic band structures of WSe_2_ and WTe_2_ W-dichalcogenide monolayers with (solid blue) and without (dotted black line) SOC, employing the PBE (pure GGA) and PBE0 (hybrid GGA) functionals.^71,72^ The layers are composed of alternating W and chalcogenide atoms in a buckled-honeycomb arrangement, as shown in Figure 2I). Computational details are provided in Appendix A. The band structures display considerable splitting from SOC, especially for the PBE0 hybrid functional calculations. For instance, the splitting of the valence band at Γ for WSe_2_ is doubled (nominal values of ∼0.125 eV with PBE and ∼0.25 eV with PBE0) when including a fraction of exact Fock exchange in the functional. This is no coincidence, as is explained in Sections 2.4 and 2.5 and is rationalized through a theoretical framework known as spin-current DFT (SCDFT).

### 2.4 Spin-Current Density Functional Theory

The Hohenberg–Kohn density functional theory for a Fermionic system in a Coulomb external field (that is to say, a field associated with a scalar-multiplicative potential *v̂*_*ext*_) shows that the energy can be expressed as a unique functional of the electron density ρ,^73^
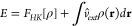
34where *F*_*HK*_ is the universal Hohenberg–Kohn functional. A treatment of more complex external fields leads to a dependence of the energy functional on a larger set of density variables, as first shown by Vignale and Rasolt, for the case of magnetic fields.^74,75^ With a magnetic field, the energy functional not only depends on ρ, but also on the three-dimensional magnetization vector **m**=[*m*_*x*_, *m*_*y*_, *m*_*z*_ ] and the particle-current density **j** (i.e., the current of the particles), leading to the so-called current-spin DFT.^74,75^

For SOC, it was similarly shown in recent years that the energy is a unique functional of ρ, **m**, **j**, but also the three currents of the three Cartesian components *m*_*x*_, *m*_*y*_, *m*_*z*_.^76−78^ These new density variables are the so-called spin-current densities **J**^*x*^, **J**^*y*^, and **J**^*z*^, leading to the spin-current DFT (SCDFT).^76−78^ The eight variables of the SCDFT are each related to one of the eight spin-blocks of the complex single-particle density matrix,^67,79^
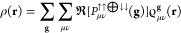
35a
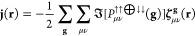
35bwhere the current overlap distribution was defined in eq 17, and the corresponding expressions for the magnetization and spin-current densities are provided in Appendix B.

The need for spin-current densities for a treatment of SOC is readily observed from the energy expression originally derived by Bencheikh for a Fermionic system with SOC in an arbitrary external static electromagnetic field,^76^
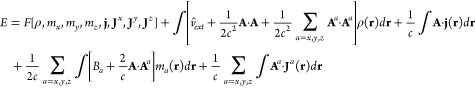
36where *B*_*a*_ is a Cartesian component of the external magnetic field intensity **B** =***∇*** × **A**, associated with the 3 × 1 vector potential **A**. *c* is the speed of light, and *F* is the universal SCDFT functional, including noninteracting kinetic *T*_*s*_, as well as Coulomb *J* and exchange-correlation *E*_*xc*_ energy contributions,
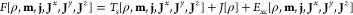
37The **A**^*a*^ symbols in eq 36 are vector potentials associated with SOC. In the absence of a magnetic field, **A**^*a*^ symbols are related to the SOC operator *û* through the relation^76^
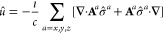
38where σ̂^*a*^ are the complex 2 × 2 Pauli spin matrices. In other words, **A**^*a*^ are 3 × 1 vector potentials that are defined by a particular (albeit somewhat unusual) way of writing the SOC operator through eq 38.

The presence of the last term in eq 36 shows that the spin-current densities **J**^*x*^, **J**^*y*^, and **J**^*z*^ are necessary (along with the electron density ρ) for writing the energy (and hence also the universal functional *F*, see refs (77 and 78) for an explicit demonstration) in the presence of SOC potentials **A**^*a*^. On the other hand, the particle-current density **j** and magnetization **m** only appear in eq 36 through coupling with the vector potential **A** associated with an external magnetic field. Hence, strictly speaking, **m** and **j** are only necessary for a calculation with an external magnetic field. For a field-free 2c-SCF calculation with SOC, the minimal set of density variables that should enter the functional *F* only comprises the electron density ρ and the three spin-current densities **J**^*x*^, **J**^*y*^, and **J**^*z*^. Nonetheless, for open-shell (i.e., time-reversal symmetry breaking) electronic states, **j** and **m** can be nonvanishing, even in the absence of a magnetic field. Therefore, it is still beneficial to include **j** and **m** in the energy functional *F* for field-free 2c-SCF calculations on open-shell systems.

This situation is analogous to a treatment of open-shell systems in spin DFT (SDFT).^80^ Although not formally required to include **m** in the energy functional for a calculation on open-shell systems without a magnetic field, it is beneficial, because open-shell systems carry a nonvanishing **m**.

### 2.5 Spin-Current Density Functional Theory Made Practical

In Crystal, SCDFT calculations are made possible through hybrid xc functionals.^79,81^ In this case, the xc energy *E*_*xc*_ of eq 37 has the general form^79^
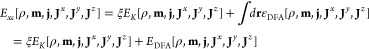
39where ε_DFA_ is the xc energy density from a semilocal DFA of the SCDFT, and *E*_*K*_ is the SCDFT Fock exchange energy, in a 2c spinor basis. As discussed in refs (77) and (79), eq 39 can be reduced to the following expression by exploiting the short-range behavior of the exchange hole, in the LDA or GGA of the SCDFT:
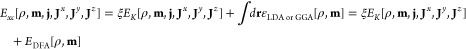
40which permits one to include particle- and spin-current densities in the xc energy expression, using standard DFAs of the SDFT.

In the present implementation in Crystal23, it is also possible to compare the results of a 2c-SCF SCDFT calculation with the analogous SDFT calculation, by using instead an energy expression like^81^
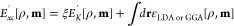
41where the only difference between eq 40 and eq 41 is that in eq 41*E*_*K*_^′^ is the SDFT (instead of SCDFT) Fock exchange energy in the 2c spinor basis, meaning that it is built from only the electron density ρ and magnetization **m**, but does not depend on the current densities. A comparison of predictions from eqs 40 and 41, using the keywords SCDFT and SDFT, allows one to study the effect of including current densities in the DFA on calculated properties from the 2c-SCF program.

**Figure 3:**
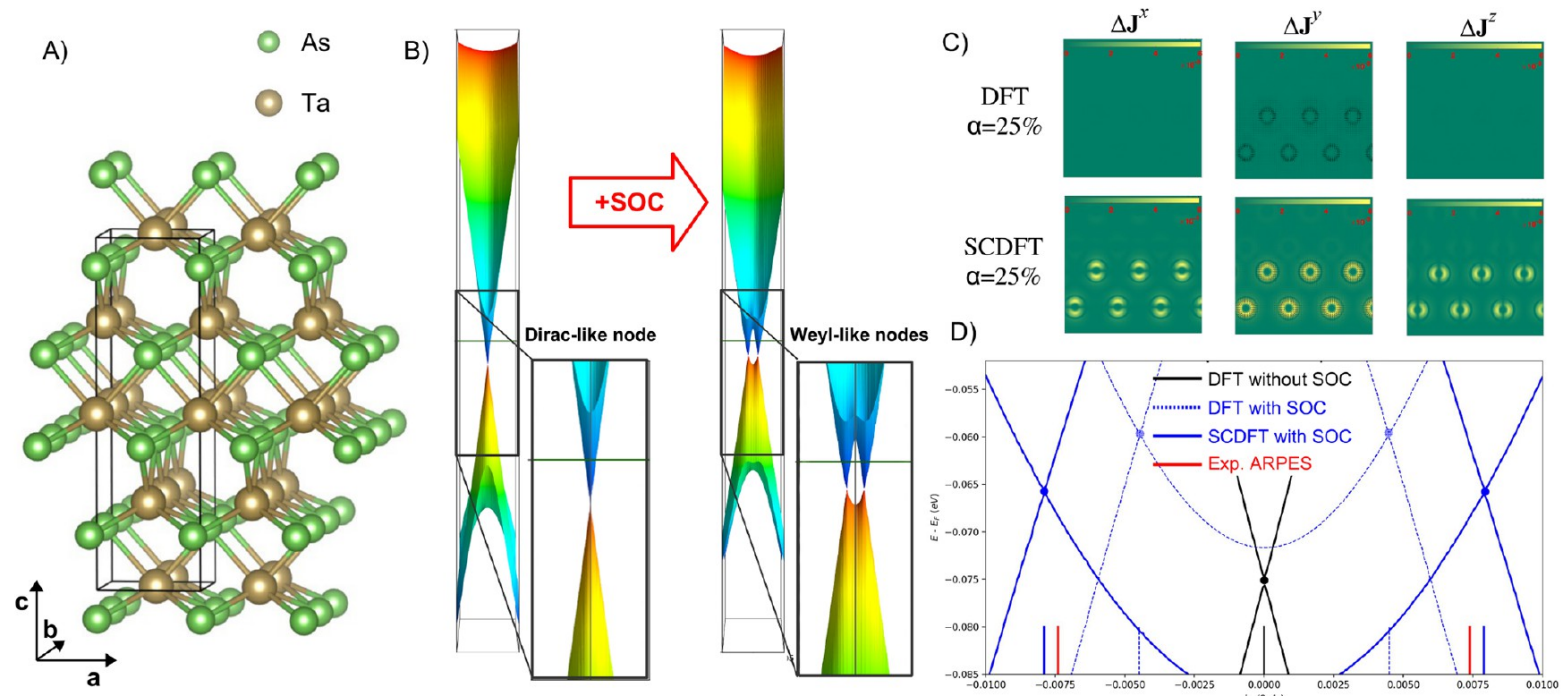
(A) Crystal structure of the *I*4_1_*md* tetragonal phase of TaAs. (B) Effect of SOC on the valence band structure. (C) Orbital-relaxation contribution to the spin-current densities Δ**J**^*i*^ = **J**_final_^*i*^ – **J**_initial_^*i*^, with differences taken with respect to second variational values in the DFT (upper panels) and SCDFT (lower panels). (D) Splitting by SOC of the Dirac-like node into Weyl node pairs in the DFT and SCDFT and comparison with ARPES experimental values.^81^

An example comparison of predictions from eqs 40 and 41 has been reported by Bodo et al.^81^ for band-structure calculations on the TaAs Weyl semimetal. The full input decks are provided in the Supporting Information.^82^ The results are summarized in Figure 3. The lack of inversion center in the *I*4_1_*md* TaAs crystal structure (Figure 3A) results in spin-splitting of the bands by SOC, whose effect on the valence band structure is shown in Figure 3B). Without SOC, the doubly degenerate valence and conduction bands converge toward a single point, with a 3D linear dispersion relation, forming a Dirac-like node. Spin-splitting of the Dirac-node by SOC results in the appearance of a pair of Weyl nodes. Experimental measurements, using angle-resolved photoemission spectroscopy (ARPES), provide an estimated splitting of about 0.015 (in units of 2 π/*a*, see red markers in Figure 3D) of the Weyl node pair. SDFT calculations, employing an xc energy expression as in eq 41, grossly underestimate this splitting at about 0.008 (dashed blue markers in Figure 3 D). In contrast, the SCDFT calculations, using instead the energy formula of eq 40, predict a splitting of 0.016, in excellent quantitative agreement with the experiment. This important difference in the SCDFT and SDFT calculations is rationalized with the help of Figure 3C), which provides color maps of the orbital-relaxation contribution to the spin-current densities of TaAs. The figure shows that significant spin current densities are accumulated along the self-consistent field process in the SCDFT calculations, resulting in a renormalization of the SOC potential and corresponding enhanced spin-splitting of the bands. In contrast, the SDFT calculations (top panels of Figure 3C) are completely unable to account for orbital relaxation of the spin currents, leading to a poor comparison against the ARPES experimental data.

### 2.6 Noncollinear Spin Density Functional Theory

In eq 40, the SCDFT xc energy requires a contribution from an explicitly paramatrized DFA of the SDFT. The functional derivatives of this second term *E*_DFA_ in eq 40 leads to the xc potential operator of eq 21,^32^
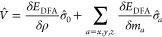
42where V̂ is the 2 × 2 xc potential operator in a 2c spinor basis, with elements *V̂*^*σσ*′^, as in eq 21. In this section, dependence of ρ(**r**), *m*_*a*_(**r**), and derived quantities on the coordinates of an electron **r** is dropped for notational convenience. Wherever integrals over **r** are used, it is assumed that all quantities contained within the integral and preceding *d***r** depend on **r**.

Different SDFT formulations are possible, depending on the specific details used to calculate the functional derivatives with respect to magnetization Cartesian components in eq 42. Two distinct strategies for LDA functionals have been implemented in Crystal23, while three strategies are available for GGA functionals. In closed shell systems (i.e., those systems that maintain time-reversal symmetry), the second term in eq 42 is vanishing, and all possible formulations coincide.

#### 2.6.1 Collinear Approach

The first (and simplest) formulation for both LDA and GGA functionals is the collinear one (*V̂* = *V̂*^*col*^), in which functional derivatives are only calculated with respect to the *z* component of the magnetization, thus,
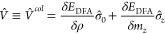
43In such a collinear formulation, *V̂*^*col*^ is block diagonal, that is to say *V̂*^*σσ*′^ = δ_*σσ*′_*V̂*^*σσ*^. In the LDA,^32^
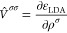
44in which  and .

In the GGA, it turns out to be convenient to not work directly with *V̂*^*σσ*^, but rather with its matrix elements *V*_*μν*_^*σσ*^(**g**) in an AO basis, which read^32^
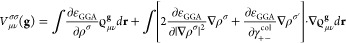
45where the overlap distribution ϱ_*μν*_^**g**^ has been defined in eq 16 and^32^
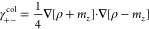
46The major disadvantage of the collinear formulation described by eqs 44 and 45 is that it leads to a total energy formula which is not rotationally invariant if a SOC operator is included in the Hamiltonian. That is to say, in a collinear calculation with SOC, the energy of an open-shell system will depend on its orientation in space.

#### 2.6.2 Noncollinear Approach

To solve the rotational invariance problem, it is necessary to adopt a noncollinear formulation of V̂ that includes functional derivatives not only with respect to *m*_*z*_, but also *m*_*x*_ and *m*_*y*_. Both the canonical noncollinear formulation of Kübler et al.^83^ as well as the noncollinear formulation of Scalmani and Frisch (SF)^84^ have been implemented in Crystal23. These two formulations coincide in the LDA, but differ in the GGA. The canonical formulation has the advantage of being conceptually simpler, while the SF formulation is slightly more numerically stable.

In the noncollinear formulations, *V̂* is no longer block diagonal in spin space. In the LDA, the xc potential operator reads^32^
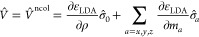
47while in the GGA, it is again convenient to work with matrix elements of *V̂*, which may be expressed as follows in terms of quantities defined in eq 42 and the matrix-element notation first introduced in eq 25. For the functional derivative with respect to ρ,^32^
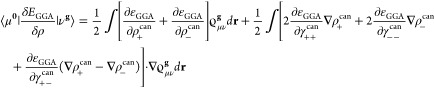
48aand for the functional derivative with respect to magnetization components,^32^
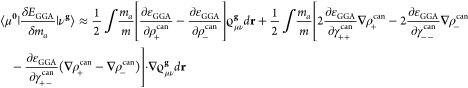
48bwhere we have introduced the following quantities proper to the canonical noncollinear formulation:
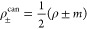
49aand
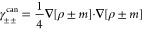
49bin which  is the modulus of the magnetization vector **m**. In eq 48b, an approximated equal sign has been used, because contributions originating from the gradient of *m*_*a*_/*m* have been dropped, which corresponds to assuming that the gradient of the magnetization locally follows the direction of the magnetization itself. In the end, the canonical noncollinear formulation of LDA and GGA functionals, described by eqs 47–49b, is similar to the collinear formulation of eqs 43–46, with the key difference being that *m*_*z*_ has been replaced in the energy functional by the vector modulus of the magnetization *m*,
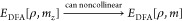
50This means that, while in the collinear approach the spin-quantization axis is everywhere fixed along *z*, in the canonical noncollinear formulation the spin-quantization axis at point **r** is locally defined along the direction of **m**(**r**) . This allows one to ensure rotational invariance of the total energy with a SOC operator in the Hamiltonian.

**Figure 4:**
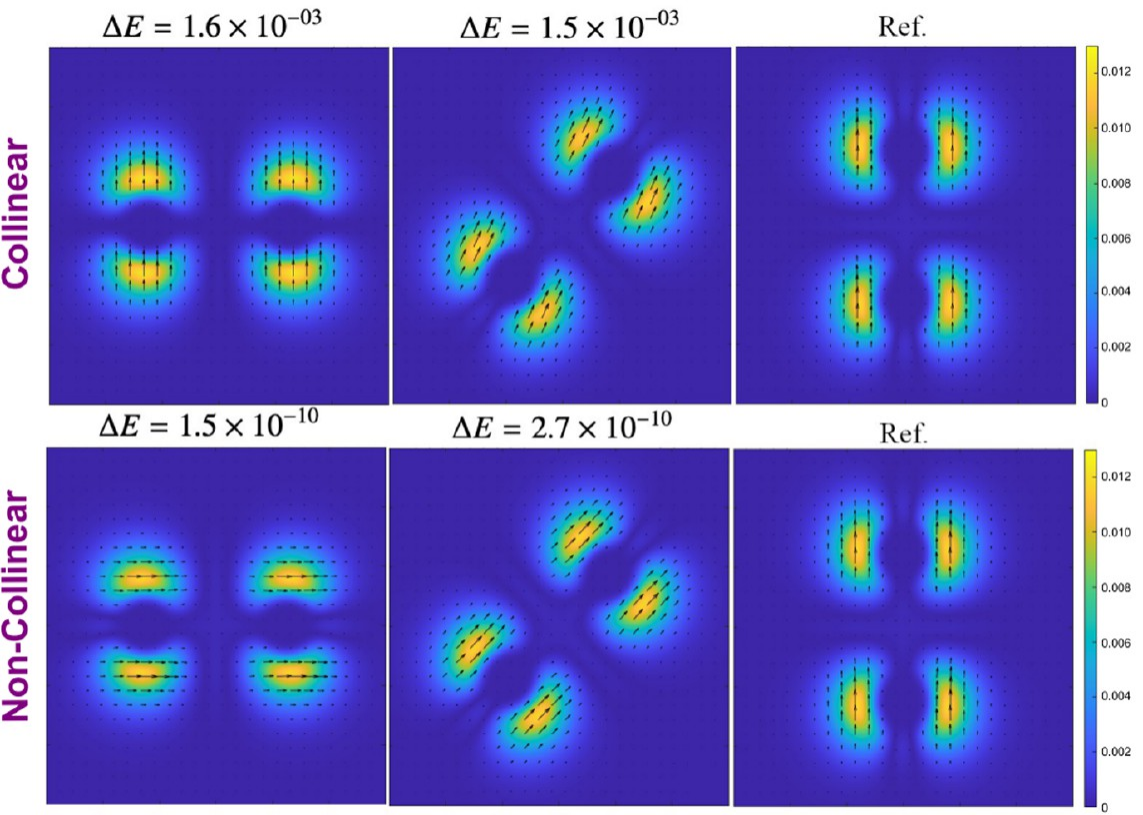
GGA (top panels) collinear and (bottom panels) noncollinear magnetization densities of the *I*_2_^+^ molecule, as it is rotated from the *x* axis to the *z* axis. Energy differences *ΔE* (in Hartree) with respect to the *z*-oriented molecule are also provided. The color intensity represents the magnitude , while the arrow length and direction represent the in-plane components *m*_*x*_ and *m*_*z*_.

Referance (32) quantified the restoration of rotational invariance of the total energy as provided by the noncollinear GGA formulation. The results are summarized in Figure 4, which shows color maps of the magnetization distribution for an *I*_2_^+^ molecule, as the molecular axis is rotated from the *x* direction to the *z* direction. In the top panels (referring to collinear GGA calculations), the missing dependence of the xc functional on *m*_*x*_ and *m*_*y*_ results in magnetization distributions being rotated away from the molecular axis for those orientations not coinciding with the *z* Cartesian direction. This results in significant energy differences on the order of 1 × 10^–3^*E*_*h*_ for different orientations of the molecule. In the present noncollinear GGA implementation (bottom panels), such energy differences are reduced down to an order of 1 × 10^–10^*E*_*h*_, thus confirming a nearly perfect rotational invariance of the total energy.

The canonical noncollinear formulation has been criticized for use with functionals beyond the LDA.^84^ Indeed, special care must be taken for implementation of the canonical formulation, because of the presence of delicate *m*_*a*_/*m* terms that appear in eq 48b, as well as 49b, is associated with a calculation of ***∇****m*. An alternative noncollinear formulation was proposed by SF and employs a different definition of the density gradient variables in eq 49b, which allows one to mitigate some of these difficulties for GGA functionals, leading to a slightly more stable numerical implementation.^84^ Both noncollinear formulations coincide in the LDA, but differ in the GGA. The SF formulation has also been implemented in Crystal23 for GGA functionals. Details on the implementation are available in ref (32).

#### 2.6.3 Technical Aspects of the Noncollinear Implementation

Some technical points to remember for noncollinear DFT calculations with the present implementation are the following:

1. In the current implementation, calculations with the canonical or SF noncollinear formulations are only possible using unpruned (i.e., uniform) integration grids. The default grid for noncollinear DFT calculations makes use of Gauss-Legendre radial and Lebedev angular point distributions and contains 75 radial points, as well as 974 angular points (i.e., an angular accuracy level of 16).^85−87^
2. A screening algorithm has been developed for dealing with those terms in eq 48b, as well as the analogous expression proper to the SF formulation, that contain *m*_*a*_/*m*, which is undefined at those points on the integration grid where the magnetization is vanishing. This procedure, described in ref (32), has been documented to provide rotational invariance of the total energy down to 1 × 10^–9^*E*_*h*_ for both the canonical and SF noncollinear formulations, employing GGA functionals, with SOC. Briefly, the screening procedure evaluates *m*_*a*_/*m* explicitly only if at least two Cartesian components of **m** exceed (in absolute value) a preset tolerance TOLM (the default value being 1 × 10^–27^ a.u.). If instead only one Cartesian component exceeds TOLM in absolute value, then the procedure reduces to the collinear problem along the corresponding component. Finally, if none of the Cartesian components of **m** exceeds TOLM, then terms proportional to the magnetization itself are set to zero in eq 48b (or its analogue in the SF formulation), whereas terms proportional to the gradient of the magnetization are calculated using a value *m*_*a*_/*m* →⟨*m*_*a*_/*m*⟩ which is averaged over the atomic basin in which the relevant point in the DFT grid is situated.^32^
3. The previously cited figure of 1 × 10^–9^*E*_*h*_ for rotational invariance of the total energy with noncollinear GGA functionals was only obtainable using a dense integration grid containing 500 radial points and an angular accuracy level of 29. With the default integration grid, on the other hand (containing only 75 radial points and an angular accuracy level of 16), rotational invariance on the total energy was achieved only to around 1 × 10^–7^*E*_*h*_, with slightly more accurate results using the SF formulation. Thus, generally very dense uniform grids are required for highly accurate noncollinear GGA calculations. It can be expected that much denser grids with respect to those typically used in collinear or closed-shell calculations are necessary for accurate noncollinear results on open-shell systems in a 2c-SCF with GGA functionals.
4. Finally, we note that the present implementation, based on a single-determinant KS wave function, is insufficient for many open-shell systems. Indeed, the present KS-DFT treatment is inappropriate for those so-called “strongly-correlated” open-shell systems, whose electronic densities are not pure-state *N*-representable, and would thus require an ensemble DFT treatment, which is still under development.^88−94^ If 2c-SCF calculations with SOC on such strongly correlated systems were performed, difficulties in converging the self-consistent procedure are expected (see ref (31) for examples of such difficulties, in which thousands of 2c-SCF cycles are required for convergence). In such difficult cases, a second-variational treatment (rather than self-consistent) of SOC is recommended, in which only one 2c-SCF cycle is performed, starting from a SR wave function, as a starting guess (keyword 2NDVARIAT).

#### 2.6.4 Starting Guess for the 2c-SCF Procedure

The following options are available as a starting guess for the 2c-SCF calculation:

1. GUESSPAT: Guess from a superposition of scalar-relativistic atomic densities (default)
2. GUESSPATNC: Guess from a superposition of scalar-relativistic atomic densities with noncollinear magnetization
3. GUESSPNOSO: Guess from a previous 1c-SCF calculation
4. GUESSPSO: Guess from a previous 2c-SCF calculation
5. GUESSROTM: Rotate the magnetization in the starting guess from a previous 1c-SCF or 2c-SCF
6. GCOREROT: Core Hamiltonian guess with infinitesimal magnetization along a selected direction

As in the SR program, the default starting guess for the density matrix for a 2c-SCF calculation is obtained from a superposition of nonrelativistic or scalar-relativistic multiconfigurational HF atomic densities, using an approach very similar to the one described in ref (95).

If the guess is used for a noncollinear DFT calculation, then it may be desirable to set a guess for the magnetization on each atomic center with an arbitrary orientation (not just along the *z* axis, as in a 1c-SCF calculation). For this purpose a keyword GUESSPATNC allows one to rotate the guess magnetization on each atomic center from the *z* axis to an arbitrary orientation. This approach, described in ref (31), allows one to define an atom-specific local orientation for the guess magnetization **m**^guess^(*k*) by a rotation of the collinear *m*_*z*_(*k*) using polar θ_*k*_ and azimuthal ϕ_*k*_ angles at atom *k*,
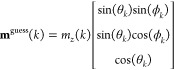
51Example applications of eq 51 to explore the rugged energy landscape in noncollinear DFT calculations are presented in ref (31).

The keywords GUESSPNOSO (GUESSPSO) allow one to use a density matrix from a previous 1c-SCF (2c-SCF) calculation as a starting guess for the 2c-SCF procedure. If the guess density matrix originates from a 1c-SCF, the parent calculation must however be unrestricted (through a use of the keywords UHF or SPIN) and have been performed without exploitation of space-group symmetry (for instance, using the keyword SYMMREMO).

Combination of the keyword GUESSROTM with any of the previously mentioned ones allows one to globally rotate the guess magnetization along a specified direction. This option can be used to estimate, for instance, the magnetic anisotropy energy (MAE).

Finally, the keyword GCOREROT permits one to use the core Hamiltonian as a starting guess (guess density matrix **P** = **0**), with an infinitesimal magnetization along a selected direction.

### 2.7 Cost of Relativistic vs Scalar-Relativistic Calculations

For the purposes of comparing the computational costs of 1c-SCF vs 2c-SCF calculations, a SR approach worth mentioning is the unrestricted Kohn–Sham (UKS) procedure, in which spin is imparted onto the Schrödinger wave function in an *ad hoc* way and is ubiquitous for a treatment of open-shell systems. The UKS procedure has been implemented in Crystal since around 1992^96−100^ and allows for a treatment of spin in an SR context, by a solution of the two uncoupled equations,
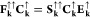
52a
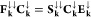
52bA comparison of the UKS and 2c-SCF procedures shows the apparent difference that eq 7 involves diagonalization of one large Fock matrix, while eq 52 requires diagonalizing two matrices of half the size. An estimate of the comparative costs of the calculations is expedient.

In the following analysis, we assume that diagonalization scales to the third power of matrix size and that the calculation is performed with *N* Bloch functions for every point **k** in the first Brillouin zone (FBZ). The key points in comparing costs of the calculation are the following:

- One solution of eq 7 scales as (2*N*)^3^ = 8 *N*^3^, whereas the cost of diagonalizing the UKS Fock matrices of eq 52 is only 2 *N*^3^. This yields a factor of 4, if the cost of the calculation is dominated by diagonalization.
- Exploitation of time-reversal symmetry in the solution of eq 52 halves the number of **k** points at which diagonalization must be performed, but not for eq 7. This further doubles the relative cost of diagonalization, bringing the factor to 8.
- In systems with nontrivial space-group symmetry, the cost of diagonalization of eq 52 is greatly reduced by limiting the number of **k** points to the irreducible wedge of the FBZ (IBZ) and by further factoring the Fock matrix at a point **k** in the IBZ into subblocks corresponding to irreducible representations of the group.^101−103^ In the 2c-SCF procedure of eq 7, on the other hand, presence of a SOC operator in the Hamiltonian means that the electronic wave function is imparted with double-group symmetry, rather than simple-group symmetry, which reduces the number of symmetry operators. Exploitation of such double-group symmetry is not implemented in the present version of the code, which can greatly increase the relative costs of calculations.
- In Crystal, the Bloch function Fock matrices are obtained by inverse Fourier transform of direct-space matrices **F**_**g**_^*σσ*′^, with elements *F*_*μν*_^*σσ*′^(**g**), calculated in an AO basis,
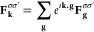
53In the UKS procedure, **F**_**g**_^*σσ*^ is pure real, but in the 2c-SCF procedure **F**_**g**_^*σσ*′^ is complex, leading to further additional costs, principally related to contraction of bielectronic integrals with a larger set of blocks of the density matrix in eqs 31 and 32.

In summary, for a calculation with no symmetry, it is expected that the new 2c-SCF procedure is roughly 1 order of magnitude more costly than a UKS Crystal calculation. However, for calculations exploiting symmetry in the solution of the UKS problem, the additional costs of performing the analogous 2c-SCF can largely exceed 1 order of magnitude.

## 3 Hybrid Density Functional Approximations and Composite Methods for Solids

The Crystal program has played a pioneering role in the field of hybrid DFT/HF approaches for extended systems,^14^ with some of the milestones being as follows: (i) The first implementation of the Fock exchange lattice series back in 1983,^104^ which then led to the first implementation of a periodic HF code (Crystal88), (ii) a first mixed DFT/HF approach for solids implemented back in 1987^105^ with an *a posteriori* correction to the HF total energy through the use of the Colle–Salvetti density functional (self-interaction corrected) for the correlation energy,^106^ (iii) in 1996, shortly after 1993 Becke’s original proposal,^107^ the implementation of global hybrid density functional approximations (DFAs) for solids,^108^ made available from the Crystal98 version, (iv) the implementation of a variety of screened-exchange DFAs (including the popular HSE06) distributed from the Crystal14 version,^109^ and (v) self-consistent hybrid DFAs, with the fraction of Fock exchange iteratively optimized through inverse proportionality to the dielectric tensor of the material, as originally formulated in 2014,^18^ implemented and made available in 2017.^19^

The combination of DFT and HF is also crucial in the formulation of Grimme’s hybrid DFT/HF composite methods.^110−112^ These methods were devised to enable affordable calculations and predict reliable geometries and energetics. The trade-off between accuracy and cost has been made possible by the well-balanced mixing of an adjusted double-ζ quality basis set and semiclassical corrections to cope with dispersion energy and to correct for the basis set superposition error (BSSE), thus providing results as accurate as more costly triple-ζ quality calculations.^113^ Since Crystal17, such composite methods were extended to periodic systems,^114^ but their applicability was limited to molecular crystals.

In this section, we present recent developments on hybrid DFAs and composite methods.

### 3.1 Implementation and Validation of Global Hybrid mGGA Functionals

A bunch of new mGGA functionals and related hybrid DFAs have been made available in the present release of the code. In detail, they include the following:

(i) DFAs derived from the B95^115^ mGGA correlation functional, namely, the B1B95^115^ method, which combines the B88 exchange functional and the B95 one as originally proposed by Becke, and the variants proposed by Truhlar and co-workers and others that use, instead, the mPW91^116^ exchange functional (i.e., MPW1B95,^117^ MPWB1K,^117^ PWB6K,^118^ and PW6B95^118^). All methods are hybridized with different amounts of Fock exchange ranging from 28% to 46%.
(ii) The highly parametrized semiempirical DFAs that belong to the well-known Minnesota family of functionals. The MN15^119^ hybrid functional and the related MN15L^120^ pure mGGA functional have been implemented along with the revised versions of the M06 and M06L ones (namely, rev-M06^121^ and rev-M06L^122^).
iii) The nonempirical, physically motivated, exchange-correlation functionals from Perdew and co-workers: in particular, the SCAN^123^ functional and its recently revised version r^2^-SCAN.^124^ Here, the r^2^-SCAN functional is considered in the global hybridized version with 25% of exact exchange.^125^

Before discussing the validation of the presently implemented mGGA functionals, we tested some of them to check their numerical accuracy and determine an integration grid that can be safely adopted for calculations with mGGA methods. The grid sensitivity of mGGA functionals is now well established, in particular, for molecular calculations.^126−130^ This originates from the form of the exchange-correlation functional and leads to numerical instabilities that can significantly affect the quality of the electronic total energy and in turn the potential energy surface.^128^ For most of the mGGA approximated methods, standard grids adopted for routine calculations are thus not suitable.^127^

**Figure 5:**
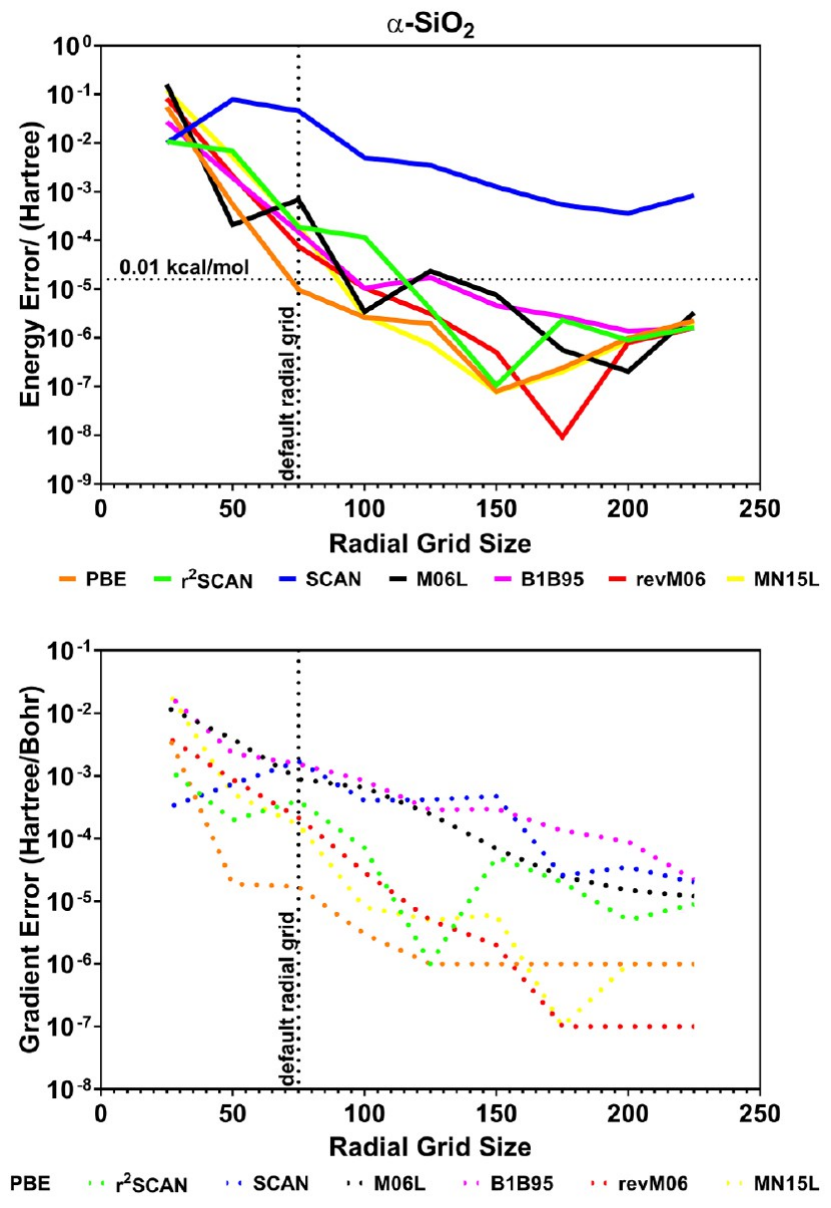
Error on the calculation of the electronic total energy (top) and the gradient norm (bottom) for α-quartz obtained by using different radial grid sizes at a fixed angular grid as evaluated for both GGA and mGGA functionals.

In Crystal, the numerical integration of the xc energy is based on an atomic partition scheme originally proposed by Becke^131^ for molecules and then extended to periodic systems.^132^ The atomic integration grids are comprised of a radial and an angular grid. The grid points are generated through a Gauss–Legendre radial quadrature and the Lebedev angular quadrature. In the trade-off between accuracy and cost, usually, a pruning scheme is employed to reduce the grid size.^133^ The integration grids can then be represented by two numbers (*n*,*m*) with *n* denoting the number of radial points and *m* the maximum number of angular points in the pruning scheme. The standard grid in the code is a (75,974) pruned grid (i.e., XLGRID). This grid size is accurate enough for SCF iterations and nuclear gradients of LDA and GGA functionals, but not for mGGA ones. Even the (99,1454) grid (i.e., XXLGRID) is not enough as can be seen from Figure 5. Indeed, Figure 5 shows the convergence of the electronic total energy (top) and the norm of the forces (bottom) for α-quartz as computed with a different number of radial points at fixed angular grid (1454) and pruning scheme. The plotted error is referred to energy and gradient norm computed with a (250,1454) grid. If one considers as threshold errors 10^–5^ Hartree and 10^–4^ Hartree/Bohr for energy and the gradient norm, respectively, it can be clearly seen that the default number of radial points (i.e., 75) gives the required accuracy for the PBE functional used as a benchmark for GGA functionals. Errors decrease by an order of magnitude for 99 radial points (XXLGRID), thus showing its numerical stability. In contrast, mGGA functionals suffer from a slower convergence as is particularly evident for the gradient norm. It is worthy to note that the SCAN and M06 functionals are clearly numerically unstable, while their revised and regularized versions show a more reliable behavior. Numerical stability tests on molecules (e.g., H_2_O, FeCp_2_) and other solids (e.g., MgO, Si, NiO) give similar results. Overall, for the tested mGGA functionals, at least 150 radial points are required to reach the given thresholds. Both energy- and gradient-related properties appear to be less influenced by the size of the numerical integration grid. For instance, with the r^2^-SCAN functional and the selected radial grid, the variations with respect to the reference grid for the optimized lattice parameters, vibrational frequencies, bulk modulus and elastic constants, and piezoelectric coefficients of α-quartz are on absolute average less than 0.001 Å, 0.3 cm^–1^, 0.1 GPa, and 0.01 pC/N, respectively. Accordingly, a new default grid of (150,1454) size has been set for calculations with mGGA functionals.

**Figure 6:**
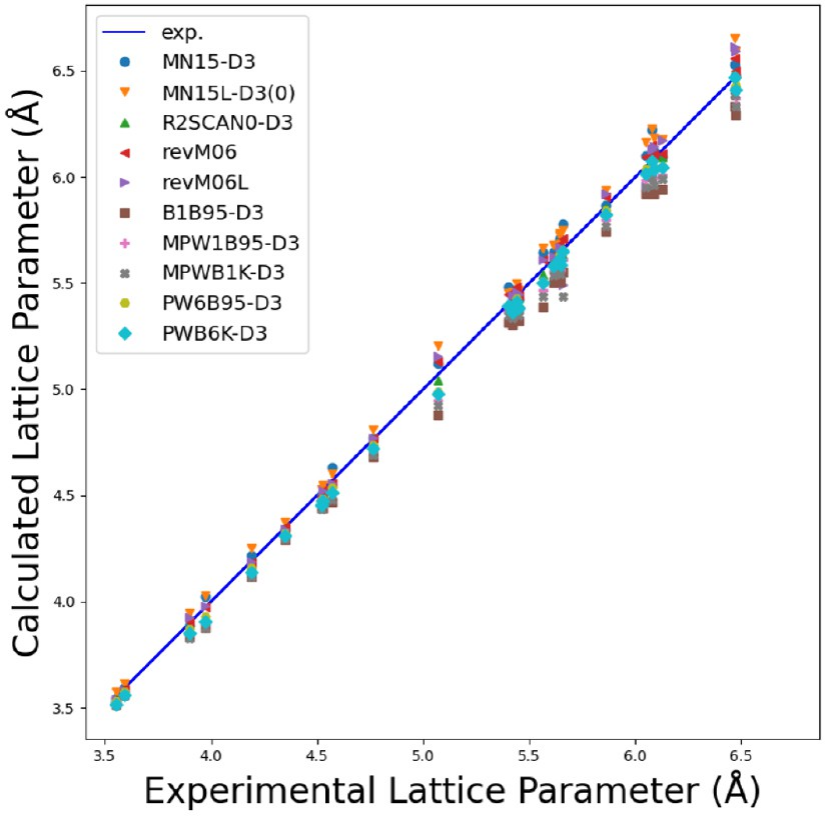
Comparison between computed and experimental lattice parameters for the 28 cubic crystals as per the newly implemented mGGA DFAs.

**Figure 7:**
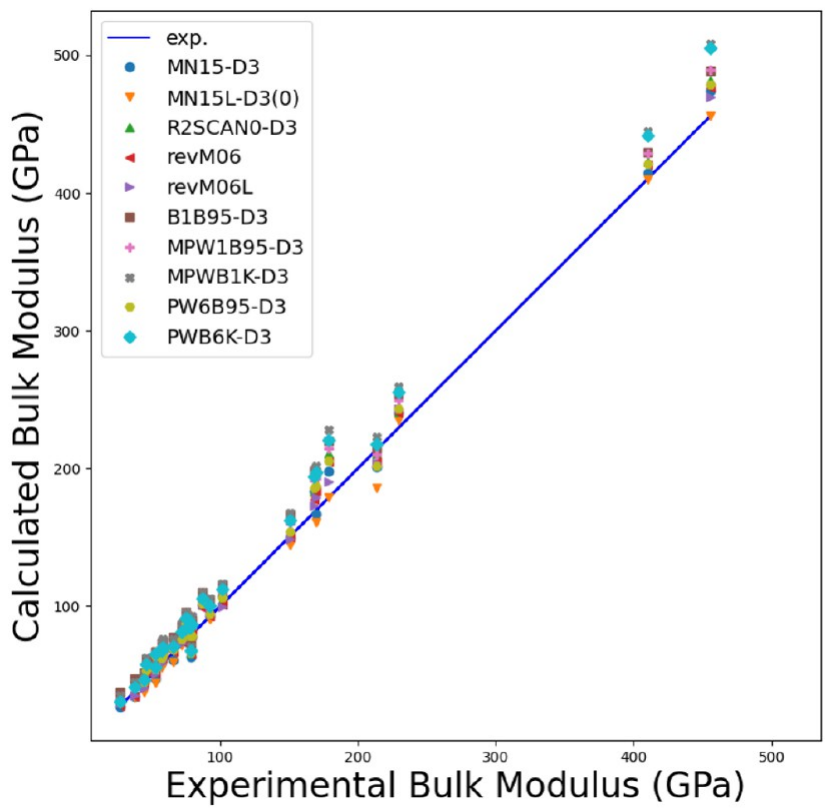
Comparison between computed and experimental bulk moduli for the 28 cubic crystals as per the newly implemented mGGA DFAs.

**Figure 8:**
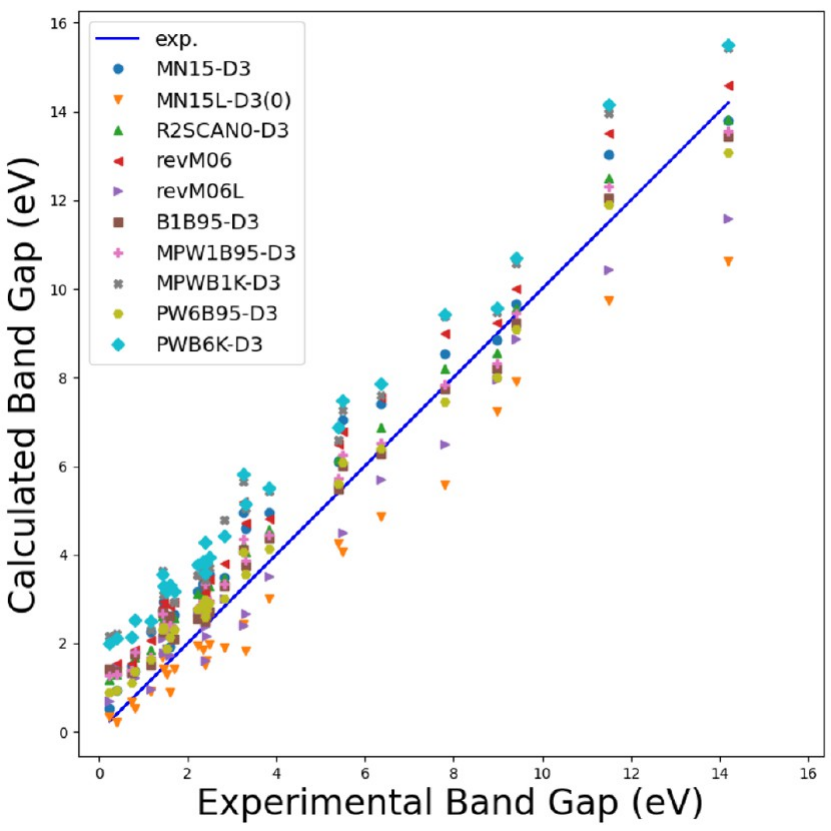
Comparison between computed and experimental band gaps for the 28 cubic crystals as per the newly implemented mGGA DFAs.

To validate the newly implemented DFAs, we analyze the lattice constant, bulk modulus, and band gap for a set of 28 crystals with cubic symmetry (semiconductors and insulators) and include 22 semiconductors, namely, C, Si, Ge, SiC, BN, BP, BAs, AlP, AlAs, AlSb, GaN, GaP, GaAs, GaSb, InP, InAs, InSb, ZnS-zb, ZnSe, ZnTe, CdTe, and MgS; four alkali halides LiF, LiCl, NaF, and NaCl; and two oxides MgO(B1) and SrTiO3(E21). Results are compared with a reference data set as collected in ref (134) for which low-temperature data, if available, and, when possible, the zero-point anharmonic expansion correction were included for a more consistent comparison. All DFAs were augmented with Grimme’s D3(BJ)^135,136^ dispersion correction with the Becke–Johnson damping function or the Chai–Head-Gordon zero-damping function, i.e., D3(0),^136^ except for the revised M06 and M06L functionals. See ref (134) for further computational details (e.g., basis sets) and references to experimental works. Figures 6, 7, and 8 show graphically the comparison between computed and experimental reference data, while Table 1 reports the mean absolute error (MAE) for the newly available mGGA methods for the predicted properties.

**Table 1:** Summary of results for mean absolute error (MAE) and standard deviation (in parentheses) of basic properties of 28 cubic crystals as computed with mGGA methods^a^

Method	%	LC (Å)	BM (GPa)	BG (eV)
PWB6K-D3	46	0.045 (0.023)	14.4 (11.8)	1.6 (0.4)
MPWB1K-D3	44	0.094 (0.037)	18.8 (11.9)	1.5 (0.4)
MN15-D3	44	0.041 (0.035)	6.7 (5.8)	0.9 (0.4)
revM06	40.41	0.027 (0.024)	6.9 (6.4)	1.1 (0.4)
MPW1B95-D3	31	0.064 (0.024)	12.7 (8.3)	0.7 (0.3)
B1B95-D3	28	0.112 (0.042)	16.5 (8.6)	0.6 (0.4)
PW6B95-D3	28	0.033 (0.023)	8.7 (6.9)	0.5 (0.3)
r^2^-SCAN0-D3	25	0.021 (0.013)	9.6 (7.1)	0.7 (0.2)
MN15L-D3(0)	0	0.067 (0.044)	6.5 (6.2)	0.9 (0.8)
revM06L	0	0.042 (0.044)	6.6 (5.5)	0.6 (0.5)

a The reported quantities include LC (lattice constant), BM (bulk modulus), and BG (band gap). For hybrid functionals, the percentage of Fock exchange (%) is also reported.

Reassuringly, from the validation point of view, results are in good agreement with the ones obtained for other functionals belonging to the fourth rung of Jacob’s ladder.^134,137^ Even though we are not interested in assessing the performance of the selected mGGA DFAs, Table 1 offers some useful insight on the behavior of the xc functionals as applied to solids. It is worthy to note that so far none of them have been tested in solid state calculations because of the well-known difficulty of using hybrid functionals in plane-wave codes, whereas they can be easily run with Crystal. From Table 1, it can be clearly seen that hybridization definitely improves the results, although pure mGGA functionals still give a good performance as for revM06L. Not unexpectedly, decreasing the amount of exact exchange below 30% leads to better band gap predictions. In particular, PW6B95-D3 shows remarkably good results and confirms its accuracy not only for molecules^138−140^ but also for solids. The recently proposed r^2^-SCAN functional as combined with 25% of Fock exchange provides good results, but probably band gaps can be further improved by slightly reducing the amount of exact exchange while keeping its overall accuracy for the other properties. Notably, although some of the newly available mGGA functionals have been devised for molecular calculations, they appear to be also suitable for solid state calculations (in particular, the hybrid ones), thus showing a broader range of applicability.

### 3.2 Extension of CPHF/KS Scheme to HJS Exchange Hole Model

In the present version of the Crystal code, the Coupled-Perturbed-Hartree–Fock/Kohn–Sham (CPHF/KS) scheme to evaluate the response to external electric fields^141−143^ has been extended to range-separated hybrid (RSH) functionals based on the Henderson–Janesko–Scuseria (HJS) exchange hole.^144^ The xcfun library^145^ of xc functionals used by the CPHF/KS scheme to compute second- and high-order derivatives of the functional has been modified accordingly. CPHF/KS calculations are available for long-range-corrected (LC-RSH), middle-range-corrected (MC-RSH), and short-range-corrected (SC-RSH) hybrid functionals of the general form
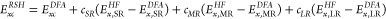
In particular, the current extension applies to some well-known RSH functionals, such as the HSE06 and HSEsol SC-RSHs, the HISS MC-RSH, and the LC-ωPBE and LC-ωPBEsol LC-RSHs. As an example, Table 2 reports the results of the calculation of the linear electric susceptibilities of the molecular crystal of *m*-nitroaniline (mNA) with the HSE06, HISS, and LC-ωPBE functionals, as compared to the B3LYP results and experimental data at different wavelengths.^146^

**Table 2:** Comparison between Experimental and Predicted First-Order Electric Susceptibility of mNA Molecular Crystal as Computed at Different Wavelengths of Electric Field (in nm), with Different Variants of Range Separated Hybrid Functionals Based on HJS x-Hole^a^

Functional (λ)	χ_*aa*_^(1)^	χ_*bb*_^(1)^	χ_*cc*_^(1)^
B3LYP (1064)	1.853	1.750	1.573
B3LYP (1319)	1.832	1.730	1.554
B3LYP (∞)	1.794	1.698	1.522
HSE06 (1064)	1.845	1.746	1.569
HSE06 (1319)	1.824	1.727	1.549
HSE06 (∞)	1.787	1.695	1.518
HISS (1064)	1.757	1.686	1.496
HISS (1319)	1.739	1.670	1.481
HISS (∞)	1.709	1.642	1.454
LC-ωPBE (1064)	1.633	1.637	1.377
LC-ωPBE (1319)	1.621	1.624	1.367
LC-ωPBE (∞)	1.600	1.602	1.351
Exp. (1064)	1.957	1.833	1.664
Exp. (1319)	1.929	1.809	1.637
Exp. (∞)	1.810	1.665	1.469

a Basis set: 6-311G(2df,2pd//6-311G(d,p).

The three RSH functionals contain more or less the same amount of Fock exchange but at different ranges. Interestingly, a clear trend is observed in the transition from short to long range with the values of χ^(1)^ decreasing systematically when passing from HSE06 to HISS and LC-ωPBE. Instead, the global hybrid B3LYP gives results similar to the HSE06 ones. B3LYP and HSE06 computed data are in better agreement with experiment than HISS and LC-ωPBE, in particular, at the static limit (i.e., λ = ∞) with an average deviation of less than 2%. At lower wavelengths, the agreement worsens with all functionals giving underestimated χ^(1)^ values. Overall, present results show that the extension of the HJS-based RSH methods to the CPHF/KS module offers a useful tool to understand the role of exact exchange in hybrid DFT/HF functionals, and in perspective, it would pave the path to develop optimally tuned RSH functionals for solids.

### 3.3 Revised Composite Methods for Solid State Calculations

In Crystal23, the composite methods originally proposed by Grimme (namely, HF-3c, PBEh-3c, and HSE-3c) have been revised with the goal of extending their applicability to inorganic solids, layered systems, and metal–organic materials. They share the same expression for the total energy provided by the original composite methods,
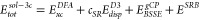
54that includes the semiclassical corrections exploited by the “3c” methods: the D3 dispersion correction, the geometrical Counter-Poise^147,148^ (gCP) correction for the basis set superposition error (BSSE) and incompleteness, and the short-range correction (SRB) for covalent bond lengths.

The main guidelines adopted for the revision strategy can be summarized as follows:

(i) use of exchange-correlation functionals developed for solids (i.e., PBEsol and HSEsol)
(ii) reduction of the amount of Fock exchange in DFT hybrid methods for a better description of electronic properties (e.g., 20%–25%)
(iii) application of a simple recipe to make molecular basis sets originally adopted by Grimme and co-workers suitable for inorganic solids. Indeed, the original composite methods make use of a minimal (MINIX^110^) and double-ζ quality (def2-mSVP^111,112^) atomic basis sets for HF and hybrid DFT methods, respectively, that are mostly unmodified molecular basis sets thus being not fully suitable for solid state calculations

In particular, the latter represents the most crucial modification to extend the applicability of the revised composite methods to a wider range of solid state systems. In detail, the recipe adopted is based on (i) an upshift of the exponents of the outermost Gaussian basis functions to a value equal or slightly greater than 0.1 Bohr^–2^ that has been considered as a lower bound limit to avoid numerical instability and (ii) scaling of the exponent of the previous Gaussian basis function by keeping the original exponent ratio.

**Figure 9:**
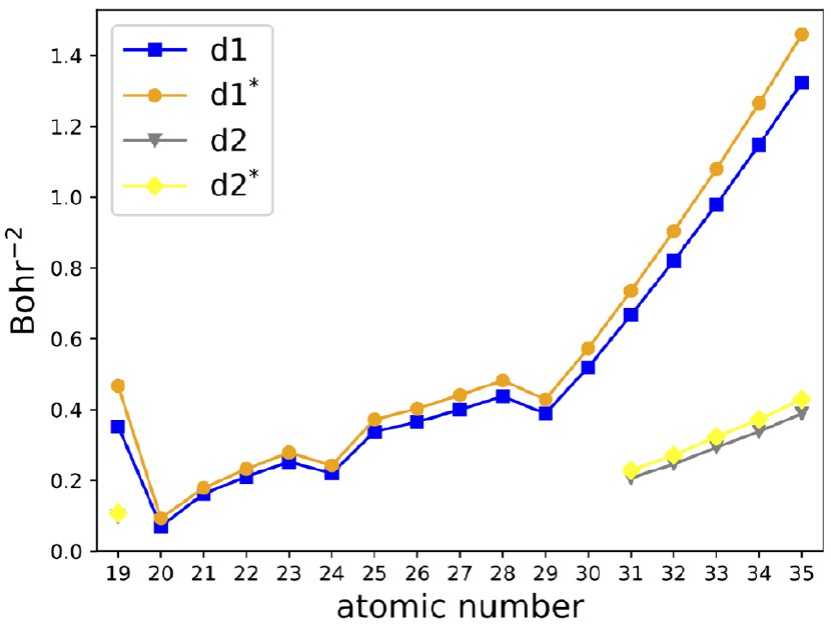
Example of revision of the def2-mSVP basis set: *d* exponents for the elements of the fourth row before (gray and blue lines) and after (yellow and orange lines) the basis set revision. *d*1 is the exponent in the inner Gaussian function, while *d*2 is the one of the outermost one.

**Figure 10:**
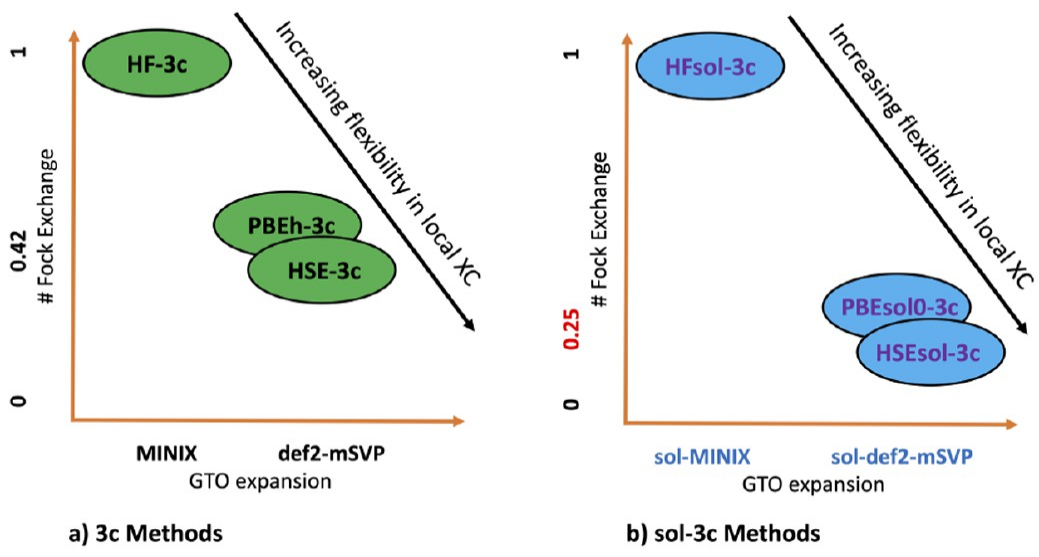
Comparison between the original 3*c* composite methods (a) and the revised sol-3*c* ones (b).

As an example, a graphical representation of the revision applied to the original def2-mSVP basis set for the *d* orbital exponents of the fourth-row elements of the periodic table is given in Figure 9. Basis set exponents have been revised from He to Xe for both def2-mSVP and MINIX basis sets. For the latter, the same procedure has been adopted, but from H to Ar the Guassian functions have been decontracted before applying the scaling. Further details on the revision of the basis sets can be found in the Supporting Information of ref (149). The revised methods have been tagged with a label “sol” (as for “solids”) to distinguish them from the original ones whose application was limited to molecules and molecular crystals. Accordingly, the resulting methods have been denoted as HFsol-3c, PBEsol0-3c, and HSEsol-3c. Figure 10 is a graphical representation of the main differences between the original 3*c* composite methods and the present sol-3*c* revised ones.

To show the wider applicability of the revised sol-3*c* composite methods, they have been benchmarked against the standard molecular adducts S66x8^150^ data set, the X23^151,152^ set of molecular crystals, and the SS20 set of solids (i.e., a subset of the 28 solids discussed above). Results are summarized in Table 3 in which the original 3*c* composite methods are also included for comparison. Of course, the latter cannot be applied to the SS20 set of solids for which the comparison has been extended to the parent methods without correction potentials. In ref (149), further tests have been reported for other inorganic systems, layered materials, and different properties. Overall, the revised sol-3*c* composite methods give comparably good or even better performance than 3*c* composite methods and uncorrected parent methods thus showing that they are well suited for a broad range of applications from molecules to solids. The wider applicability of sol-3*c* hybrid DFT/HF composite methods has been also demonstrated in the modeling of metal–organic frameworks (MOF).^153^ MOFs can represent a challenge in many respects because of their chemical versatility, modular nature, unit cell size, and complexity of the framework. The structural, vibrational, electronic, and adsorption properties were computed for some of the most common MOFs with excellent results. Furthermore, the PBEsol0-3c hybrid composite method has been successfully applied to corroborate experimental findings on the encapsulation of molecules in MOFs and zeolitic−imidazolate frameworks (ZIFs) for drug delivery (i.e., 5-fluorouracil)^154^ and for solid state lighting (i.e., fluorescein),^155^ respectively, and to elucidate the mechanical properties of defective ZIF-8.^156^ Finally, it is worth noting that in the trade-off between accuracy and cost, sol-3*c* hybrid DFT/HF composite methods have been demonstrated to be cost effective for calculations on physical systems with thousands of atoms on computational resources with a relatively small number of cores at a moderate cost in terms of CPU time and memory.^157^ Indeed, a striking agreement between experimental and computed structures (ΔV < 1%) has been reported for giant MOFs as MIL-100 and MIL-101 with about 2800 and 3600 atoms in the unit cell, respectively.^153^ The availability of the sol-3*c* composite methods in the Crystal code from the HFsol-3c to the hybrid DFT/HF ones thus allows one to tackle very large systems, providing cost-effective yet accurate results.

**Table 3:** Statistical Analysis of Results for Three Benchmark Sets (Namely, S66x8, X23, and SS20) as Obtained for Original 3*c* and Revised sol-3*c* Composite Methods and for Parent Methods without Correction Potentials^a^

Data set	Prop.		HF^*a*^	HF-3c	HFsol-3c	PBEsol0^b^	PBEh-3c	PBEsol0-3c	HSEsol^b^	HSE-3c	HSEsol-3c
S66x8	Dist.	MARE (%)	–	0.50	0.39	–	1.50	0.51	–	1.50	0.49
	BE	MAE (kcal/mol)	–	0.43	0.71	–	0.50	0.64	–	0.50	0.66
X23	Vol.	MARE (%)	–	6.46	2.31	–	3.60	3.18	–	2.90	2.84
	CE	MAE(kcal/mol)	–	2.06	3.03	–	1.30	1.53	–	1.30	1.50
SS20	LP	MAE (Å)	0.07	–	0.07	0.03	–	0.03	0.03	–	0.03
	BG	MAE (eV)	6.75	–	6.95	0.78	–	0.92	0.67	–	0.77
	BM	MAE (GPa)	22.05	–	26.70	9.34	–	7.93	8.96	–	7.63

a Mean absolute errors (MAE) and mean absolute relative errors (MARE) are reported for different quantities: Dist. (intermolecular equilibrium distance), BE (binding energy), Vol. (equilibrium volume), CE (cohesive energy), LP (lattice parameter), BG (band gap) and BM (bulk modulus).

b Used in the same basis set expansion as in the corresponding “sol-3c” methods, but without correction potentials.

## 4 Improvements to Saunders Algorithm for Calculation of Integrals

### 4.1 Extension of LCAO Approach to *g*-type Atomic Orbitals

In initial distributions of Crystal, integrals were only programmed up to *l* = 2 *d*-type Gaussians. The *l* = 3 *f*-type functions were made available for total-energy and gradient calculations around 2003, with Crystal03.^158^ Now in Crystal23, all calculations (total energy, gradients, vibrational frequencies, response properties, etc.) have been extended to *l* = 4 *g*-type functions.^159^

In extending Crystal to *g*-type functions, most of the work is related to evaluating new integrals (and their derivatives) according to the scheme of Saunders.^33^ It was mentionned through eq 10 that Bloch functions in Crystal are expressed as an inverse Fourier transform of AOs χ_μ_. More specifically, χ_μ_ are written as a linear combination of RSSH-GTFs ,
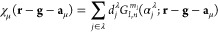
55where *d*_*j*_^λ^ and α_*j*_^λ^ are fixed coefficients and exponents of the shell λ, and *l*, *m*_*l*_, *n* are the usual azimuthal, magnetic, and principal quantum numbers. Each primitive RSSH-GTF  is defined, in turn, as a product of an RSSH  with a GTF,
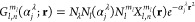
56where *N*_λ_, *N*_*l*_(α_*j*_^λ^), and *N*_*l*_^*m*_*l*_^ are shell and *m*_*l*_-(in)dependent primitive normalization factors, whose exact expressions are provided in ref (159). In eq 56, an RSSH  is a homogeneous Cartesian polynomial that reads
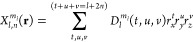
57in which the sum runs over all triplets of integers *t*, *u*, *v* that satisfy the equality *t* + *u* + *v* = *l*+2*n*.  are coefficients which can be tabulated from recurrence relations for  as explained in ref (160).

For what follows, it is useful to introduce the unnormalized RSSH-GTF *R*,
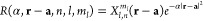
58In Crystal, only *R*(α,**r**,0,*l*,*m*_*l*_) (with *n* = 0) are used as basis functions. But *R*(α,**r**,*n*,*l*,*m*_*l*_) (with *n* > 0) are used in the calculation of kinetic energy integrals (and their derivatives).^33,161,162^ At variance with most other quantum chemistry programs, in Crystal, integrals are directly evaluated in the RSSH-GTF basis (not the Cartesian GTF basis), using a scheme originally described by Saunders in ref (33).

In the Saunders scheme, a pair product of RSSH-GTFs is expanded into so-called Hermite GTFs Λ,
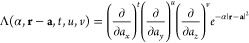
59The expansion of a pair product of RSSH-GTFs into Hermite GTFs is achieved through coefficients *E*,
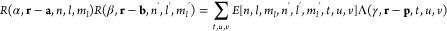
60where γ = α + β, and **p** is the centroid of the RSSH-GTF pair **p** = (α**a** + β**b**)/γ. The expansion coefficients *E* are zero for *t* < 0, *u* < 0, and *v* < 0, and in eq 60, the sum runs over all triplets that satisfy *t* + *u* + *v* ≤ 2*n* + 2*n*′ + *l* + *l*′.

The coefficients *E* are calculated using recurrence relations that may be derived from the corresponding recurrences for spherical harmonics and Hermite polynomials.^33,159^ However, in Crystal, these recurrence relations are not programmed themselves. Instead, the symbolic expressions for the *E* coefficients are tabulated up to a given value of *l* (up to *l* = 3 *f*-type functions from Crystal03–Crystal17 and now *l* = 4 *g*-type functions in Crystal23). Such an explicit tabulation is crucial from the point of view of computational efficiency, as is discussed in ref (159). New *l* = 4 *g*-type function routines were generated by tabulating the relevant *E* coefficients using computer algebra system (CAS) for symbolic computation available in Matlab, along with automated generation of Fortran77 routines. In passing, we note that a similar strategy (using instead the Maple CAS) had been used by Saunders and colleagues in their 1997 work for extending the evaluation of the Boys function derivatives to higher quantum numbers.^163^

The tabulation of explicit symbolic expressions for the *E* coefficients (and *G*^*a*^_*x*_, *G*^*a*^_*y*_, *G*^*a*^_*z*_ coefficients, see Section 4.2), rather than a direct use of recurrence relations, results in highly efficient routines, as is briefly discussed below. It allows, on the one hand, to exploit the sparsity of the coefficients (i.e., circumvent superfluous calculations of zeros) and, on the other hand, avoids the evaluation of a very large number of logical statements which would otherwise be required in the direct application of recurrence relations.^159^

**Figure 11:**
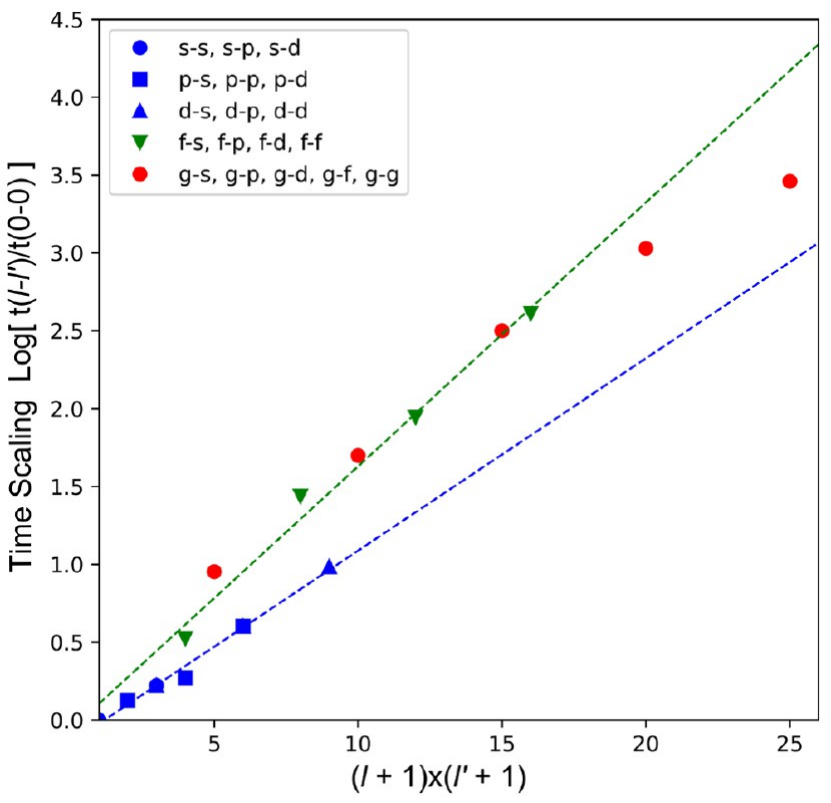
Computational efficiency of the different sets of routines for calculating the *E* RSSH-GTF pair coefficients of eq 60 for the total energy. The blue shapes represent timings for the oldest series of routines for calculating *s*-*s*, *s*-*p*, *s*-*d*, *p*-*s*, ..., *d*-*d* coefficients. The green triangles are for the *f*-*s*, *f*-*p*, ..., *f*-*f* coefficient routines introduced in Crystal03. The red dots are for the new *g*-*s*, *g*-*p*, ..., *g*-*g* coefficient routines of Crystal23.

The relative efficiency of the new *g*-type function routines for the *E* coefficients of eq 60 required for total energy calculations is documented in Figure 11. The subroutine that calculates the tabulated values of the *E* coefficients was called one million times for increasing values of *l* and *l*′. The logarithm of wall clock times is plotted as a function of (*l* + 1) × (*l*′ + 1). The blue shapes correspond to the oldest routines for *s*-*s*, *s*-*p*, *s*-*d*, *p*-*s*, ..., *d*-*d* coefficients, which are calculated through highly optimized routines. The green triangles are for the *f*-*s*, *f*-*p*, ..., *f*-*f* coefficient routines introduced in Crystal03. This green series follows a linear trend that lies above the blue trend, because the corresponding *f* orbital routines were generated from a slightly less efficient strategy. Indeed, these *f* orbital routines, written around 2003, do not make a direct use of the scheme proposed by eq 60 and are instead based on an expansion of Cartesian GTFs into Hermite GTFs, followed by a subsequent transformation to the RSSH-GTF basis, which explains their relative inefficiency.

The red dots in Figure 11 represent the new routines that were generated by the symbolic computation in Matlab for *g* orbitals, based on eq 60. These new routines plot in between the blue and green linear trends, indicating comparable efficiency to the previously existing routines for *s* to *f* orbitals.

Once the coefficients of eqs 60 and 63 are obtained from the tabulated symbolic expression, then the task of calculating integrals is straightforward. As an example, consider the overlap integral, using eq 60,^33^
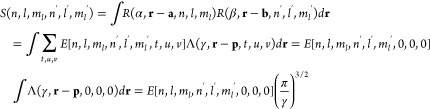
61where we have used the following relation which may be derived from the orthogonality of Hermite polynomials (see also p. 161 of ref (164)) followed by integration of an *s*-type Gaussian function in three dimensions,
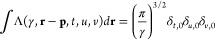
62Other integrals can be obtained similarly.^33,70^

### 4.2 Accelerated Calculation of Derivatives of Integrals for the Analytical Gradient

For calculating analytical gradients of the total energy, derivatives of the integrals with respect to nuclear displacements *a*_*x*_, *a*_*y*_, or *a*_*z*_, are required. These are calculated through coefficients *G*^*a*^_*x*_, *G*^*a*^_*y*_, *G*^*a*^_*z*_, in a similar way to the *E* coefficients of eq 60. That is, the derivative of a pair product of RSSH-GTF with respect to a displacement (*a*_*x*_, for instance) is expanded in Hermite GTFs through coefficients *G*^*a*^_*x*_,
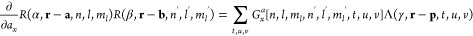
63in which the sum over triplets *t*, *u*, *v* runs over all values that satisfy *t* + *u* + *v* ≤ 2*n* + 2*n*′+ *l* + *l*′+1. Similar coefficients are also calculated for displacements of other Cartesian components with coefficients *G*^*a*^_*y*_ and *G*^*a*^_*z*_. As for the *E* coeffcients, the *G*^*a*^_*x*_, *G*^*a*^_*y*_ ,and *G*^*a*^_*z*_ coefficients are determined by increasing quantum numbers using recurrence relations. New formulas are only needed for increasing quantum numbers on the center for which the derivative is taken (i.e., center **a** in the case of the *G*^*a*^_*x*_, *G*^*a*^_*y*_, and *G*^*a*^_*z*_ coefficients). The recurrence relations for a calculation of *G*^*a*^_*x*_ were provided in ref (161), while those for *G*^*a*^_*y*_ and *G*^*a*^_*z*_ are provided in ref (159). Quantum numbers are increased on center **a** using eqs 33–36 of ref (161) for *G*^*a*^_*x*_ and eqs 17–24 of ref (159) for *G*^*a*^_*y*_ and *G*^*a*^_*z*_.

**Figure 12:**
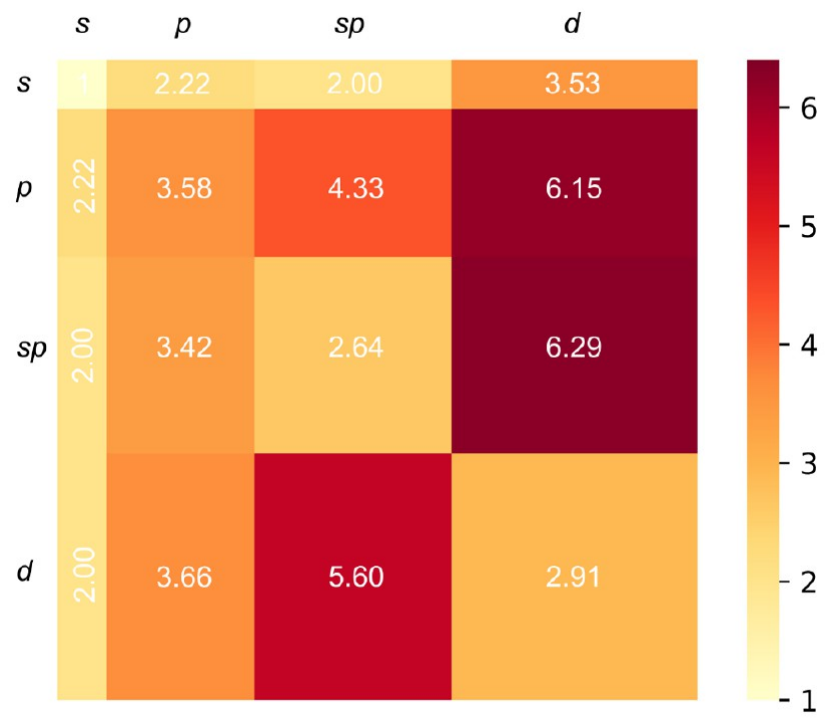
Speedups for calculating *s*-*s*, *s*-*p*,···, *p*-*s*, *p*-*p*,···, *d*-*d* RSSH-GTF pair *G*^*a*^_*x*_, *G*^*a*^_*y*_, and *G*^*a*^_*z*_ coefficients with the new routines in Crystal23 vs the previously existing routines.

In Crystal derivatives are always evaluated with respect to center **a**. Those with respect to center **b** in eq 63 can be obtained following eqs 37–42 of ref (161),
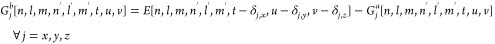
64In the same way as for the *E* coefficients, the computer algebra system for symbolic computation available in Matlab was used to calculate and generate explicit Fortran77 routines for the *G*^*a*^_*x*_, *G*^*a*^_*y*_, and *G*^*a*^_*z*_ coefficients (general up to *l* = 4 *g*-type functions). In the case of the gradient *G*^*a*^_*x*_, *G*^*a*^_*y*_, and *G*^*a*^_*z*_ coefficients, not only did the new routines permit an extension of analytical gradient calculations to *g*-type functions, but also they were found to be faster than the previously existing ones for *s*- to *d*-type functions. The computational savings afforded by the new routines for *G*^*a*^_*x*_, *G*^*a*^_*y*_, and *G*^*a*^_*z*_ coefficients is documented in Figure 12, which provides speedups of the new vs old routines for *s*- to *d*-type functions. The speedups are asymmetric (e.g., factor of 3.66 for *d*-*p* vs 6.15 for *p*-*d*) because of the derivative in eq 63, which is only taken on the left Gaussian function. In the best cases (*p*-*d* and *sp*-*d*), the relevant *G*^*a*^_*x*_, *G*^*a*^_*y*_, and *G*^*a*^_*z*_ coefficients are calculated over six times faster in Crystal23, compared to previous releases of the code. The overall savings this provides on the whole calculation will be documented in subsequent publications.

## 5 Topological Analysis of Electron Density and Its Laplacian for Lanthanides and Actinides

Chemical bonding of *f* electrons is a complex and fascinating phenomenon, yet to be fully rationalized, with both fundamental and technological implications. Strong relativistic effects, strong electron correlation, and weak crystal fields contribute to the identification of a broad active valence manifold constituted by the 5*f*, 6*p*, 6*d*, and 7*s* orbital shells in actinide complexes, whose degrees of participation in the formation of chemical bonds varies as a function of several factors and along the actinide series.^165−168^ In particular, the 5*f* electrons are known to participate in bonding from thorium up to plutonium and then to abruptly become less involved from americium on.^169,170^ An intriguing, much investigated, but still elusive, aspect of actinide chemistry is the occurrence and degree of covalency of 5*f* electrons in the chemical bonding.^165,171−173^ A variety of techniques can be used to characterize chemical bonding in lanthanide and actinide compounds, both experimentally and theoretically.^174−176^

A general, formally rigorous, technique that allows for a consistent and quantitative description of multiple aspects of chemical bonding is represented by the quantum theory of atoms in molecules and crystals (QTAIMAC), where the description of chemical bonding is based on a topological analysis of the electron density ρ(**r**) of eq 35a.^177,178^ The bonding features are analyzed by identifying critical points **r** = **r**_CP_ of the density, which are defined as those points where the gradient of the density is vanishing ***∇***ρ(**r**)|_**r**=**r**_CP__ = **0**. Critical points may also be classified according to the eigenvalues of the Hessian matrix of ρ(**r**) evaluated at **r** = **r**_CP_. Thus, information on chemical bonding in the system requires first and second derivatives of ρ with respect to the electron coordinate **r**. Further information on charge concentration in the system may be obtained through a topological analysis of the Laplacian of the electron density (i.e., the trace of the Hessian matrix), which then requires third and fourth derivatives of ρ(**r**) with respect to **r**.^179^

The topological analysis of the electron density ρ(**r**) and of its Laplacian ∇^2^ρ(**r**) was implemented in the Topond program^178,180,181^ that was merged into Crystal14 and parallelized.^182^ In Crystal23, the strategy for evaluating ρ(**r**) and its first to fourth derivatives has been extended to *f*- and *g*-type orbitals (previously being usable only for *s*-, *p*-, and *d*-type orbitals). This opens the possibility for a topological analysis of the electron density of lanthanide and actinide containing systems, with *f* electrons in the valence.^183,184^

The previous strategy (general to *s*-, *p*-, and *d*-type RSSH-GTFs) was based on an expansion of the AO overlap distribution in eq 35a into Hermite GTFs, using eqs 55, 56, 58, and 60. This resulted in an highly efficient algorithm, which, however, may not be easily generalized to higher quantum numbers. A new algorithm has therefore been devised (general to *s*-, *p*-, *d*-, *f*-, and *g*-type orbitals) by a direct evaluation of the AO overlap distribution (and its first to fourth derivatives) in the RSSH-GTF basis.^183,184^ This new algorithm, being less efficient than the previous one based on an expansion in Hermite GTFs, is only activated upon runtime if *f*- and *g*-type orbitals are used in the basis set for a particular calculation.

### 5.1 Example Applications

**Figure 13:**
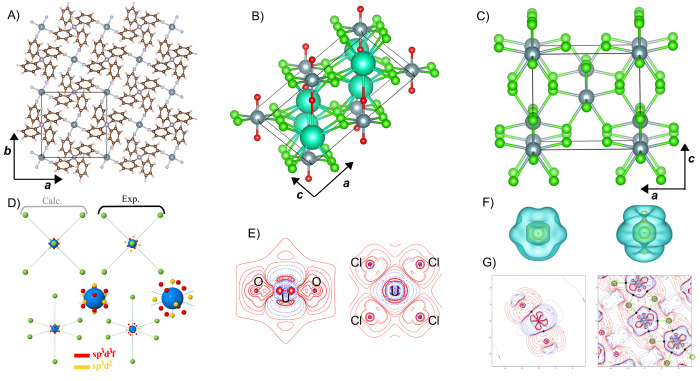
Atomic structure of (A) the tetraphenyl phosphate uranium hexafluoride crystal, [PPh_4_^+^][UF_6_^–^]; (B) the cesium uranyl chloride crystal, Cs_2_UO_2_Cl_4_; and (C) the UCl_4_ crystal. (D) Spatial distribution of the VSCC critical points of the Laplacian of the density ∇^2^ρ(**r**) around the U atom of the [PPh_4_^+^][UF_6_^–^] crystal in the calculations (left) and in the experiments (right). A zoomed-in view in the vicinity of the U atom is also shown. (E) Deformation density, *Δρ*(**r**), contour maps of the cesium uranyl chloride crystal around the U atom in two different planes: (left) through the O–U–O axis and the **b** crystal lattice vector and (right) the equatorial plane of the four Cl atoms. Contour values are ±0.05, 0.15, 0.25, 0.4, 0.7, 1.0, 1.5, 2.0 *e*/Å^–3^. Red and blue lines correspond to positive and negative values, respectively. (F) Distribution of *L*(**r**) around the U atom in a 3D representation where isosurfaces of ±0.4 *e*/Bohr^5^ are shown (yellow for positive, blue for negative) for the frozen molecular fragment UCl_4_ (left) and for the periodic UCl_4_ crystal (right). (G) Deformation density, *Δρ*(**r**), contour maps of the frozen molecule (left) and of the crystal (right) on a plane passing through two U–Cl_fnn_ and two U–Cl_snn_. Bond critical points are marked by small black circles. The atomic structure of the crystal in the selected plane is superimposed in the right panel to help the interpretation of the plots.

We have applied the new extension to *f* electrons of the Topond module to the study of some uranium compounds: the tetraphenyl phosphate uranium hexafluoride crystal, [PPh_4_^+^][UF_6_^–^]; the cesium uranyl chloride crystal, Cs_2_UO_2_Cl_4_; and the UCl_4_ crystal. Graphical representations of their atomic structures are reported in Figure 13.

Crystals of [PPh_4_^+^][UF_6_^–^] belong to the tetragonal *I*4 space group. The UF_6_ molecular fragments in the crystal are distorted with four equatorial fluorine atoms, F_*e*_, and two slightly more elongated apical fluorine atoms, F_*a*_. Thanks to an improved protocol in data collection and reduction, Pinkerton and co-workers were recently able to experimentally reconstruct the charge density of the [PPh_4_^+^][UF_6_^–^] crystal and to perform a QTAIMAC analysis.^185^ We have performed a thorough analysis of its chemical bonding features and compared to the experiments, that we have presented elsewhere.^183^ Here, in Figure 13D, we present a comparison of calculated and experimental valence shell charge concentrations (VSCCs) as they are particularly relevant to the rationalization of chemical bonding. VSCCs are defined as critical points of the Laplacian of type (3,+3) . A total of 14 VSCCs were experimentally reported around the U atom: (i) eight critical points arranged at the vertices of a cube with the edges slightly tilted off the U–F axes (red spheres in the figure), (ii) four critical points forming a square in the equatorial plane, with vertices slightly tilted off the bisector of the F_*e*_–Û–F_*e*_ angle (yellow spheres in the figure), and (iii) two critical points along the U–F_*a*_ axes (yellow spheres in the figure). Experimentally, all 14 VSCCs are at a distance of about 0.38 Å from U. Present calculations are able to confirm the whole set of 14 critical points found in the experiments. The predicted radial distance of the (3,+3) critical points of the Laplacian is of 0.30 Å and coincides with the minimum of the VSCC of the principal quantum number 6. Furthermore, according to present calculations, the 14 critical points can be grouped into two independent sets with slightly different properties: eight critical points arranged at the vertices of a cube (red spheres in the figure) and six critical points arranged at the vertices of an octahedron (yellow spheres in the figure). The only difference with respect to the experiment consists in the red cube and yellow octahedron not to be tilted off the U–F bonds, which, however, seems consistent with the symmetry of the system. The spatial distribution of the two sets of VSCCs around the U atom can be rationalized in terms of the hybridization of the valence atomic orbitals. It has recently been shown that a *sp*^3^d^2^ hybridization leads to a octahedral 6-fold coordination and a *sp*^3^d^3^f hybridization leads to a cubic 8-fold coordination.^186^

Cesium uranyl chloride, Cs_2_UO_2_Cl_4_, crystallizes in a monoclinic lattice with space group C2/*m*. Each U atom forms two symmetry-equivalent bonds with O atoms (bond length of 1.776 Å in the experimental geometry) as well as four symmetry-equivalent bonds with Cl equatorial ligands (bond length of 2.670 Å). Each Cs atom is connected with eight Cl atoms (four symmetry-independent pairs with bond lengths in the range 3.502–3.624 Å) and one O atom (bond length of 3.259 Å). For a better comparison with the experimental electron density,^187,188^ we have performed our quantum-mechanical simulations on the experimental geometry. We have reported our results in ref (184). We present deformation density (DD) maps in Figure 13E. Deformation density (relative to a neutral atomic reference) and *Δρ*(**r**), contour maps of the cesium uranyl chloride crystal around the U atom are shown in two different planes: (left) through the O–U–O axis and the **b** crystal lattice vector and (right) the equatorial plane of the four Cl atoms. The left panel shows the nearly axial symmetry of the U–O interaction. In particular, the DD of present quantum-mechanical calculations corroborates^167,176,189^ the previously suggested “triple bond” nature of the U–O interaction with a *sp* hybridization of the oxygen and the formation of a σ bond along the U–O axis and, supposedly, two π bonds with a maximum of charge deformation at about 0.71 Å off the axis. We have observed large differences between the experimental and computed DD in the equatorial plane of the four Cl atoms. In particular, the expected nearly 4-fold symmetry of this plane seems to be lost in the experimental DD, while it is still largely there in the computed DD of the right panel. The large departure from the expected symmetry was acknowledged in ref (188) and tentatively attributed to the different crystal environment at the second and third nearest neighbor level. While present calculations do show some asymmetry in the equatorial plane, they predict it to be very subtle while preserving the overall symmetric distribution of the density around the U atom in this plane. However, both theory and experiments describe a charge depletion close to U and a charge accumulation close to Cl along the U–Cl bonds, indicative of a higher ionic character of this interaction relative to the U–O one.

The UCl_4_ crystal belongs to the tetragonal *I*4_1_/*amd* space group. All calculations on the periodic structure of the UCl_4_ crystal have been performed on the experimental geometry. Each U atom in the crystal is linked to four first nearest neighbors, Cl_fnn_, at 2.64 Å and to 4 *s* nearest neighbors, Cl_snn_ at 2.88 Å. We present deformation density (DD) maps in Figure 13 G) on a plane passing through two U–Cl_fnn_ and two U–Cl_snn_ bonds in the crystal. Deformation densities (relative to a neutral atomic reference) are reported both for the isolated frozen molecule (left) and for the crystal (right). Isovalues of the contours are given in the figures. The DD of the frozen molecular fragment clearly shows the charge accumulation around the Cl atoms (particularly so along the direction of the bond with the U atom and toward it) and charge depletion around the U atom. Small charge depletion areas are visible beyond the two Cl atoms. Interestingly, while overall the U atom is characterized by a charge depletion basin all around it, a 4-fold charge accumulation pattern is visible in the vicinity of the U atom in those directions of space away from the bonds and somehow between ligands (not just the two Cl_fnn_ lying on the plane of the figure but also the other two above and below the plane). When passing from the left panel to the right one (i.e., passing from the frozen molecular fragment to the actual periodic crystal), the DD changes significantly, thus clearly showing how crystal field effects are extremely relevant to the characterization of chemical bonding in this system. In the crystal, each Cl atom is now involved in two bonds with U atoms, and thus, its charge distribution is affected by the formation of a further bond. Perhaps the most interesting feature of the DD of the crystal relative to that of the frozen molecule is the redistribution of the electron density around the U atom. The 4-fold pattern of charge accumulation visible in the DD of the molecule is significantly reduced, while a large 2-fold buildup of electron density is observed along a direction that bisect both the Cl_fnn_–Û–Cl_fnn_ and the Cl_snn_–Û–Cl_snn_ angles. These effects are also visible by analyzing the deviation from the spherical distribution of the Laplacian *L*(**r**) in the vicinity of U. Figure 13F shows 3D isosurfaces of *L*(**r**), with isovalues of ±0.4 *e*/Bohr^5^ (yellow for positive, blue for negative) for two structures: the frozen molecular fragment (left) and the actual periodic crystal (right).

## 6 Basis Sets

In this section, we discuss recent developments related to the use and optimization of basis sets of atom-centered atomic orbitals for condensed matter simulations. In Crystal23, the LCAO approach has been extended to the use of *g*-type basis functions (previously limited to up to *f*-type basis functions).^159^ Some algorithms that were limited to work with angular functions only up to *d*-type (such as the evaluation of the electron density, its derivatives and its topological analysis) have now been extended to *f*- and *g*-type functions.^183,184^

### 6.1 New Consistent Basis Sets for Solids

#### 6.1.1 POB Sets

Bredow and co-workers have been optimizing consistent local basis sets for solids since 2013, which initially led to the very popular (certainly among Crystal users) pob-DZVP and pob-TZVP sets.^190^ Here, we briefly review the developments made in this respect since the release of Crystal17 and now available in Crystal23.

It was observed that the original pob basis sets suffer from the basis set superposition error (BSSE). In order to reduce this effect, the basis optimization process has been upgraded by taking into account the counterpoise energy of hydride dimers as an additional parameter. Based on the experience with the original pob-TZVP basis set, other optimization parameters were also modified, such as the threshold for the smallest value of orbital exponents, to achieve higher accuracy and better overall performance. This scheme has led to revised consistent all-electron basis sets for elements in the range H–Br: pob-DZVP-rev2 and pob-TZVP-rev2.^191^ The overall performance, portability, and SCF stability of the resulting rev2 basis sets are significantly improved compared to the original pob basis sets. The pob-TZVP-rev2 basis sets have been further extended to elements of the sixth period in the range Cs–Po, which are based on the fully relativistic effective core potentials (ECPs) of the Stuttgart/Cologne group and on the def2-TZVP valence basis of the Ahlrichs group (see ref (192) and references therein). Finally, the same strategy has been applied to optimize consistent pob-TZVP-rev2 basis sets for the elements of the fifth period as well, in the range Rb–I.^193^

**Figure 14:**
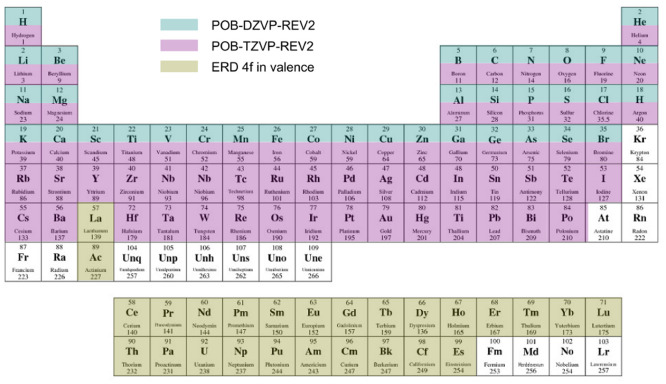
Schematic representation of what new consistent local basis sets are available in Crystal23 for what elements of the periodic table.

Figure 14 shows for what elements of the periodic table such rev2 basis sets have been optimized. Both pob-DZVP-rev2 and pob-TZVP-rev2 basis sets have been coded into internal libraries of Crystal23 and thus can be activated just by use of the corresponding keywords POB-DZVP-REV2 and POB-TZVP-REV2.

#### 6.1.2 ERD Sets for Lanthanides and Actinides

The key role of 4*f* electrons of lanthanides and 5*f* electrons of actinides on the electronic and magnetic properties of their complexes and compounds calls for the development of basis sets where these are left out of the ECPs. A consistent series of basis sets with the 4*f* or 5*f* orbitals in the valence, to go with small-core ECPs, has been devised and optimized for the whole lanthanide series and for the actinide series up to Es, specifically for solid state applications.^159,194^

The adopted procedure is briefly sketched here. The format of the optimized basis sets is ECP28MWB-(11*s*11*p*7*d*8*f*2*g*)/[4*s*4*p*2*d*3*f*2*g*], where scalar relativistic effects are treated with the Wood–Boring Hamiltonian,^195,196^ and the valence part is described by four *sp* shells, two *d* shells, three *f* shells, and two *g* shells. The process starts by optimizing the coefficients and exponents for *s*, *p*, *d*, and *f* shells for the isolated atom. In particular, the *f* shells are optimized with a partial occupation corresponding to the 3+ cation. The most diffuse exponent of the *sp*, *d*, and *f* shells is then reoptimized in the X_2_O_3_ sesquioxide solid (with *X* being any lanthanide or actinide element considered) and two *g* shells are added, which represent the first polarization of the occupied *f* orbitals. Figure 14 shows for what lanthanides and actinides such basis sets have been optimized.

### 6.2 Basis Set Internal Optimizer

We have implemented a novel algorithm that allows for automatic basis set optimization directly from within the code.^197^ Following the proposal of VandeVondele and Hutter,^198^ the optimization relies on the minimization of a suitable functional,
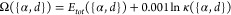
65where α and *d* are exponents and coefficients defining the basis set, as in eq 55, and κ({α,*d*}) is the ratio between the largest and the smallest eigenvalue of the overlap matrix at the center of the Brillouin zone (Γ–point). Its purpose is to prevent the onset of linear dependency within the basis set, which could lead to numerical inaccuracies and, ultimately, to catastrophic behavior.

The minimization is performed according to an algorithm analogue to DIIS (direct inversion of the iterative subspace),^199,200^ which we called BDIIS (basis-set DIIS). At a given iteration *i* in the optimization procedure, we define the error vectors *e*_*i*_^α^ and *e*_*i*_^*d*^ as the gradients in Ω with respect to exponents and contraction coefficients to be evaluated numerically,
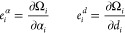
66where Ω_*i*_ is evaluated from eq 65 by using the current α_*i*_ and *d*_*i*_. The DIIS error matrix elements *ij* are built from the scalar products between the errors in iteration *i* and *j*,
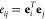
67By imposing the constraint ∑_*i* = 1_^*n*^*c*_*i*_ = 1, we can obtain at each iterative step *n* the linear combination coefficients of the BDIIS method by solving the linear equation system
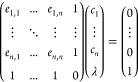
68where λ is a Lagrange multiplier. Such coefficients are then used to obtain the new estimate as a linear combination of the trial vectors obtained in previous iterations
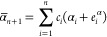
69
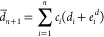
70For exponents, the numerical derivatives of eq 66 are evaluated using a three-point formula and a displacement that is 1% of the initial exponent value. A linear search is finally performed, at each iteration, along the direction defined by the BDIIS algorithm, so to search for the optimal step length.^197^

As an illustrative example, we report, in Table 4, a comparison among the valence exponents of the original def2-TZVP, the recent pob-rev2 basis set, and our TZVP basis specifically optimized for diamond or graphene with the PBE functional. For brevity, we refer to the latter two basis sets as dcm[*C*_*diam*_]-TZVP and dcm[*C*_*graph*_]-TZVP, respectively. Core orbitals were not optimized.

**Table 4:** Top: Uncontracted Gaussian Exponents for Different Carbon TZVP Basis Sets.^a^ Bottom: Total Energies at the DFT/PBE Level for Diamond and Graphene as Computed with Different Triple-ζ Basis Sets^b^

	def2^201^	pob-rev2^191^	dcm[*C*_*diam*_]	dcm[*C*_*graph*_]^197^
s	0.5770	0.4941	2.7288	1.0961
	0.2297	0.1644	0.7083	0.5911
	0.0952	–	0.2754	0.2374
p	0.2889	0.5662	0.6187	0.3387
	0.1006	0.1973	0.2713	0.1594
d	1.0970	0.5792	2.0114	1.2502
	0.3180	–	0.6265	0.7194
f	0.7610	–	1.0624	0.7067
				
E_*TOT*_^*diam*^		–76.154752	–76.161457	–
E_*TOT*_^*graph*^		–76.158920	–76.158342	–76.169383

a dcm[*C*_*diam*_]-TZVP and dcm[*C*_*graph*_]-TZVP refer to our basis set optimized by BDIIS with the PBE functional in diamond and graphene, respectively.

b Energies in Hartree.

We observe how the optimized basis is different from the molecular one, with an overall contraction of exponents. Then, it can be seen how the different chemistries and atomic densities affect the optimal basis set exponents. The outermost *p*-type function is the most different in diamond and graphene. The more diffuse *p* function is responsible for the failed convergence when using the graphene dcm[*C*_*graph*_]-TZVP basis set in diamond (bottom of Table 4). Also *d*- and *f-*type functions have a somewhat different spread in the two systems, reflecting the role of quadrupole and octupole interactions.

In the bottom of Table 4, we report the total energies obtained at the DFT/PBE level: in addition to dcm-TZVP and pob-TZVP bases, the dcm[*C*_*diam*_]-TZVP basis was also tested in graphene and the dcm[*C*_*graph*_] -TZVP in diamond. We see that the properly optimized basis sets gain a considerable margin in absolute value with respect to the general-purpose one. On the other hand, swapping the two dcm-TZVP bases led to energies similar to that of pob-TZVP[G], but the more diffuse dcm[*C*_*graph*_]-TZVP led to a failed SCF convergence.

Another example of a powerful application of the basis set optimization method can be found in ref (202). In that work, we show—in the case of bilayer graphene—how a quadruple-ζ basis set can be obtained that matches in full detail the band structure of a plane wave basis with a high cutoff. It is remarkable how such accuracy in the bands can be obtained only by minimizing the functional of eq 65.

As a general strategy, we suggest starting from a suitable molecular basis set, contracting the most diffuse outer exponent so as to let a basic SCF go through, and then letting the optimizer work out the optimal value.

### 6.3 Perturbative Treatment of Diffuse Basis Functions

We have developed a novel approach that allows converging the SCF in a given basis set and evaluating perturbatively *a posteriori* the effect of enlarging the basis set,^203,204^ e.g., adding somewhat more diffuse functions that would be troublesome. The idea of a dual basis set treatment dates back to Wolinski and Pulay,^205^ mainly in connection with the MP1 singles term that arises when adopting the approach in the framework of Møeller–Plesset theory. Important work in this direction was done by Martin Head-Gordon and co-workers,^206^ with a different method, that allows only for energy correction. Our treatment, conversely, allows also for wave function and eigenvalues corrections.

Let us introduce a “small” basis (S) and a “large” basis (L), assuming that S is a subset of L. We first solve the SCF in the S basis ^*S*^**F_k_**^*S*^**C_k_** = ^*S*^**S_k_**^*S*^**C_k_**^*S*^Ek and then define the projector from the S to the L basis for each **k** point as
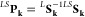
71

Here,  is the overlap matrix between the L and S basis sets. Next, we separate the space spanned by the basis functions L into two subspaces, one of which is spanned by the S basis (∥) while the other (⊥) is orthogonal to it. For that purpose, it is useful to define the matrix _SL_**P_k_** ≡ ^*S*^**S**_**k**_^−1^_SL_**S_k_** and then introduce the matrix  which represents the projection from the L space to the S space and back, together with its complementary matrix 
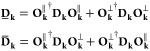
72By separating the core and bielectronic parts of the Fock matrix **F**_**k**_ = **h**_**k**_ + **B**_**k**_, we can now define the perturbation operator **Ω**_**k**_^(1)^ and the first orders in the perturbation series for **F**_**k**_,
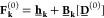
73
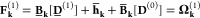
74
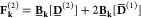
75

We skip the quite lengthy derivations—to be found in the reference papers—to only present here the perturbation energy corrections,
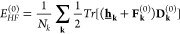
76
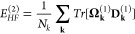
77
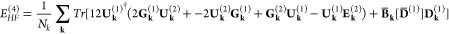
78where the proper occupied or virtual blocks of the involved matrices have to be considered (see refs (203 and 204)). First- and third-order energies are null by construction. In eq 78, we have introduced the **G**_**k**_^(*n*)^ and **U**_**k**_^(*n*)^ matrices. The former is simply the Fock matrix of order *n* in the basis of unperturbed crystalline orbitals: **G**_**k**_^(*n*)^ = **C**_**k**_^(0)†^**F**_**k**_^(*n*)^**C**_**k**_^(0)^. The matrix **U**_**k**_^(*n*)^ yields the perturbed coefficients: **C**_**k**_^(*n*)^ = **C**_**k**_^(0)^**U**_**k**_^(*n*)^. Once the **U**_**k**_^(*n*)^ matrices are available, the density and wave function of order *n* can be simply evaluated.

**Figure 15:**
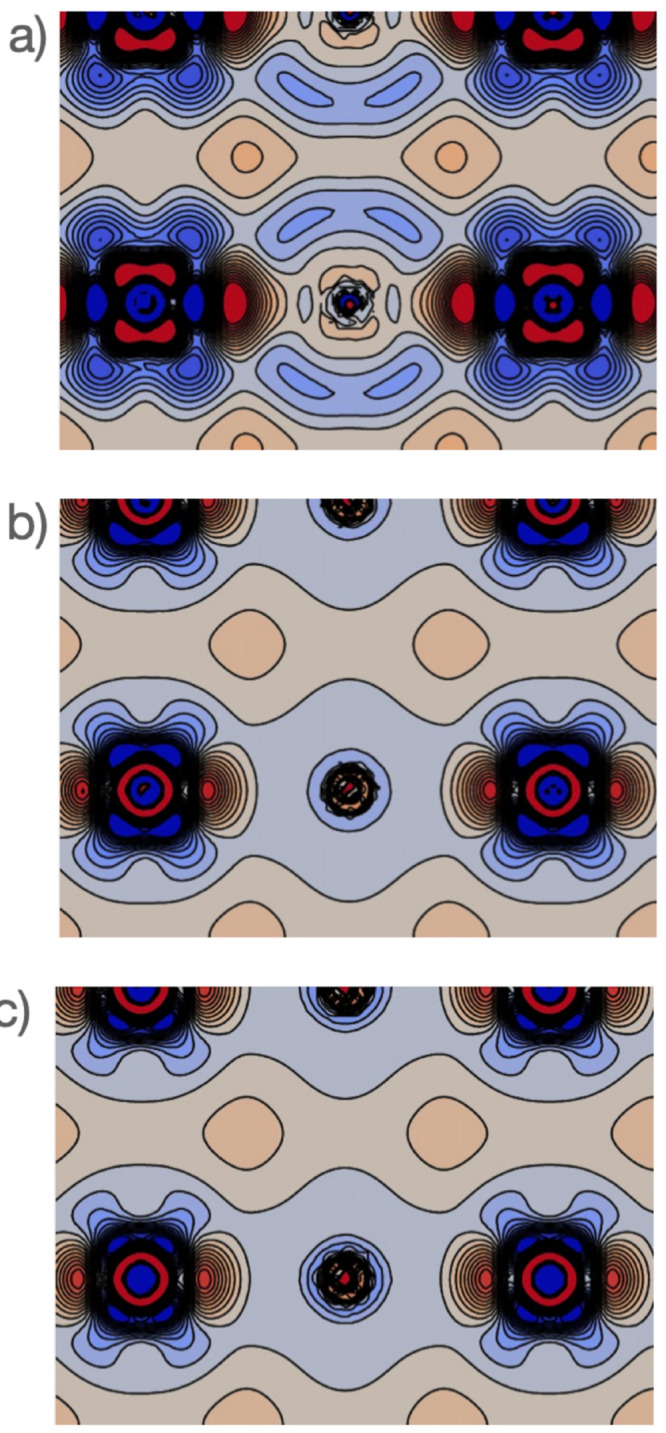
Dual basis set perturbative approach, NaCl solid. Difference charge density maps are shown in (a, b, c) for zeroth, first, and second perturbative orders, respectively. The difference is computed with respect to the charge density of the reference large basis set. Details as in Table 5. Isoline spacing is set to 10 μBohr.

In Table 5 and in Figure 15, we report some demonstrative results for the NaCl bulk crystal, at the PBE level, using two basis sets. The small basis set used is in both cases optimized on the NaCl crystal with the BDIIS method above described, obtaining the so-called dcm-SVP and dcm-TZVP basis sets.^197^ The large basis is obtained by adding to such basis *d* and *f* diffuse polarization shells on the Cl atom (only *d* functions for the smaller basis). From Table 5, we see that more than 80% of the difference in energy between the two basis sets in the SVP case is recovered at the second perturbative order and more than 95% at the fourth order. For the TZVP case, the starting difference is smaller, slightly more than half a kJ/mol. Our perturbative approach recovers here 85% and 95% of that error at the second and fourth order in energy, respectively, reducing the absolute error to 0.02 kJ/mol.

**Table 5:** Dual Basis Set Perturbative Approach Applied to Solid NaCl and Different Basis Sets^a^

	SVP basis		TZVP basis	
	error (kJ/mol)	% error	error (kJ/mol)	% error
Δ*E*^(0)^	65.9822	100.00	0.5149	100.00
Δ*E*^(2)^	12.0694	18.29	0.0749	14.54
Δ*E*^(4)^	3.1721	4.81	0.0253	4.92

a Errors in total energy (absolute and relative) for different perturbation orders. Details in text.

As we have outlined above, our approach allows us to correct not only the total energy but also the wave function and the hence density. In Figure 15, we show how for the TZVP basis the differences in the density are substantially recovered at the first order in the wave function, while the second order effect appears minor. We already had a chance to point out how the latter contribution mostly affects the virtual manifold.^203^

## 7 Anharmonicity of Lattice Vibrations

Atomic vibrations are at the core of a variety of properties of finite molecular systems and extended solids. In particular, thermal properties of materials (such as specific heat, entropy, thermal expansion, thermoelasticity, lattice thermal conductivity, etc.) are connected to the lattice dynamics of the system.^207^ Statistical thermodynamics provides the link between the microscopic atomistic description of the nuclear dynamics (i.e., the quantum-mechanical vibrational states) and macroscopic thermal properties of matter.^208^ The energies of vibrational states of molecules can be effectively probed with vibrational spectroscopies such as infrared and Raman. The same techniques are used to probe those lattice vibrations of solids where atoms of different lattice cells move in phase with each other (i.e., phonons at the Γ point of the Brillouin zone).^209^ Inelastic neutron scattering can be used to probe also out-of-phase vibrations (i.e., the so-called phonon dispersion).^210^

In the context of quantum-mechanical simulations of materials, the standard way in which the lattice dynamics of the system is described is by means of the harmonic approximation (HA) of the Born–Oppenheimer potential energy surface (PES).^211^ The HA assumes a quadratic form of the Taylor’s expansion of the PES in terms of atomic displacements from the equilibrium configuration and implies a description of the lattice dynamics in terms of a set of independent quantum harmonic oscillators. Despite its simplicity, the HA has experienced great success in the description of lattice vibrations of many classes of materials,^212−217^ in particular, those without light elements (mainly hydrogen)^218,219^ and without strongly anharmonic phonon modes, such as ferroelectric ABO_3_ perovskites, for instance.^220−223^

At the same time, the limitations of the HA are well known and, in a solid state context, can be grouped into two classes: (i) the constant-volume nature of all computed thermal properties of materials and (ii) neglected high-order terms of the PES, which result in the independence of phonon modes. The first class of limitations is such that the HA is unable to describe the thermal lattice expansion of the system, as well as its thermoelasticity (i.e., thermal dependence of the mechanical response). Furthermore, at the harmonic level, there is no distinction between constant-volume and constant-pressure thermodynamic functions. These limitations can be effectively overcome by using the so-called quasi-harmonic approximation (QHA), which requires the evaluation of harmonic phonon frequencies as a function of lattice cell volume.^224,225^ The Crystal program (since Crystal17) already has a fully automated module for the calculation of quasi-harmonic thermal properties of materials.^226−237^ The second type of limitation is due to neglected higher-than-second order terms in the expansion of the PES, so that the intrinsic anharmonicity of the phonon modes as well as phonon–phonon couplings, and their effects on vibrational states (such as Darling–Dennison and Fermi resonances and phonon combination bands), are in turn neglected, which results in the approximated description of spectroscopic features and thermodynamic properties.^238,239^ As a further consequence of the lack of cubic terms of the PES within the HA, phonon lifetimes τ would be infinite as well as the lattice thermal conductivity of the material.

In Crystal23, we now have implemented algorithms for the evaluation of high-order terms of the PES^240^ and for the vibrational self-consistent field (VSCF) and vibrational configuration interaction (VCI) calculation of anharmonic vibrational states.^241^

In the following, we will discuss some formal aspects of vibrational states of molecules and solids, where, in the case of solids, we restrict our attention to Γ-point vibration modes. However, let us note that, by working in terms of a supercell of the primitive one, vibration modes of solids proper of different **k**-points can be folded back to the Γ-point. The starting point of our anharmonic vibrational description is represented by the harmonic approximation according to which the nuclear dynamics of the system is described in terms of a set of *M* independent quantum harmonic oscillators, whose corresponding normal coordinates are *Q*_1_, *Q*_2_, ···, *Q*_*M*_ ≡**Q**.

### 7.1 High-Order Terms of PES

The numerical description of high-order terms of the PES represents the most delicate and computationally expensive step in the anharmonic treatment of vibrational states of materials. In our strategy, the PES is truncated to quartic order and contains one-, two-, and three-mode interatomic force constants. Four different numerical approaches have been implemented, all based on a grid representation of the PES in the basis of the normal coordinates, that require different ingredients (energy and/or forces) to be evaluated at each point (i.e., nuclear configuration) of the grid. Different algorithms have been explored to compute the high-order energy derivatives: energy fitting and finite differences. The numerical stabilities and relative computational efficiencies of the various schemes have been discussed in ref (240).

Within the Born–Oppenheimer approximation, vibrational states are determined by solving the nuclear Schrödinger equation, which, in terms of normal coordinates, reads
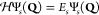
79where Ψ_*s*_(**Q**) is the vibrational wave function of the *s*th vibrational state and *E*_*s*_ the corresponding energy. By setting the rotational angular momentum to zero and by neglecting rotational coupling effects, the Hamiltonian operator in eq 79 can be written as
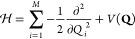
80where *V*(**Q**) is the usual Born–Oppenheimer potential energy surface (PES) in the basis of mass-weighted normal coordinates. As discussed above, the description of the potential term in the Hamiltonian is a computationally challenging task. Here, we expand the PES in a Taylor’s series centered at the equilibrium nuclear configuration as follows:
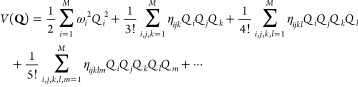
81where ω_*i*_ is the harmonic frequency of the *i*th vibration normal mode and where η_*ijk*_, η_*ijkl*_, and η_*ijklm*_ are cubic, quartic, and fifth-order force constants, respectively,
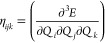
82
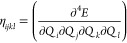
83
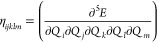
84The inclusion of anharmonic (i.e., higher than quadratic) terms in the potential (eq 81) therefore implies the evaluation of high-order energy derivatives with respect to atomic displacements. These high-order energy derivatives are computed numerically, which makes the description of the PES a computationally demanding task. For this reason, it proves crucial to devise (i) effective strategies to truncate the expansion of the PES in eq 81 so as to include only those terms contributing significantly to the description of the vibrational states of the system and (ii) efficient algorithms for the numerical evaluation of the high-order energy derivatives in eqs 82–84.

#### 7.1.1 Truncation of PES

We include only terms up to fourth order in the PES (namely, we use a 4T representation of the potential). Within a 4T representation, the PES can be further truncated by considering only those force constants involving a maximum of *n* distinct modes (namely, a *n*M representation of the potential). By combining the two truncation strategies introduced above, a 1M4T representation of the PES would require the evaluation of these force constants,
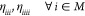
85This representation of the PES neglects two-mode couplings and almost always results in a wrong description of the vibrational states. A popular representation of the potential is the 2M4T one, which includes all two-mode coupling force constants,
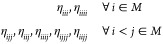
86Analogously, the 3M4T representation of the PES includes the following terms:
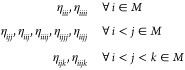
87Here, we work in terms of 2M4T and 3M4T representations of the PES as given in eqs 86 and 87, respectively.

#### 7.1.2 Numerical Evaluation of High-Order Force Constants

**Figure 16:**
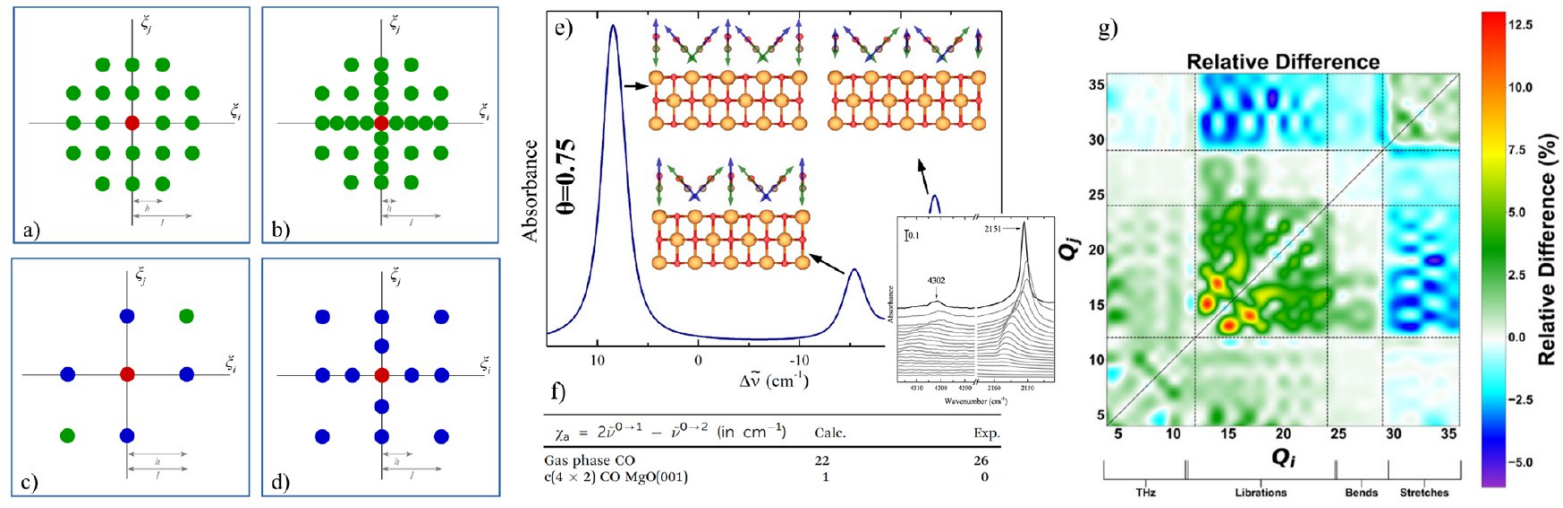
(a–d) 2D grid of points defining the nuclear configurations that need to be considered in the evaluation of the adiabatic PES in its 2M4T representation for the four different numerical schemes implemented. Different colors correspond to different quantities computed for each nuclear configuration: only energy (green), energy and forces (blue), and energy, forces, and Hessian (red). (e) Simulated infrared spectrum of the low-temperature Co Ad-layer on MgO (001) from quantum-mechanical calculations and graphical representation of the normal modes of vibration associated with the three intense peaks (arrows of the same color correspond to in-phase atomic motions). *Δν̃* is the frequency shift with respect to the CO stretching in gas phase. The bottom right inset shows experimental FTIR spectra of CO molecules adsorbed on (001) MgO surfaces, recorded at 60 K, as a function of surface coverage (spectra are vertically offset for clarity) in the spectral region of the fundamental transition for the in-phase stretching motion of all CO molecules (right panel) and of the corresponding first overtone (left panel). (f) Anharmonic coefficient χ_*a*_ = 2ν̃^0→1^ – ν̃^0→2^ (in cm^–1^) of the stretching mode of CO in gas phase and in the low-temperature ordered *c*(4 × 2) monolayer adsorbed on MgO (001) surfaces. (g) VSCF two-quanta excited-state pair coupling space of ice XI. For each pair (*Q*_*i*_,*Q*_*j*_) of normal modes, *Δω*_*ij*_^PD^ is reported, as defined in eq 98.

We have developed and implemented four different numerical approaches to compute those terms of the PES required to get a 2M4T representation, which we are going to discuss into detail below. Different approaches are characterized by a different numerical stability, accuracy, and computational cost. In order to get two-mode terms, for each pair of modes (*Q*_*i*_,*Q*_*j*_), a grid of points is needed where the energy (and forces, for some approaches) are computed. The shape of this grid is illustrated in panels a–d) of Figure 16 for the four different schemes. The first two schemes only require the evaluation of the energy at each displaced nuclear configuration, while the last two combine information from the energy and forces.

Formal details on the four schemes are given in ref (240). We recommend using scheme c, as it offers an optimal balance between cost and accuracy. Therefore, here we just discuss scheme c.

By computing the analytical gradients at some configurations (only those where atoms are displaced along one normal coordinate at a time), an effective finite difference scheme has been devised,^242^ which is called EGH from the different ingredients it requires: energy, gradients, and Hessian. Figure 16c shows the points needed for each pair of modes (*Q*_*i*_,*Q*_*j*_), where some nuclear configurations only require the energy to be evaluated while others require energy and gradients. The Hessian matrix is computed just at the equilibrium nuclear configuration to get the harmonic normal modes and frequencies. For each pair of modes, all the terms of the 2M4T representation of the PES in eq 86 can be obtained from the following finite difference relations:
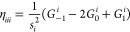
88
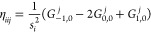
89
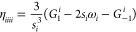
90
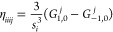
91
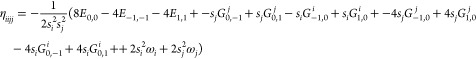
92where  and  are the adaptive steps among the points of the grid along the *Q*_*i*_ and *Q*_*j*_ normal coordinates (see ref (240) for more details on how the step *h* is defined) and where *E*_*a*,*b*_ is the energy computed at a nuclear configuration displaced by *a s*_*i*_*Q*_*i*_ + *b s*_*j*_*Q*_*j*_ from the equilibrium one. For those terms of the PES involving only one mode, a more compact notation is used where *E*_*a*_ is the energy of a nuclear configuration displaced by *a s*_*i*_*Q*_*i*_ from the equilibrium one. *G*_*a,b*_^*i*^ is the gradient with respect to *Q*_*i*_ computed at a nuclear configuration displaced by *a s*_*i*_*Q*_*i*_ + *b s*_*j*_*Q*_*j*_ from the equilibrium one (analogously, *G*_*a,b*_^*j*^ is the gradient with respect to *Q*_*j*_ computed at the same nuclear configuration). For those terms of the PES involving only one mode, a more compact notation is used where G_*a*_^*i*^ is the gradient with respect to *Q*_*i*_ of a nuclear configuration displaced by *a s*_*i*_*Q*_*i*_ from the equilibrium one.

### 7.2 VSCF and VCI Approaches

Different approaches (of increasing complexity and accuracy) can be used to solve eq 79 numerically. In particular, several methods have been developed to progressively take into account the correlation among vibration modes, through mode–mode couplings, which are formally analogous to the hierarchy of wave function-based methods in electronic structure theory.^243,244^ The vibrational analog of the Hartree–Fock (HF) method is known as vibrational self-consistent field (VSCF) approach: a mean-field scheme where each vibrational degree of freedom interacts with an average potential over the other modes.^245−247^ In analogy to the definition of dynamical electron correlation, the vibrational correlation among modes is defined as the difference between the exact vibrational states and VSCF ones. In electronic structure theory, the HF solution can be used as a starting point to improve the description of the electronic wave function, passing from a single-determinantal to a multideterminantal representation by using either perturbative (MP2, MP4, etc.) or variational (CC, CI, etc.) approaches. In the vibration theory, starting from the reference VSCF state, the analog of the electronic Møller–Plesset perturbation theory is known as vibrational perturbation theory truncated at *n*th order (VPT*n*),^248−250^ the analog of the coupled-cluster family of methods is the vibrational coupled-cluster approach (VCC),^251,252^ and the analog of the configuration-interaction methodology is the vibrational configuration-interaction (VCI), where mode–mode couplings are treated exactly (at least in the full-VCI limit).

In Crystal23, we have implemented the VSCF and VCI methods for both molecules and solids. In particular, to the best of our knowledge, this is the first implementation of VCI for periodic systems.

Vibrational modes are distinguishable so that the *M*-mode wave function of a given vibrational configuration **n** does not need to be antisymmetrized and can be written as a Hartree product of one-mode functions (modals),^240^
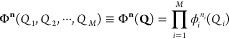
93where **n** = (*n*_1_, *n*_2_, ···, *n*_*i*_, ···, *n*_*M*_) is the vibrational configuration vector of the quantum numbers of the *M* one-mode functions. For each given vibrational configuration **n**, the VSCF method consists in looking for the variationally best form of the corresponding *M* one-mode functions. This is achieved by requiring that the expectation value of the full Hamiltonian is stationary,
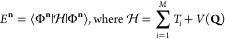
94with *T*_*i*_ = −1/2 (∂^2^/∂ *Q*_*i*_^2^) being the one-mode kinetic energy operator.

In the VCI method, the wave function of each vibrational state *s* is written as a linear combination of *M*-mode wave functions of different vibrational configurations in the form of Hartree products of modals as in eq 93,
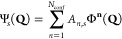
95where the sum runs over *N*_conf_ configurations, each characterized by a vibrational configuration vector **n**. The selections of the *N*_conf_ configurations determine the truncation of the VCI expansion. For each vibrational state *s*, the corresponding VCI wave function and energy are obtained by solving the corresponding Schrödinger equation . The VCI method can be expressed in matrix form as follows: **H A** = **A E**, where **A** is the squared matrix containing, columnwise, the coefficients *A*_*n*,*s*_ of the eigenvectors, **E** is the diagonal matrix of the eigenvalues, and **H** is the VCI Hamiltonian matrix (of size *N*_conf_ × *N*_conf_), whose elements are
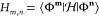
96The VCI method therefore reduces to the construction and diagonalization of the VCI Hamiltonian matrix, from which all vibrational states are simultaneously determined. VSCF solutions can be used to express the modals in the VCI method (according to the so-called VCI@VSCF approach).

#### 7.2.1 Truncation of VCI Expansion

The VCI method relies on the expansion of the wave function of each vibrational state in terms of *N*_conf_ Hartree product functions describing different vibrational configurations, as introduced in eq 95. The number *N*_conf_ of functions used in the VCI expansion is of critical importance with regard to both the accuracy and computational cost of the method. Indeed, the larger *N*_conf_ the better the description of the vibrational state but also the larger the size of the VCI Hamiltonian matrix in eq 96 to be diagonalized. In particular, this latter aspect is the main limiting factor to the application of standard VCI to the study of those systems where more than just a few vibration modes need to be coupled.

Therefore, it is crucial to devise effective schemes to reduce as much as possible the configurational space used in the VCI expansion. We have implemented two such schemes introduced below. The following strategies can be used:

1. The first strategy for the truncation of the VCI expansion consists in including only those vibrational configurations where there are a maximum of *N*_quanta_ excitation quanta involved. Formally, we can express this strategy as follows to say that only those configurations satisfying the next condition are included in the expansion:
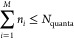
97
2. A second strategy that we use to truncate the VCI expansion consists in setting a maximum number of modes *N*_modes_ that can be simultaneously excited in a given configuration. In other words, only those vibrational configurations where there are a maximum of *N*_modes_ with *n*_*i*_ ≠ 0 are used.

The effect of these two schemes on the truncation of the VCI expansion is documented in ref (241). In regard to *N*_modes_, let us note that, when working in terms of 2M4T or 3M4T representations of the PES, the VCI description converges for *N*_modes_ = 3 and *N*_modes_ = 4, respectively.

### 7.3 Example Applications

We review a couple of recent applications of the methodologies discussed above.

#### 7.3.1 Structure and Dynamics of CO Ad-Layers on MgO Surfaces

The combination of quantum-mechanical simulations and infrared (IR) absorption spectroscopy measurements provides a clear picture for a long-standing puzzle in surface science: the actual structure and vibrational dynamics of the low-temperature-ordered CO monolayer adsorbed on (001) MgO surfaces.^253^ The equilibrium structure of the commensurate (4 × 2) adsorbed phase consists of three CO molecules per primitive cell (surface coverage of 75%) located at two inequivalent sites: one molecule sits upright on top of a Mg site, while two molecules, tilted off the normal to the surface, are symmetrically positioned relative to the upright one with antiparallel projections on the surface. This configuration, long believed to be incompatible with measured polarization infrared spectra, is shown to reproduce all observed spectral features, including a new, unexpected one: the vanishing anharmonicity of CO in-phase stretching modes in the monolayer.

Despite its seemingly simple nature, the physisorption of CO molecules on the clean (001) MgO surface is a complex process whose structural and dynamical features are still far from being fully characterized, particularly so in the low-temperature, high-coverage regime.^254−266^ We have investigated the structure of the low-temperature *c*(4 × 2) phase of the CO monolayer on the (001) MgO surface by means of full structural relaxations within quantum-mechanical calculations based on the density functional theory and found the *H*_0_ configuration to be the most stable one. Based on configurational considerations and on semiempirical potential calculations, the *H*_0_ structure has long been considered to be incompatible with the observed IR fingerprint.^267^ By comparison with previously and newly measured experimental infrared spectra, we have shown instead that all measured spectral features are reproduced by the *H*_0_ configuration.

Furthermore, from the newly recorded infrared spectra, an unexpected spectral feature emerged: the infrared peak corresponding to the first overtone of the in-phase stretching of all CO molecules is recorded at a frequency that is exactly twice as large as that of the corresponding fundamental transition, thus indicating an apparent vanishing anharmonicity in the vibrational potential of the adsorbed CO molecules in the ordered monolayer. The application of the methodologies described above for the calculation of anharmonic vibrational states allowed us to show that the optimized *H*_0_ structural model indeed exhibits this feature when anharmonicity through mode–mode couplings is accounted for.

The computed infrared spectrum in the region of the CO stretching modes is reported in Figure 16e as a function of the frequency shift *Δν̃* with respect to the computed harmonic frequency of gas phase CO. The most intense peak corresponds to the in-phase stretching of all CO molecules in the monolayer. The bottom right inset of Figure 16e shows the unexpected feature of the vibrational spectrum of CO adsorbed on (001) MgO surfaces: the apparent vanishing anharmonicity of the CO stretching vibration in the monolayer. The figure covers the spectral region of the fundamental transition for the in-phase stretching motion of all CO molecules (right) and of the corresponding first overtone (left). The overtone occurs at a frequency, ν̃^0→2^ = 4302 cm^–1^, that is exactly twice as large as that of the fundamental transition, ν̃^0→1^ = 2151 cm^–1^, thus yielding a null anharmonic coefficient χ_*a*_ = 0, as would occur in a perfectly harmonic potential. The CO molecule in the gas phase is instead known to exhibit a significant degree of anharmonicity in its stretching motion,^268^ the fundamental transition occurring at a frequency ν̃^0→1^ = 2143 cm^–1^, and the corresponding first overtone at a frequency ν̃^0→2^ = 4259 cm^–1^. This corresponds to an anharmonic coefficient χ_*a*_ = 2ν̃^0→1^ – ν̃^0→2^ of 26 cm^–1^.

Quantum-mechanical VCI calculations confirm this feature and provide further insight on its origin. First, we studied the anharmonicity of the stretching mode in the CO molecule in the gas phase, which led to an anharmonic coefficient χ_*a*_ = 22 cm^–1^. We then considered the low-temperature ordered monolayer of CO molecules adsorbed on MgO (001). When the sole anharmonicity of the potential of the in-phase stretching mode is considered, an anharmonic coefficient χ_*a*_ = 10 cm^–1^ is obtained, which decreases to χ_*a*_ = 0.7 cm^–1^ when phonon–phonon couplings are explicitly taken into account among all of the six CO stretching modes of the *c*(4 × 2) ordered phase (three of which are infrared active). These findings are summarized in Figure 16f. Therefore, the apparent vanishing anharmonicity of the in-phase CO stretching vibration in the *c*(4 × 2) phase is to be understood as a global effect in the CO monolayer where lateral interactions and couplings among collective vibrations play a key role.

#### 7.3.2 Anharmonicity of O–H Stretching Vibrations in Water Ice

The anharmonicity of O–H stretching vibrations of water ice has been characterized by use of the new periodic implementation of the VSCF and VCI methods.^269^ The low-temperature, proton-ordered phase of water ice (namely, ice-XI) has been investigated. The net effect of a coupled anharmonic treatment of stretching modes is not just a rigid blue-shift of the respective harmonic spectral frequencies but rather a complex change of their relative spectral positions, which cannot be captured by simple scaling strategies based on harmonic calculations. The adopted techniques allow for a hierarchical treatment of anharmonic terms of the nuclear potential, which is key to an effective identification of leading factors. It was shown that an anharmonic independent-mode approximation only describing the “intrinsic anharmonicity” of the O–H stretches is unable to capture the correct physics and that couplings among O–H stretches must be described. By coupling O–H stretches to all other possible modes of ice-XI (THz collective vibrations, molecular librations, bendings), specific types of motion which significantly affect O–H stretching states were identified: in particular, molecular librations were found to affect the stretching states more than molecular bendings.

The first anharmonic treatment of O–H stretching vibrations in ice-XI that was performed was a single-mode one, where the intrinsic anharmonicity of each normal mode is investigated by accounting for cubic and quartic single-mode terms in the PES (i.e., terms η_*iii*_ and η_*iiii*_ in eq 81) and by neglecting mode–mode couplings. In other words, normal modes were still considered as independent but the nonquadraticity of their 1D potential was accounted for. In this limit, the VSCF and VCI methodologies formally coincide. The intrinsic anharmonicity produces an increase of the fundamental vibration frequency of all stretching modes but the lowest frequency one (i.e., the symmetric in-phase stretching on all four water molecules in the cell). While such behavior may seem counterintuitive with respect to what one is used to find in the description of the stretching mode of biatomic molecules (where a simple Morse-like model would predict a lowering of the frequency upon inclusion of high-order terms of the PES), it is common in polyatomic molecules such as water and even more so in molecular crystals. Indeed, while in an isolated water molecule hydrogen atoms move toward dissociation, in ice they move toward the next oxygen atom in their stretching vibration mode.

The strength of the coupling between single pairs of modes was then investigated. As many VSCF and VCI calculations as there are pairs of vibration modes in ice XI, (*Q*_*i*_,*Q*_*j*_) with *i*,*j* = 1,···,*M* were run. Each calculation took into account the third- and fourth-order coupling constants involving just the two respective modes, as in eq 86. In order to quantify the effect of the pair coupling on the vibrational states, a vibrational state where both modes of the pair are simultaneously singly exited (so-called “combination bands” in vibrational spectroscopies) was analyzed. In other words, for each selected pair of modes, the vibrational configuration **n** = (*n*_1_, *n*_2_, ···, *n*_*k*_, ···, *n*_*M*_) with *n*_*k*_ = δ_*ki*_ + δ_*kj*_, where δ is Kronecker’s delta function was considered. From the VSCF approach, we get the energy *E*^**n**^ of this state from eq 94 so that its transition frequency ω_*ij*_^pair^ can be obtained from ℏω_*ij*_^pair^ = *E*^**n**^ – *E*^**0**^, where **0** is the fundamental state. From the VCI approach, the vibrational state *s*′ with the strongest **n** character, i.e., with the largest *A*_*n*,*s*_ coefficient in eq 95, was searched for. The corresponding transition frequency could thus be obtained from ℏω_*ij*_^pair^ = *E*_*s*′_ – *E*_0_.

In order to measure the effect of the anharmonic pair mode coupling over an independent mode anharmonic treatment (as in the intrinsic anharmonic description discussed before), the transition frequency for this two-mode state ω_*ij*_^pair^ was compared with the sum of the singly excited transition frequencies, ω_*i*_^ia^ and ω_*j*_^ia^ of the two individual modes treated at the intrinsic anharmonic level. A percent difference (PD) was calculated relative to the sum of the singly excited frequencies,
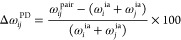
98This quantifies the coupling between a given pair of modes. A large *Δω*_*ij*_ means that the frequency of the doubly excited state cannot be estimated just by summing the two frequencies of the corresponding individual modes. Figure 16g provides a graphical representation of the analysis outlined above. The figure shows (in a color scale) the strength of the pair coupling for each pair of normal modes in ice XI (*Q*_*i*_,*Q*_*j*_) as quantified by eq 98 from the VSCF approach. If two modes do not strongly couple, *Δω*_*PD*_ should be near zero, as this implies the doubly excited state has the same (or nearly the same) energy as the sum of each mode being singly excited independently from the other. Conversely, when the two modes are strongly coupled, there will be a large deviation between the doubly excited state energy and the energies of the two singly excited modes. Inspection of the figure allows some considerations: (i) Weak couplings are observed between stretching and THz vibrations, as well as between bendings and THz vibrations (both cases involve mode types with very different energies). (ii) Interestingly, weak couplings are observed among bending modes (actually, this is the only diagonal block of the matrix showing very small values). (iii) All other diagonal blocks of the matrix show relatively large values, which implies a relatively strong coupling among stretching modes, among librational modes and also among THz modes). (iv) Among the stretching diagonal block, mode 29 (i.e., the symmetric in-phase stretching on all four water molecules) once again behaves differently from other modes yielding negative *Δω*_*ij*_ with all other stretching modes at variance with the positive *Δω*_*ij*_ of all other stretching mode pairs. (v) Some off-diagonal blocks also show large values, indicative of relatively strong couplings between modes of different spectral subsets (this is the case of the stretching-bending block and even more so of the libration-stretching, libration-bending, and libration-THz, which highlights the key role played by the librational motions in the anharmonic behavior of ice, with librations able to couple with all other vibrations).

The effect of mode–mode couplings among modes of different spectral type on the computed anharmonic stretching frequencies has also been investigated and discussed in ref (269).

## 8 MPI+OpenMP Hybrid Parallelism for DFT Energy and Forces

Exploitation of parallelism is crucial for modern *ab initio* electronic codes, and for many years Crystal has been exemplary, as discussed in ref (270). The strategy adopted within the SCF calculation is briefly outlined below and discussed into detail elsewhere:^270,271^

- The strategy is purely based upon message passing between processes.
- The elements of the Kohn–Sham matrix may all be constructed independently, thus a task farming methodology is used. This results in excellent parallelism for large systems, load balancing being the only obstacle to perfect performance. The calculation of the forces is performed similarly.
- For the diagonalization and related linear algebra, Crystal has two versions: PCrystal and MPPCrystal. The former employs a replicated data strategy and can only exploit parallelism over **k** points, while the latter uses a distributed memory strategy and uses ScaLAPACK for the parallel linear algebra.^272^ In this section, we focus solely on MPPCrystal, although in fact many of the improvements discussed also apply to PCrystal.

This scheme works well and has been used to solve a number of challenging problems.^273−275^ However, as discussed in ref (270), it does have one major drawback: In the construction of the Kohn–Sham matrix, a large number of arrays associated with the screening of the integrals are replicated, as are the real space representations of the Kohn–Sham, overlap, and density matrices. Although these are all stored in sparse format, and thus scale linearly with the size of the system, for systems with a large number of basis functions running on large numbers of processes, they can become the most memory consuming objects. This is because on large numbers of processes the *O*(*N*^2^) objects, namely, the reciprocal space representation of the Kohn–Sham and overlap matrices and the eigenvectors, have been “scaled away” due to being distributed over the many cores, while each process has a full copy of the replicated objects.

Reduction in memory use is becoming increasingly important on modern high-performance computing (HPC) clusters. While the compute power is increasing through increasingly large numbers of cores (often in increasingly fat nodes supported by increasingly powerful accelerators), the amount of memory per core is decreasing.^276^ Thus, to access the huge amount of compute power available on modern HPC systems, memory management is of prime importance. After all, if your calculation is inefficient, you just have to wait a bit longer for the result, but if it uses too much memory you cannot do the calculation at all.

As such, to address this problem, a second level of parallelism has been added to the whole of the SCF and forces code in Crystal23 through use of threads implemented via OpenMP. Thus, by instantiating the large replicated objects once per process, they may be held in shared memory and accessed by multiple threads, thus effectively lowering the memory usage. For example, on a single node, use of four processes and four threads instead of 16 processes will roughly quarter the memory used by the replicated objects. This methodology works well with the “fat” nodes now available; while here we only examine the use of a very small number of threads, in principle, many more could be used with a consequent further lowering in memory.

A very brief description of the implementation of the two main sections of the code are dealt with as follows:

- For building the Kohn–Sham matrix, a similar strategy to before is followed; the elements are independent so parallelism whether it be via threads or processes is straightforward. However, it is worth noting that load balancing between the threads within a process is trivial to implement, and thus, some of the residual load balancing issues are addressed;
- For the linear algebra, we assume that the libraries called to perform it, such as ScaLAPACK, are already multithreaded, as is the case nowadays.^277,278^

**Figure 17:**
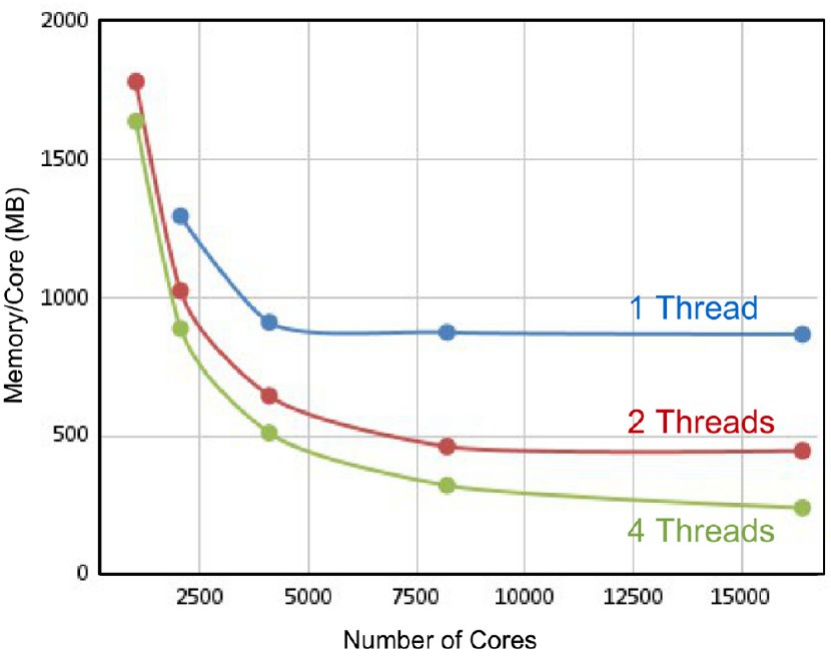
Memory usage per core of MPPCrystal for the MCM-41 X16 case as a function of the number cores and threads. All calculations were run on the Archer2 supercomputer.

Figure 17 shows the memory usage per core for MPPCrystal for a large DFT test case, a 16 way supercell (X16) of the mesoporous amorphous silica, MCM-41. We have used smaller supercells of this example to examine the performance and memory usage of MPPCrystal in earlier studies, and thus, it is well understood.^270^ The X16 supercell contains 9264 atoms and uses 124,640 basis functions and the PBE functional and a single **k** point. The calculations were run on Archer2,^279^ a large modern cluster situated at the Edinburgh Parallel Computing Centre (EPCC) and based upon AMD processors with 2GByte per core and 128 cores per node.^280^ The graph compares running on a given total number of cores with differing numbers of threads. Thus, a run on 8192 cores with two threads consists of 4096 MPI processes, each dual threaded.

It can be seen that at low core counts, less than roughly 2000 cores, the memory usage rapidly decreases with increasing numbers of cores. This is the region where the large *O*(*N*^2^) reciprocal space objects dominate. Distributed over 1024 processes, a single order 124,640 real matrix requires around 116 Mbytes, and as we require several of such sized objects for the calculation, their contribution is easily visible on the scale of the graph. However, by the time we reach 8192 processes, the contribution due to the distributed objects is much smaller, only 15 MBytes per matrix, and so they have all but disappeared leaving only the replicated objects. Instead it can be seen that the different numbers of threads asymptote to different plateaus, with the four thread limit being roughly one-quarter of that for one thread. This is exactly in line with the argument above; the use of threads to share the replicated objects among a small number of cores has made a dramatic reduction in the memory usage.

It is also notable that we could not perform the single threaded run on 1024 cores as there the memory requirements were too great, while the use of two or four threads made such a run possible. Thus, through this work, we can now perform realistic calculations on systems with more than 100,000 basis functions with as little as 0.25 GByte per core.

**Figure 18:**
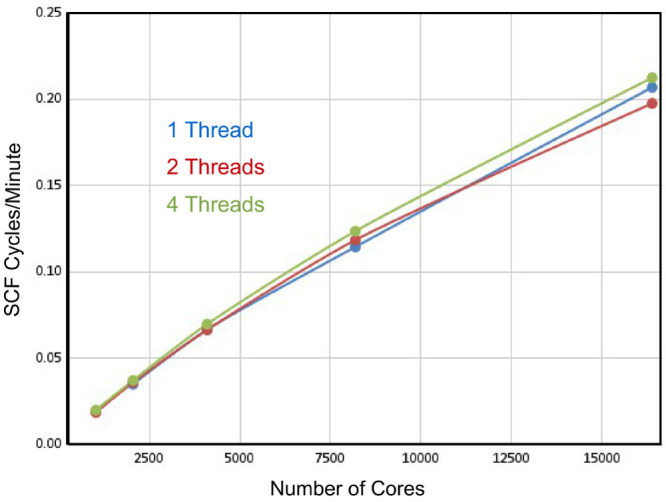
Performance of MPPCrystal for the MCM-41 X16 case as a function of the number cores and threads. All calculations were run on the Archer2 supercomputer.

Figure 18 shows the performance for the same MCM-41 X16 test case. It can be seen that use of threads little impacts this case and that the code is scaling well to 16,384 cores and beyond. Thus, in parallel, Crystal23 is capable of efficient calculations on very large systems at high core counts using minimal memory.

## 9 Quasi-Harmonic Thermoelasticity

Thermoelasticity represents the dependence of elastic mechanical properties of materials on temperature. In particular, the thermoelastic response of crystalline materials is described by the thermal dependence of all the isothermal or adiabatic elastic constants defining the fourth-rank elastic tensor, which provides the formal description of the anisotropic mechanical properties of the material in the elastic regime.^281^ An accurate description of thermoelasticity is relevant to many areas of research including (i) geophysics, where the elastic properties of minerals at temperatures of the Earth’s mantle determine the velocity of propagation of seismic waves,^282−285^ (ii) refractory materials, whose mechanical stiffnesses must not be deteriorated at high temperature,^286−289^ (iii) pharmacology, where most potential drugs are synthesized in the form of molecular crystals, whose mechanical stabilities at room temperature are crucial for an effective tableting process,^290−295^ and (iv) catalysis, where the mechanical instabilities of porous frameworks pose serious limitations to their effective use, as for metal–organic frameworks.^296−299^

The full thermoelastic characterization of a crystal requires the determination of the fourth-rank thermoelastic tensor at each desired temperature *T*, that is, the full set of isothermal elastic constants (second free-energy density derivatives with respect to pairs of strain types),
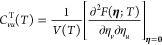
99where the superscript T is needed to distinguish isothermal from adiabatic elastic constants, *V*(*T*) is the volume of the equilibrium structure at temperature *T*, *F* is the Helmholtz free energy, η_*v*_ is one of the six independent components of the strain tensor **η**, and *v*, *u* = 1,···, 6 are Voigt indices.^300^ From inspection of eq 99, it is clear that the quantum-mechanical simulation of thermoelasticity requires the description of the lattice dynamics of the system beyond the usual harmonic approximation. This is because it involves the calculation of the free energy dependence on all lattice parameters to get (i) the anisotropic thermal expansion and (ii) the free energy second-derivatives with respect to strain.^301−304^ While the latter requires rather expensive calculations, the former can be evaluated at a reduced computational cost.

Different computational schemes can be used to compute thermoelastic constants for different classes of materials. However, they all rely on the description of thermal expansion, which is conveniently computed via the so-called quasi-harmonic approximation,^224,305^ already implemented in Crystal17 with a fully automated algorithm, which has been optimized so as to allow for the determination of the thermal expansion by computation of the harmonic phonons at just four volumes.^226−230^

### 9.1 Quasi-Static Scheme for Weakly Bound (Metal−)Organic Crystals

The thermoelastic response in soft organic or metal–organic materials (such as molecular crystals, organic semiconductors, metal–organic frameworks, etc.) is dominated by thermal expansion (positive or negative),^231,232,234,309−313^ so that, in most cases, the dependence of the free energy on the lattice parameters, *F*(**η**; *T*), can be safely substituted by that of the static energy, *E*(**η**; *T*_0_). Therefore, a simplified strategy can be introduced (so-called quasi-static approach), where thermoelastic constants are obtained from^236,237^
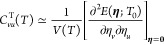
100where *T*_0_ is the absolute zero. While eq 100 still relies on the quasi-harmonic determination of *V*(*T*), now the second energy derivatives with respect to strain are evaluated from the static internal energy *E* and not from the free energy *F*, and this remarkably simplifies the corresponding calculations. Indeed, phonon frequencies need to be computed at different volumes but not at different strained configurations. Furthermore, the fully automated algorithm for the evaluation of the elastic tensor, based on the static energy gradients, can still be used.^306−308^

**Figure 19:**
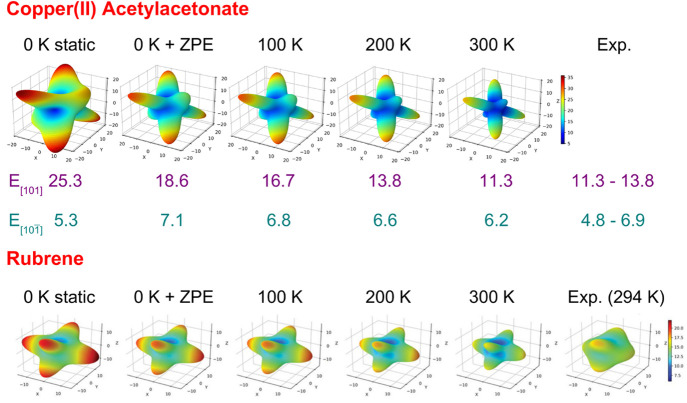
3D plots of the spatial distribution of the Young modulus of (top) copper(II) acetylacetonate crystals and (bottom) rubrene crystals, as a function of temperature. For copper(II) acetylacetonate, the value of the Young modulus along two specific directions, [101] and [101], is also reported and compared to room temperature experiments. For rubrene, the 3D plot of the experimental Young modulus at room temperature is reported for comparison. Data are in GPa.^236,237^

Let us discuss the application of this quasi-static scheme to the thermoelastic response of two prototypical soft materials: (i) the metal–organic copper(II) acetylacetonate crystal (monoclinic lattice, space group *P*2_1_/*n*): a system that has recently attracted a lot of attention because of its unusual high flexibility^314^ and its different structural mechanisms induced by temperature and strain,^315^ and (ii) the rubrene organic semiconductor (orthorhombic lattice, space group *Cmca*), which has one of the highest carrier mobilities of known crystals of this class of materials at room temperature. Figure 19 reports 3D plots of the computed spatial distribution of the Young modulus of the two systems as a function of temperature, compared to available experimental data at room temperature (the whole 3D spatial distribution of the Young modulus for rubrene and the directional Young modulus along two crystallographic directions, [101] and [101], for copper(II) acetylacetonate).^236,237^ In both cases, the thermal evolution of the computed mechanical response is very large when passing from static elastic values to room temperature ones. The sole inclusion of zero-point energy (ZPE) phonon corrections to the static picture is seen to produce very significant changes to the overall elastic response. Moreover, thermal effects do not just contribute to the “isotropic shrinking” of the elastic response but also to the change of its anisotropic distribution. In these cases, the quasi-static approach allows one to compute the thermoelastic response at room temperature at an affordable computational cost and in both qualitative and quantitative agreement with the experiment.

### 9.2 General Quasi-Harmonic Scheme for Strongly Bound Inorganic Crystals

The evaluation of thermoelastic constants through the more general eq 99 is a much more demanding computational task than that described in Section 9.1 as it requires phonons to be computed at several space group symmetry-breaking strained configurations. Furthermore, at variance with the static energy *E*, no analytical forces with respect to strain are available for the free energy *F*, which makes the evaluation of the second derivatives in eq 99 even more demanding. However, the quasi-harmonic approximation still provides a formal framework for such a task.^316−319^ We have recently suggested and implemented a quasi-harmonic scheme, where the evaluation of eq 99 is performed in two steps:^320^ (i) the determination of the equilibrium structure of the system at temperature *T* and (ii) the calculation of the second free-energy derivatives with respect to strain. The former step can be performed with the standard quasi-harmonic approach already implemented in Crystal17.^226−230^ The second derivatives of the Helmholtz free energy with respect to strain cannot be computed analytically but rather need to be evaluated numerically from the free energy of strained lattice configurations. Thus, in general, the whole set of thermoelastic constants cannot be computed by only deforming the lattice according to the six independent strain components η_*v*_. More general strains can be required that are expressed as combinations of the fundamental ones. Let us introduce the following Voigt’s vector notation for the independent strain components,
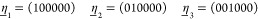

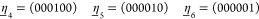
Any general strain η can thus be defined as a linear combination of the fundamental strains above,
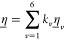
101where *k*_*v*_ are the coefficients of the linear combination defining the strain shape. The amplitude of the strain is identified by an additional parameter δ. The following scheme can be adopted to evaluate thermoelastic constants at a given temperature *T*, where a strain shape η is selected and the lattice distorted accordingly for different values of the strain amplitude δ. At each strained configuration, the harmonic vibration frequencies are computed in order to get the corresponding free energy: *F*(δ; η,*T*). The free energy is thus a function of δ and parametrically depends on the strain shape η and on the temperature *T*. The free energy computed for different values of δ can be fitted to a polynomial function and the corresponding fitting parameters *c*_0_, *c*_1_, *c*_2_, ... determined,
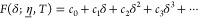
102The second free energy density derivative with respect to the strain amplitude of the expression above, at the equilibrium configuration at temperature *T*, is given by 2*c*_2_/*V*(*T*) and corresponds to a linear combination of isothermal elastic stiffness constants as follows:
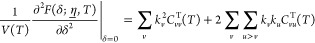
103Depending on the symmetry of the system, in order to get the full set of thermoelastic constants, several strain shapes have to be applied, each providing a linear combination of thermoelastic constants. In the present implementation, the evaluation of the derivatives in eq 103 with respect to the strain amplitude δ is fully automated and requires a single run of the program per each temperature *T* and per each strain shape η.

Let us sketch the algorithm that we have devised to compute quasi-harmonic thermoelastic constants of materials. In particular, we explicitly illustrate the sequence of calculations that are required by stressing what steps can be performed automatically in Crystal23. The algorithm that we propose is the following:^320^

1. A full structural relaxation of the system is performed (both atomic positions and lattice parameters are optimized). The static equilibrium structure, with volume *V*_0_, is obtained.
2. A space group symmetry-preserving QHA calculation is performed, which provides the thermal expansion of the system. A fully automated algorithm is implemented since Crystal17 to perform this task,^226−230^ where harmonic phonon frequencies are computed at four different volumes.
3. A value of temperature *T* is selected. Starting from the values of the lattice parameters at this temperature obtained at the end of the previous step, a volume-constrained, lattice symmetry-preserving structural relaxation is performed to get the equilibrium structure (also in terms of atomic positions) at the desired temperature.
4. A given strain shape η is chosen, which will provide a linear combination of elastic stiffness constants according to eq 103.
5. The second free energy derivatives with respect to the strain are computed. A fully automated algorithm has been implemented in the Crystal23 program for this task. The starting point is represented by the optimized structure obtained at the end of step 3 above (i.e., the equilibrium structure at temperature *T*). The structure is deformed, in terms of the strain shape η, into four strained configurations (two with positive and two with negative strain amplitude δ). At each strained configuration, atomic positions are relaxed and phonon frequencies computed. The computed quasi-harmonic free energy as a function of strain amplitude is fitted to a second-order polynomial and the corresponding second-derivative determined.

**Figure 20:**
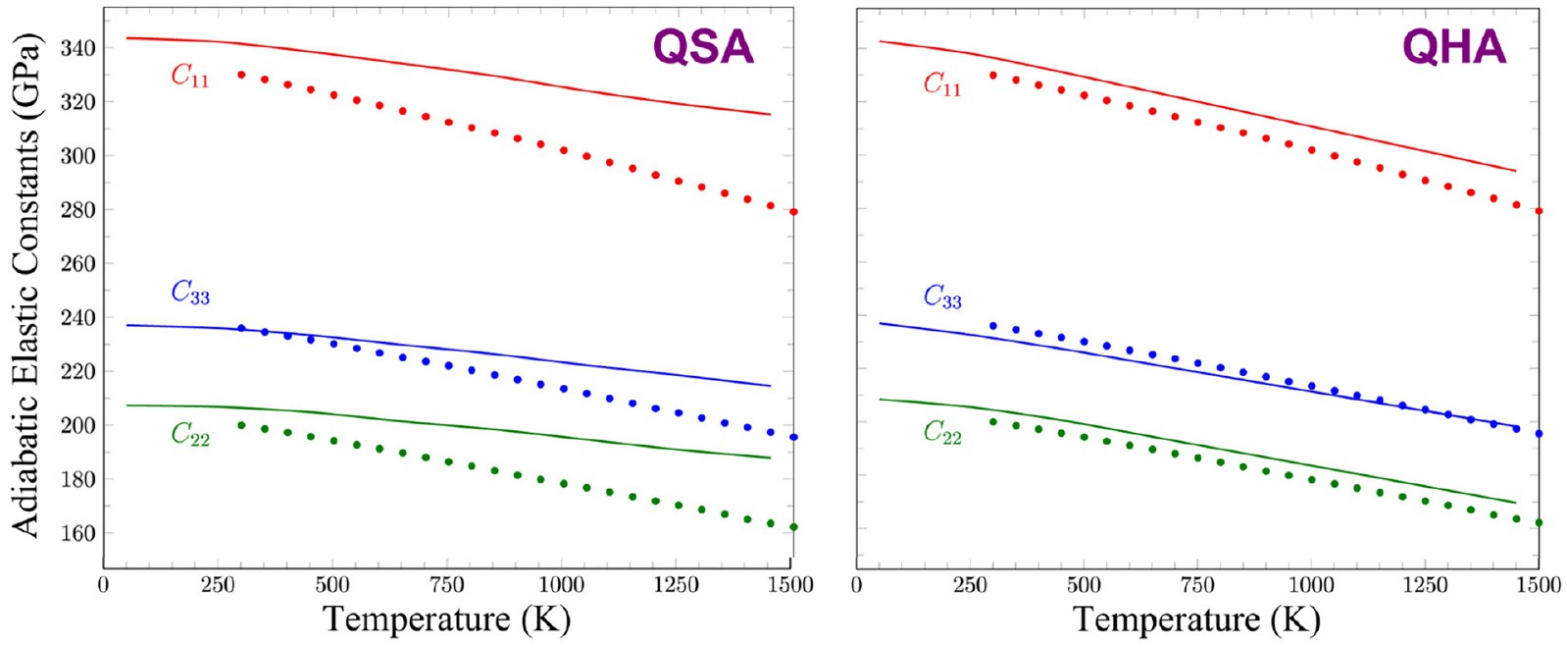
Selected adiabatic thermoelastic constants of α-Mg_2_SiO_4_ forsterite as a function of temperature as measured experimentally (circles)^321^ and as computed with our quasi-harmonic models (lines). Panels on the left and right report simulated trends with the simplified quasi-static approach of Section 9.1 and with the more explicit quasi-harmonic one of Section 9.2, respectively.

We have recently applied this quasi-harmonic algorithm to the description of the thermoelastic response of forsterite, α-Mg_2_SiO_4_: an end-member of the olivine solid solution series, that is one of the most abundant silicates in the upper mantle of the Earth.^320^ The single-crystal thermoelasticity of forsterite was accurately determined experimentally at several temperatures from 300 to 1700 K^321^ so that this system represented an ideal one to validate and discuss our methodology. Figure 20 reports selected adiabatic thermoelastic constants of forsterite as a function of temperature as measured experimentally (circles) and as computed with our quasi-harmonic models (lines). Panels on the left and right of the figure report simulated trends with the simplified quasi-static approach of Section 9.1 and with the more explicit quasi-harmonic one of Section 9.2, respectively. It is clearly seen that the simplified quasi-static approach allows one to describe a fraction of the thermal response of the system, while the more explicit scheme provides trends that are very consistent with those observed in the experiments, up to 1500 K in this case.

## 10 Multiwalled Nanotubes

In 2010, Noel and co-workers implemented an original algorithm for modeling single-walled nanotubes, which fully exploits the helical symmetry in a periodic context.^322^ The whole machinery is now extended to allow the simulation of *M*-wall nanotubes (*M* ≥ 2) by wrapping any type of layered material according to different chiralities. Again, the exploitation of helical symmetry provides a double benefit: (i) Multiwalled nanotubes can be designed by specifying just few input parameters, and (ii) very large systems can be treated with a significant saving of computational resources.^323^

Multiwalled nanotubes are cylindrical structures periodic along a single direction, conventionally taken to be *x*, consisting of concentric nanowalls of increasing diameter. Each wall can be designed and therefore completely characterized by only two integers, (*n*_1_, *n*_2_), which univocally define the corresponding rolling vector, **R**,
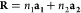
104where **a**_**1**_ and **a**_**2**_ are the lattice parameters of the 2D slab unit cell. |*R*| represents the circumference of the tube and is thus related to the nanotube diameter *D* = |*R*|/π. The angle θ between **R** and **a**_1_ is defined as the chiral angle,^324^
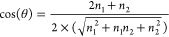
105According to their (*n*_1_, *n*_2_) indices, nanotubes fall into one of the following three categories: *armchair* (*n*_1_, *n*_1_), *zigzag* (*n*_1_, 0), or *chiral* (*n*_1_, *n*_2_ ≠*n*_1_) .^325^ Moreover, depending on **R**, two other lattice vectors are uniquely defined: (i) the nanotube lattice parameter **L**, chosen as the shortest vector perpendicular to **R** and defining the periodicity along *x*: **L** = *l*_1_**a**_**1**_ + *l*_2_**a**_**2**_ (where *l*_1_ and *l*_2_ are integers) and (ii) the helical (i.e., roto-translational) vector **H** = *h*_1_**a**_**1**_ + *h*_2_**a**_**2**_, which possesses a rotational component along the circumference vector, **R**, and a translational component along the lattice parameter, **L**, and then determines the correspondence between a translation in the 2D slab with a roto-translation on the curved surface.

The periodicity along the tube axis (i.e., the existence of the longitudinal vector **L**) is not guaranteed for all possible 2D (slab) lattices.^322^ Among the five 2D Bravais lattices, the hexagonal and square ones are the only ones that can be wrapped according to any chirality (*n*_1_, *n*_2_), whereas rectangular and rhombohedral ones can only give rise to (*n*_1_, *n*_1_) and (*n*_1_, 0) nanotubes, respectively. It is not possible to roll up an oblique lattice to get a 1D periodic nanotube.

A multiwalled structure can thus be generated by wrapping the 2D precursor in *M* tubes of gradually increasing diameter, each designed according to the rules just outlined. Once modeled, the nanomaterial can be studied by exploiting all the features (geometry optimization and manipulation, addition of defects, adsorption of molecules) and properties (electronic, vibrational, mechanical, optical, etc.) available in the Crystal code.

**Figure 21:**
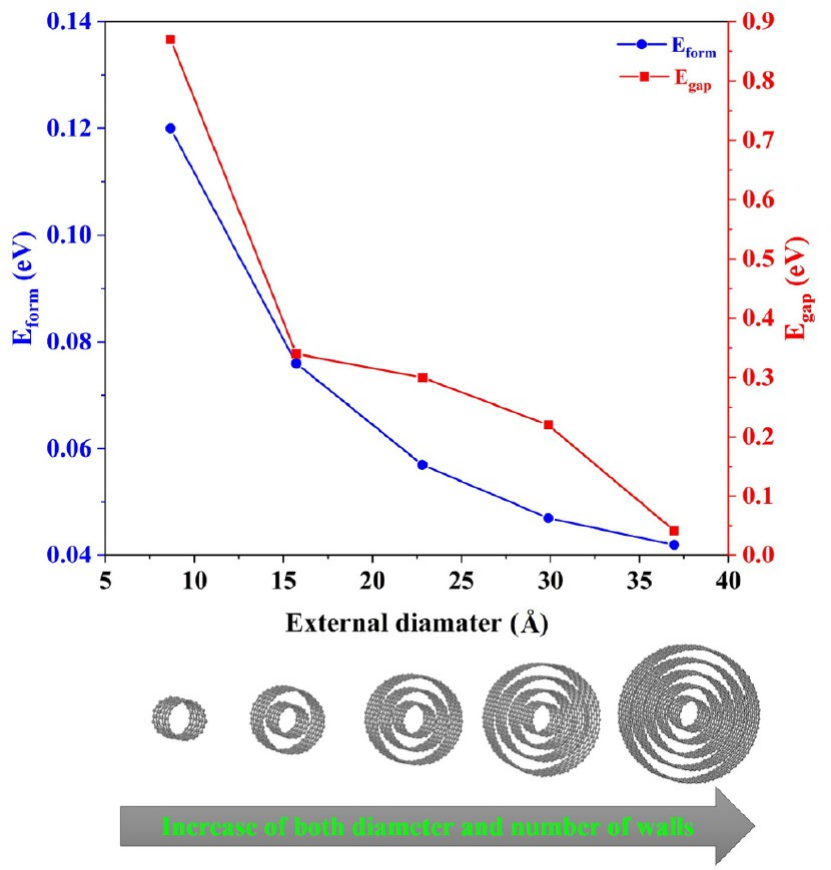
Formation energy, *E*_form_, and band gap, *E*_gap_, of carbon *zigzag* multiwalled nanotubes. From the single wall (11,0) characterized by 44 atoms in the reference cell, 88 symmetry operators, and *D* = 8.7 Å, to the M = 5 system, (11,0)@(20,0)@(29,0)@(38,0)@(47,0) with 580 atoms, 376 symmetry operators, and *D* = 37.1 Å.

For example, multiwalled stability can be analyzed in terms of the formation energy per atom, *E*_form_, defined as the energy difference of the nanotube with respect to an optimized *M*-layer 2D slab of the precursor material,
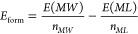
106where *E*(*MW*) and *E*(*ML*) are the energies of the optimized *M*-wall nanotube and *M*-layer slab, respectively, and *n*_*x*_ is the number of atoms in the respective reference cell (with *x* = *MW* or *ML*). In Figure 21, *E*_form_ and the electronic band gap, *E*_gap_, of a set of *zigzag* carbon multiwalled nanotubes are reported as a function of the diameter and number of walls *M*. The interwall distance is the same as the interlayer distance in graphite, this being the reference system for *M* →∞. Therefore, both the formation energy and the gap tend to zero as the number of walls increases.

**Figure 22:**
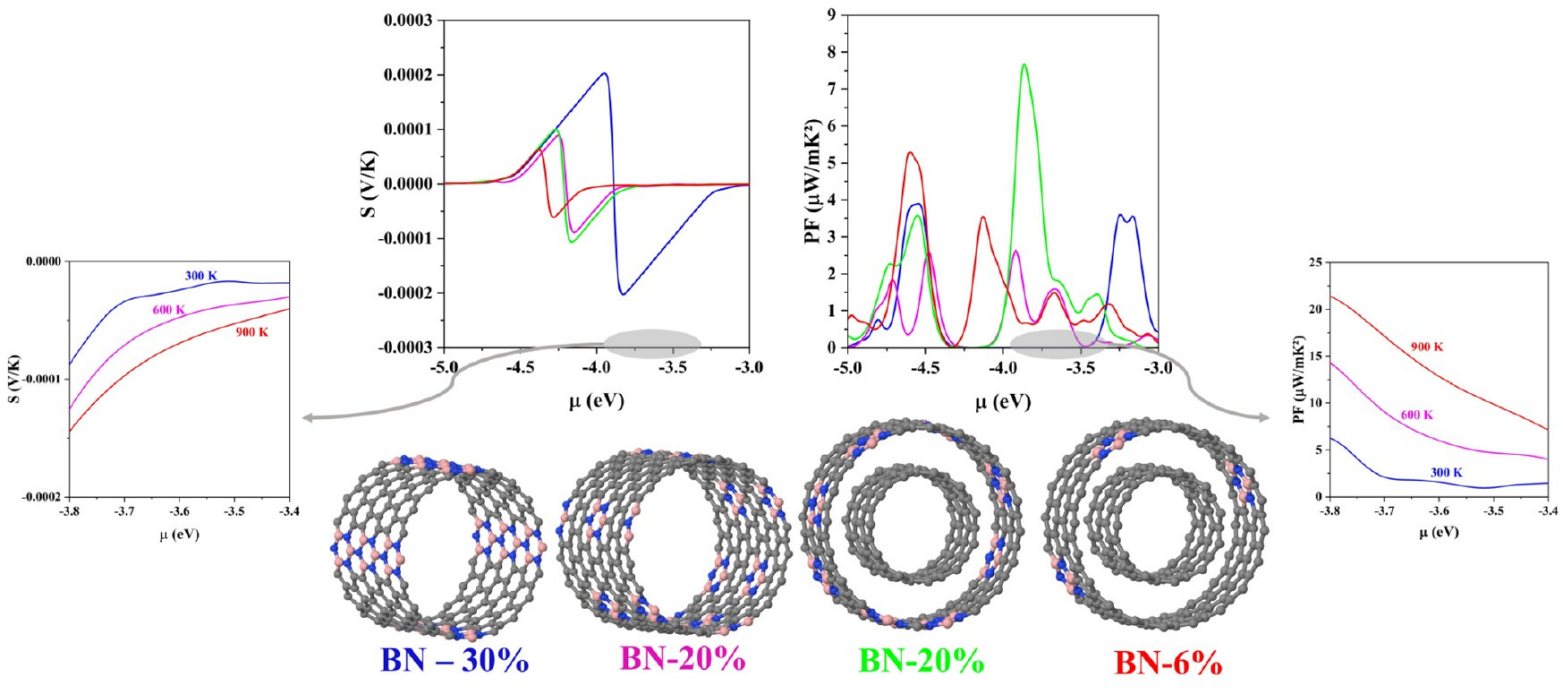
Transport properties of C@(BN)_*x*_C_1–*x*_*zigzag* double-walled nanotubes with different percentages and patterns of doping compared with the corresponding single-walled materials, (BN)_*x*_C_1–*x*_. Seebeck coefficient (left) and power factor (right). In the two insets: the dependency on temperature of *S* (left) and *PF* (right) in the range of chemical potentials corresponding to experimentally measured carrier densities.

The electronic band gap of carbon nanotubes can be engineered by doping with its isostructural boron nitride, BN, analog to obtain stable ternary structures, (BN)_1–x_C_*x*_, with specific electronic features. In Figure 22, the Seebeck coefficient (*S* = Δ*V*/Δ*T*) and the power factor (*PF* = *S*^2^σ) of single- and double-walled tubes are compared, calculated using the semiclassical Boltzmann transport equation theory as implemented in the Crystal code.^326^ The C@(BN)_1–*x*_C_*x*_ 2W structure with a 20% of BN randomly distributed in the outer wall shows a particularly good value of the power factor, and both its Seebeck and *PF* increase as the temperatures rises up. Theoretical modeling of carbon nanotubes with controlled doping can lead to the synthesis of potentially interesting materials for multiple applications, and this type of integrated design and property analysis can be performed with Crystal.

## 11 Conclusive Remarks

The main developments made to the Crystal program since the previous major version (namely, Crystal17) have been illustrated. Formal aspects of the various methodologies have been complemented with example applications to highlight their functionalities and potentials in the context of computational solid state chemistry and physics. Many of the topics covered in this review paper are still the objects of study and will constitute further developments to the code. To name a few: (i) a perturbative treatment of SOC (for energy, band structure, and density matrix, and thus density variables) to reduce the computational cost of the two-component self-consistent treatment, both in terms of CPU time and memory, (ii) generalization of the Topond module to SOC (i.e., a topological analysis of the electron density as derived from a two-component calculation), (iii) calculation of anharmonic infrared and Raman intensities from VSCF and VCI wave functions, point-symmetry exploitation in the numerical evaluation of high-order terms of the PES, and implementation of the VPT2 approach, (iv) extension of the OpenMP+MPI hybrid parallel approach to the CPHF/KS for the response to electric fields and of the massively parallel approach to the Properties module of the program and to SOC calculations, (v) implementation of a module for *ab initio* Born–Oppenheimer molecular dynamics, (vi) extension of the CPHF/KS approach to the treatment of magnetic fields, and (vii) implementation of optimally tuned range separated hybrid DFAs for solids and of Grimme’s D4 correction scheme for dispersive interactions.

## Appendix A: Computational Details of the 2c-SCF Calculations

Calculations on W-dichalcogenide monolayers were performed with the STUTSC potential (for W) and the STUTLC potentials (for Se and Te). For W, the valence basis set was of the form (6*s*6*p*4*d*2*f*)/[5*s*3*p*4*d*2*f*], being modified starting from the ecp-60-dhf-SVP set available from the Turbomole package.^327^ For Se and Te, valence basis sets of the form (5*s*5*p*2*d*)/[3*s*3*p*2*d*] were modified from the ones originally presented in ref (36). The full input decks are available in Crystal format in the Supporting Information.^82^ Reciprocal space was sampled in a 24 × 24 Monkhorst–Pack net, with Fermi smearing of 0.001 *E*_*h*_. A tolerance of 10^–8^*E*_*h*_ on the total energy was used as a convergence criterion for the SCF procedure. The five TOLINTEG parameters that control truncation of the Coulomb and exact-exchange infinite series were set to 8 8 8 8 30. The exchange-correlation functional and potential (in their collinear spin-DFT formulation) were sampled on a direct-space pruned grid over the unit-cell volume with Lebedev angular and Gauss-Legendre radial quadratures, employing 99 radial and 1454 angular points (keyword XXLGRID). The geometries of the layers were initially obtained by cleaving three-atom thick slabs along the (001) surface of the bulk *P*6_3_/*mmc* crystal structures.^328^ Then, both the atomic fractional coordinates and lattice parameters of the layers were fully optimized with analytical gradients of the total energy for systems periodic in two dimensions, and a quasi-Newton scheme, using, respectively, the PBE and PBE0 functionals at the scalar-relativistic 1c-SCF level.^329−332^ Finally, single-point 2c-SCF calculations, including SOC, were performed on the previously optimized scalar-relativistic geometries.

## Appendix B: Spin and Spin-Current Densities in Terms of the Density Matrix

Returning to eq 35a, the corresponding expressions for the spin density (or magnetization) and spin-current densities are

B1a

B1b

B1c

B1d

B1e

B1f

which shows that each of the eight density variables of SCDFT is associated with a distinct spin-block of the complex 2c-SCF direct-space density matrix.

## Supporting Information Available

explicit basis sets in Crystal format (PDF)
