# Supplementary material for: CRYSTAL23: A Program for Computational Solid State Physics and Chemistry
Source: J Chem Theory Comput. 2022 Dec 11;19(20):6891–932. doi: 10.1021/acs.jctc.2c00958 (PMC10601489; doi:10.1021/acs.jctc.2c00958)
Supplement: Supplementary file 1 — ct2c00958_si_001.pdf [file ct2c00958_si_001.pdf]

Supporting Information: CRYSTAL23: A Program for Computational Solid State Physics and Chemistry

Alessandro Erba,[*,†] Jacques K. Desmarais,[†] Silvia Casassa,[†] Bartolomeo Civalleri,[†] Lorenzo Donà,[†] Ian J. Bush,[‡] Barry Searle,[¶] Lorenzo Maschio,[†] Loredana Edith-Daga,[†] Alessandro Cossard,[†] Chiara Ribaldone,[†] Eleonora Ascrizzi,[†] Naiara L. Marana,[†] Jean-Pierre Flament,[§] and Bernard Kirtman[‖]

†*Dipartimento di Chimica, Università di Torino, via Giuria 5, 10125 Torino, Italy*

‡*Oxford e-Research Centre, University of Oxford, 7 Keble Road, Oxford OX1 3QG, U.K.*

¶*SFTC Daresbury Laboratory, Daresbury, Cheshire WA4 4AD, U.K.*

§*Université de Lille, CNRS, UMR 8523 — PhLAM — Physique des Lasers, Atomes et Molécules, 59000 Lille, France*

‖*Department of Chemistry and Biochemistry, University of California, Santa Barbara, California 93106, USA*

E-mail: alessandro.erba@unito.it

1 Non-Collinear Magnetization

Figure S1 is a magnified version of Figure 4 of the main body of the paper, to better highlight the orientation of the arrows describing the magnetization vectors at various points in space.

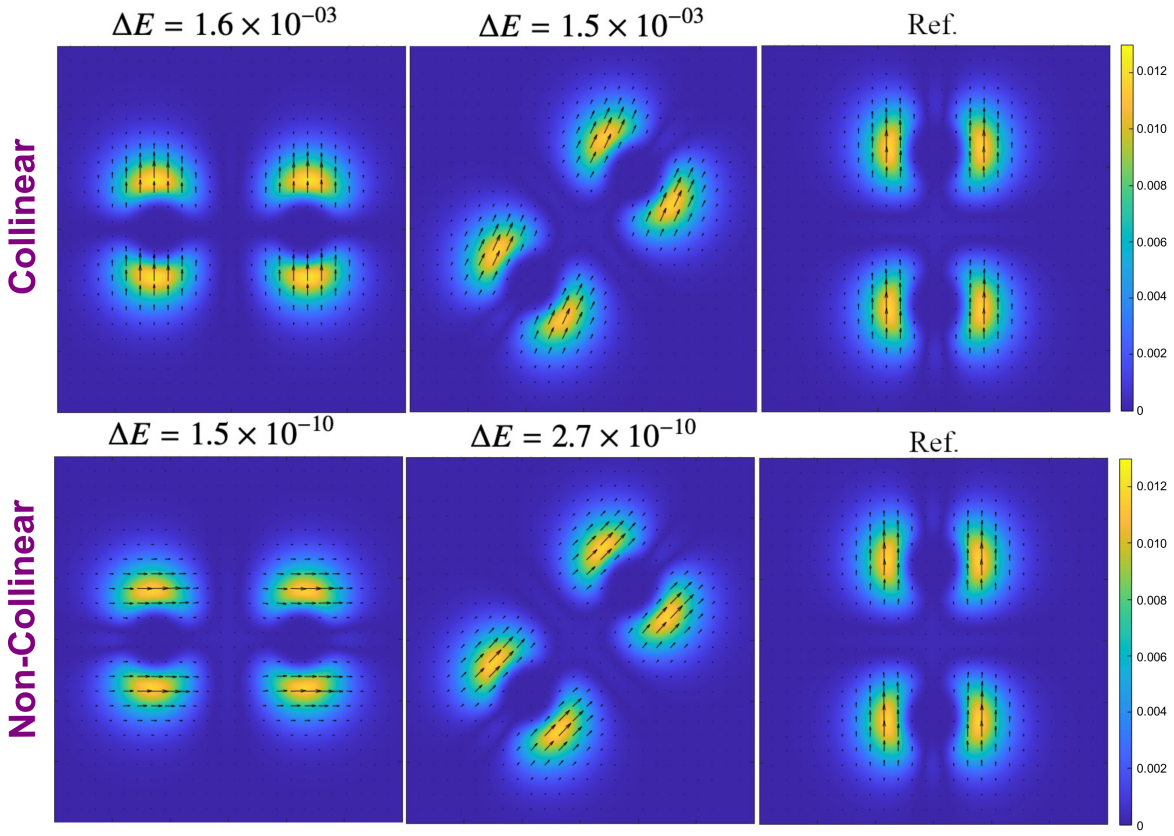

Figure S1: GGA (top panels) collinear and (bottom panels) non-collinear magnetization densities of the I_2^+ molecule, as it is rotated from the x axis to the z axis. Energy differences ΔE (in Hartree) w.r.t. the z-oriented molecule are also provided. The colour intensity represents the magnitude $m = \sqrt{m_x^2 + m_y^2 + m_z^2}$ while the arrow length and direction represent the in-plane components m_x and m_z.

2 Performance of Hybrid meta-GGA DFAs on the SS28 Dataset

Table S1: Experimental and calculated lattice constants (Å) for the SS28 dataset. For references to experimental data see *Pernot et al. J. Phys. Chem. A* **151**, 5288–5304 (2015)

	Exp.	PWB6K-D3	MPWB1K-D3	MN15-D3	revM06	MPW1B95-D3	B1B95-D3	PW6B95-D3	r^2-SCAN0-D3	MN15L-D3(0)	revM06L
AlAs	5.649	5.585	5.540	5.649	5.639	5.561	5.507	5.590	5.618	5.682	5.645
AlP	5.450	5.385	5.350	5.420	5.442	5.365	5.321	5.388	5.430	5.466	5.445
AlSb	6.126	6.047	5.992	6.096	6.109	6.011	5.943	6.049	6.095	6.180	6.174
BAs	4.764	4.719	4.693	4.768	4.768	4.711	4.679	4.734	4.754	4.810	4.772
BN	3.592	3.561	3.555	3.594	3.587	3.567	3.562	3.576	3.583	3.613	3.578
BP	4.527	4.472	4.453	4.505	4.521	4.464	4.439	4.481	4.512	4.549	4.523
CdTe	6.470	6.469	6.386	6.528	6.561	6.415	6.333	6.471	6.490	6.653	6.614
C	3.555	3.514	3.509	3.544	3.540	3.521	3.517	3.530	3.539	3.573	3.542
GaAs	5.641	5.617	5.562	5.711	5.682	5.593	5.533	5.635	5.629	5.732	5.669
GaN	4.523	4.452	4.432	4.501	4.497	4.457	4.438	4.478	4.470	4.528	4.475
GaP	5.441	5.413	5.371	5.470	5.479	5.394	5.345	5.427	5.433	5.496	5.459
GaSb	6.086	6.033	5.963	6.124	6.105	5.997	5.922	6.047	6.056	6.181	6.144
InAs	6.048	6.013	5.949	6.097	6.096	5.987	5.921	6.036	6.039	6.163	5.958
InP	5.858	5.823	5.770	5.867	5.905	5.799	5.744	5.840	5.854	5.937	5.921
InSb	6.473	6.410	6.330	6.479	6.501	6.370	6.292	6.428	6.446	6.594	6.592
Ge	5.644	5.606	5.557	5.698	5.671	5.592	5.540	5.633	5.624	5.723	5.577
LiCl	5.072	4.978	4.924	5.122	5.132	4.949	4.881	4.986	5.041	5.203	5.155
LiF	3.974	3.904	3.876	4.024	3.972	3.906	3.875	3.929	3.930	4.030	3.979
MgO	4.188	4.138	4.116	4.216	4.184	4.140	4.116	4.158	4.162	4.251	4.185
MgS	5.612	5.578	5.535	5.646	5.632	5.555	5.502	5.580	5.612	5.680	5.626
NaCl	5.565	5.499	5.436	5.646	5.608	5.469	5.387	5.507	5.541	5.665	5.608
NaF	4.570	4.515	4.476	4.630	4.558	4.510	4.468	4.536	4.524	4.604	4.555
Si	5.422	5.363	5.331	5.389	5.427	5.347	5.306	5.369	5.415	5.455	5.440
SiC	4.348	4.308	4.294	4.342	4.348	4.308	4.290	4.321	4.344	4.373	4.340
SrTiO3	3.900	3.849	3.828	3.886	3.903	3.849	3.830	3.869	3.873	3.943	3.924
ZnS	5.399	5.394	5.348	5.483	5.445	5.363	5.314	5.396	5.376	5.450	5.380
ZnSe	5.658	5.650	5.438	5.777	5.711	5.612	5.551	5.654	5.634	5.749	5.493
ZnTe	6.079	6.074	5.995	6.221	6.149	6.019	5.937	6.071	6.072	6.226	6.127

Table S2: Experimental and calculated bulk modulus (GPa) for the SS28 dataset. For references to experimental data see *Pernot et al. J. Phys. Chem. A* **151**, 5288–5304 (2015)

	Exp.	PWB6K-D3	MPWB1K-D3	MN15-D3	revM06	MPW1B95-D3	B1B95-D3	PW6B95-D3	r^2-SCAN0-D3	MN15L-D3(0)	revM06L
AlAs	75.00	91.55	96.21	87.54	86.79	91.49	96.18	87.80	88.61	87.40	86.74
AlP	87.40	105.90	110.43	101.27	98.72	105.52	110.00	101.96	100.99	103.53	101.25
AlSb	58.20	70.34	74.82	67.81	66.44	70.79	74.69	67.38	67.57	65.53	63.21
BAs	151.10	162.05	167.71	149.89	150.49	159.77	166.64	153.79	154.36	144.45	149.10
BN	410.20	441.97	444.55	414.43	420.17	428.97	429.37	421.20	422.83	409.97	429.84
BP	168.00	193.99	199.20	183.71	177.48	191.32	198.10	186.05	183.59	177.91	173.24
CdTe	45.00	46.99	50.99	44.19	44.21	48.50	51.80	45.10	46.21	37.69	40.48
C	455.90	504.90	508.28	474.34	476.31	489.26	488.80	478.65	481.75	456.11	469.78
GaAs	78.00	84.84	90.90	74.90	78.63	84.24	89.93	78.75	82.76	71.85	77.91
GaN	213.70	217.49	223.13	200.76	204.74	210.01	214.64	201.76	210.74	185.73	204.31
GaP	92.30	100.33	105.75	93.01	92.94	99.53	104.73	94.40	97.80	90.90	94.79
GaSb	57.90	67.52	73.29	59.82	62.62	67.84	73.16	62.83	65.50	55.92	58.67
InAs	58.60	70.31	75.80	62.71	64.56	69.84	74.56	64.84	67.95	57.19	60.68
InP	72.00	81.74	87.15	76.39	74.59	81.23	85.89	76.23	78.67	71.30	73.11
InSb	46.10	57.74	62.38	51.99	52.87	57.63	61.50	53.25	55.39	43.32	46.83
Ge	79.40	87.10	92.80	77.40	80.34	84.75	90.03	78.51	84.18	74.70	79.25
LiCl	38.20	41.63	44.76	34.17	34.24	42.90	47.53	41.09	38.69	34.14	35.23
LiF	76.30	90.72	93.74	74.16	81.90	89.18	92.46	87.25	87.10	79.60	84.25
MgO	169.80	197.56	201.77	166.71	183.75	192.31	197.13	187.11	185.68	160.68	178.86
MgS	78.90	67.73	70.76	62.96	65.37	68.27	71.33	66.42	66.44	65.26	67.90
NaCl	27.60	31.00	35.27	26.83	27.76	33.01	37.34	31.00	30.43	29.69	30.29
NaF	53.10	65.25	66.20	56.25	61.43	64.69	67.01	63.80	64.36	66.98	66.14
Si	101.30	112.50	116.62	107.42	101.35	110.95	115.28	106.50	105.21	107.42	99.42
SiC	229.10	255.20	259.04	240.48	240.70	249.12	254.11	243.15	240.94	235.26	243.22
SrTiO3	179.00	220.97	228.05	197.86	204.44	214.26	220.29	205.79	211.21	179.07	190.43
ZnS	75.00	83.35	88.33	75.08	80.52	84.98	89.48	81.10	85.94	76.05	85.80
ZnSe	65.90	70.80	76.14	60.90	67.39	72.70	77.57	68.61	72.02	59.60	70.30
ZnTe	52.80	55.65	61.08	47.12	52.30	58.26	63.16	54.24	55.80	44.15	51.45

Table S3: Experimental and calculated band gaps (eV) for the SS28 dataset. For references to experimental data see *Pernot et al. J. Phys. Chem. A* **151**, 5288–5304 (2015)

	Exp.	PWB6K-D3	MPWB1K-D3	MN15-D3	revM06	MPW1B95-D3	B1B95-D3	PW6B95-D3	r^2-SCAN0-D3	MN15L-D3(0)	revM06L
AlAs	2.230	3.780	3.535	3.175	3.512	2.803	2.545	2.763	3.125	1.958	2.712
AlP	2.490	3.947	3.694	3.557	3.432	2.965	2.724	2.906	3.296	1.984	2.738
AlSb	1.690	3.180	2.929	2.652	2.945	2.345	2.097	2.308	2.585	1.426	2.079
BAs	1.460	3.288	3.103	2.943	2.925	2.432	2.252	2.357	2.519	1.423	1.784
BN	6.360	7.858	7.607	7.417	7.553	6.524	6.279	6.391	6.868	4.849	5.692
BP	2.400	3.593	3.447	3.482	3.157	2.679	2.497	2.606	2.749	1.522	1.621
CdTe	1.610	3.310	3.249	1.921	2.840	2.399	2.626	2.143	2.456	0.886	1.741
C	5.490	7.483	7.262	7.041	6.790	6.242	6.017	6.092	6.155	4.058	4.506
GaAs	1.520	3.165	3.247	2.266	2.437	2.303	2.431	1.881	2.281	1.312	1.877
GaN	3.300	5.155	5.055	4.591	4.716	3.876	3.748	3.573	4.066	1.834	2.678
GaP	2.350	3.845	3.607	3.351	3.508	2.955	2.733	2.894	3.108	1.863	2.590
GaSb	0.810	2.535	2.524	1.385	1.850	1.803	1.766	1.368	1.682	0.534	1.230
InAs	0.410	2.122	2.222	0.938	1.546	1.310	1.380	0.951	1.311	0.217	1.458
InP	1.420	3.566	3.624	2.744	2.822	2.678	2.704	2.322	2.629	1.688	2.122
InSb	0.230	1.991	2.175	0.532	1.443	1.281	1.420	0.886	1.180	0.335	0.698
Ge	0.740	2.148	2.132	1.503	1.547	1.390	1.362	1.111	1.420	0.683	1.316
LiCl	9.400	10.697	10.581	9.660	9.991	9.456	9.231	9.098	9.563	7.916	8.868
LiF	14.200	15.505	15.433	13.798	14.589	13.562	13.447	13.072	13.829	10.623	11.578
MgO	7.800	9.426	9.368	8.527	8.999	7.827	7.738	7.452	8.191	5.587	6.488
MgS	5.400	6.871	6.590	6.103	6.492	5.734	5.472	5.611	6.157	4.262	5.588
NaCl	8.970	9.574	9.468	8.857	9.223	8.308	8.210	8.012	8.566	7.244	7.962
NaF	11.500	14.161	13.966	13.034	13.519	12.308	12.044	11.904	12.499	9.734	10.437
Si	1.170	2.510	2.314	2.261	2.065	1.697	1.511	1.645	1.855	0.920	0.968
SiC	2.420	3.826	3.630	3.709	3.705	2.823	2.626	2.745	3.135	1.620	2.170
SrTiO3	3.250	5.827	5.644	4.950	5.202	4.364	4.122	4.069	4.246	2.436	2.402
ZnS	3.840	5.506	5.441	4.946	4.804	4.461	4.380	4.147	4.564	3.018	3.504
ZnSe	2.820	4.415	4.795	3.491	3.798	3.354	3.299	3.018	3.462	1.914	2.997
ZnTe	2.390	4.277	4.278	3.034	3.676	3.351	3.333	2.982	3.330	1.594	2.393

3 Input Files

We report below a few example input files for some of the calculations performed for this study.

Spin-Orbit Coupling SCF Calculation on 2D WSe_2

```
WSe2 SOC
CRYSTAL
0 0 0
194
3.1532 12.323
2
274 0.333333333334 0.666666666667 0.25000
234 0.333333333334 0.666666666667 0.62250
SLABCUT
0 0 1
1 3
```

```
SYMMREMO
END
274 14
STUTSC
0 0 2 2. 1.
 15.000000000 -0.53984569304
 12.000000000 1.0228484726
0 0 1 2. 1.
 5.2610967725 1.0000000000
0 0 1 0. 1.
 0.92785370307 1.0000000000
0 0 1 0. 1.
 0.40334458241 1.0000000000
0 0 1 0. 1.
 0.15 1.0000000000
0 2 4 6. 1.
 7.2496570000 0.46749049338
 6.0848760000 -0.67718942302
 1.2523777812 0.53559619861
 0.58569208922 0.49083198365
0 2 1 0. 1.
 0.45 1.0000000000
0 2 1 0. 1.
 0.15 1.0000000000
0 3 1 4. 1.
 4.0131231332 1.0000000000
0 3 1 0. 1.
 1.6237452450 1.0000000000
0 3 1 0. 1.
```

```
 0.69187452392 1.0000000000
0 3 1 0. 1.
 0.27865835325 1.0000000000
0 4 1 0. 1.
 0.9 1.0
0 4 1 0. 1.
 0.3 1.0
234 8
STUTLC
0 0 3 2. 1
3.300301 0.385816
2.434170 -0.668512
0.713896 -0.384598
0 0 1 0. 1
0.528812 1.0
0 0 1 0. 1
0.211428 1.0
0 2 3 4. 1
5.153564 0.011117
2.057511 -0.102428
0.604512 0.226377
0 2 1 0. 1
0.291191 1.0
0 2 1 0. 1
0.133145 1.0
0 3 1 0. 1.
 0.9 1.0
0 3 1 0. 1.
 0.3 1.0
```

```
99 0
END
TWOCOMPON
SOC
ENDTWO
DFT
XXLGRID
PBE0
ENDDFT
SHRINK
24 24
FMIXING
60
SMEAR
0.001
TOLINTEG
8 8 8 8 30
TOLDEE
8
MAXCYCLE
1000
END
```

Spin-Orbit Coupling SCF Calculation on 2D WTe_2

```
WTe2 SOC
comment
CRYSTAL
0 0 0
194
```

```
3.1532 12.323
2
274 0.333333333334 0.666666666667 0.25000
252 0.333333333334 0.666666666667 0.62250
SLABCUT
0 0 1
1 3
END
274 14
STUTSC
0 0 2 2. 1.
 15.000000000 -0.53984569304
 12.000000000 1.0228484726
0 0 1 2. 1.
 5.2610967725 1.0000000000
0 0 1 0. 1.
 0.92785370307 1.0000000000
0 0 1 0. 1.
 0.40334458241 1.0000000000
0 0 1 0. 1.
 0.15 1.0000000000
0 2 4 6. 1.
 7.2496570000 0.46749049338
 6.0848760000 -0.67718942302
 1.2523777812 0.53559619861
 0.58569208922 0.49083198365
0 2 1 0. 1.
 0.45 1.0000000000
0 2 1 0. 1.
```

```
 0.15 1.0000000000
0 3 1 4. 1.
 4.0131231332 1.0000000000
0 3 1 0. 1.
 1.6237452450 1.0000000000
0 3 1 0. 1.
 0.69187452392 1.0000000000
0 3 1 0. 1.
 0.27865835325 1.0000000000
0 4 1 0. 1.
 0.9 1.0
0 4 1 0. 1.
 0.3 1.0
252 8
STUTLC
0 0 3 2. 1
 4.620870 -0.076259
 3.407086 0.222163
 1.353795 -0.541514
0 0 1 0. 1
 0.278218 1.0
0 0 1 0. 1
 0.128403 1.0
0 2 3 4. 1
 4.772823 -0.038412
 3.508559 0.112992
 1.653984 -0.229605
0 2 1 0. 1
 0.326880 1.0
```

```
0 2 1 0. 1
 0.139746 1.0
0 3 1 0. 1.
 0.9 1.0
0 3 1 0. 1.
 0.3 1.0
99 0
END
TWOCOMPON
SOC
ENDTWO
DFT
XXLGRID
PBE0
ENDDFT
SHRINK
24 24
FMIXING
60
SMEAR
0.001
TOLINTEG
8 8 8 8 30
TOLDEE
8
MAXCYCLE
1000
END
```

Spin-Orbit Coupling SCF Calculation on 3D TaAs Weyl Semi-metal

```
TaAs SOC
CRYSTAL
0 0 0
109
3.437 11.656
2
273 0. 0. 0.
233 0. 0. 0.416
END
273 13
STUTSC
0 0 5 2. 1.
  24.473650944      0.48239461915E-01
  18.721372549     -0.11130803862
  11.500000000      -4.3871385439
  10.350000000       14.773276225
  9.7732783383      -10.295986879
0 0 1 0. 1.
  3.8125414615       1.0000000000
0 0 1 0. 1.
  1.0507430630       1.0000000000
0 0 1 0. 1.
 0.49732275755       1.0000000000
0 0 1 0. 1.
 0.15602650970       1.0000000000
0 2 4 6. 1.
  23.290413736      0.13565200450E-01
  17.000000000     -0.74349450243E-01
  12.008186536      0.14135027656
```

```
  5.0278760583     -0.29185231563
0 2 1 0. 1.
  1.1937124184      0.52092880326
0 2 1 0. 1.
 0.57889707053       1.0000000000
0 2 1 0. 1.
 0.27225198801       1.0000000000
0 2 1 0. 1.
 0.10000000000       1.0000000000
0 3 3 2. 1.
  6.4242952246      0.94769104117E-01
  5.1122245125     -0.18492990619
  1.2009867996      0.44048238003
0 3 1 0. 1.
 0.51923142085       1.0000000000
0 3 1 0. 1.
 0.21323328623       1.0000000000
233 5
STUTLC
0 0 3 2. 1
3.069423 0.332502
2.268204 -0.565786
0.858631 -0.168471
0 0 1 0. 1
0.415970 1.0
0 0 1 0. 1
0.180588 1.0
0 2 3 6. 1
1.275524 -0.321208
```

```
0.942483 0.300478
0.289765 0.466271
0 2 1 0. 1
0.125374 1.0
99 0
END
TWOCOMPON
SOC
PRTENESOC
ENDTWO
SDFT
NONCOLC
SVWN
HYBRID
25
ENDDFT
SHRINK
30 30
MAXCYCLE
200
NOSHIFT
FMIXING
70
TOLINTEG
8 8 8 8 20
SMEAR
0.001
TOLDEE
7
```

```
END
```

Equation-of-State Calculation on 3D SiC with the PBEsol0-3c Composite Method

```
SiC EOS
CRYSTAL
0 0 1
216
4.361
2
14  0.0000  0.0000  0.0000
 6  0.2500  0.2500  0.2500
EOS
PREOPTGEOM
END
BASISSET
SOLDEF2MSVP
DFT
PBESOL03C
END
SHRINK
8 8
TOLINTEG
7 7 7 7 25
FMIXING
50
END
```

Equation-of-State Calculation on 3D AlAs with the MPW1B95-D3 Hybrid meta-GGA Method

```
AlAs EOS
CRYSTAL
0 0 1
216
5.643
2
 13  0.00 0.00 0.00
233  0.25 0.25 0.25
EOS
PREOPTGEOM
END
END
13 13
0 0 6 2. 1.
       54866.4890         0.839000000E-03
       8211.76650         0.652700000E-02
       1866.17610         0.336660000E-01
       531.129340         0.132902000
       175.117970         0.401266000
       64.0055000         0.531338000
0 0 3 2. 1.
       64.0055000         0.202305000
       25.2925070         0.624790000
       10.5349100         0.227439000
0 0 1 2. 1.
       3.20671100          1.00000000
0 0 1 0. 1.
       1.15255500          1.00000000
0 0 1 0. 1.
```

```
       0.70000000          1.00000000
0 0 1 0. 1.
       0.35000000          1.00000000
0 0 1 0. 1.
      0.176678000          1.00000000
0 2 4 6. 1.
       259.283620         0.944800000E-02
       61.0768700         0.709740000E-01
       19.3032370         0.295636000
       7.01088200         0.728219000
0 2 2 1. 1.
       2.67386500         0.644467000
       1.03659600         0.417413000
0 2 1 0. 1.
      0.700000000          1.00000000
0 2 1 0. 1.
      0.316819000          1.00000000
0 2 1 0. 1.
      0.150          1.00000000
0 3 1 0. 1.
      0.6000000          1.00000000
233 9
INPUT
23. 0 2 4 6 2 0
28.725122 370.114025 0
6.767681 9.349296 0
45.331064 99.142103 0
44.767415 198.307880 0
19.539090 28.383073 0
```

```
18.973471 56.871464 0
51.057152 -18.485145 0
50.151340 -28.113530 0
16.108936 -1.223895 0
14.672223 -1.345765 0
3.851927 0.101757 0
3.813502 0.170338 0
11.940584 -0.775230 0
17.761160 -2.157259 0
0 0 6 2. 1.
       2542.81000          0.113700000E-02
       381.169000          0.605500000E-02
       40.2342000          0.841250000E-01
       16.1217000         -0.405285000
       3.20189000          0.712926000
       1.42096000          0.473376000
0 0 6 2. 1.
       2542.81000         -0.390000000E-03
       381.169000         -0.219000000E-02
       40.2342000         -0.268530000E-01
       16.1217000          0.136878000
       3.20189000         -0.320457000
       1.42096000         -0.337391000
0 0 1 0. 1.
      0.321443000           1.00000000
0 0 1 0. 1.
      0.120000000           1.00000000
0 2 6 6. 1.
       99.5349000          0.385700000E-02
```

```
        24.1195000          -0.851010000E-01
        5.84196000           0.404762000
        2.56010000           0.531478000
        1.09308000           0.184012000
       0.318424000           0.576400000E-02
0 2 6 3. 1.
        99.5349000          -0.772000000E-03
        24.1195000           0.199410000E-01
        5.84196000          -0.107210000
        2.56010000          -0.172259000
        1.09308000           0.876100000E-02
       0.318424000           0.569744000
0 2 1 0. 1.
       0.120000000            1.00000000
0 3 6 10. 1.
        113.509000           0.119800000E-01
        36.8872000           0.795440000E-01
        13.6893000           0.236755000
        5.38964000           0.401534000
        2.08046000           0.406686000
       0.737568000           0.173162000
0 3 1 0. 1.
       0.307800000            1.00000000
99 0
END
DFT
MPW1B95
END
SHRINK
```

```
8 8
TOLINTEG
7 7 7 7 25
FMIXING
50
DFTD3
VERSION
4
METHOD
MPW1B95
ABC
END
END
```

Equation-of-State Calculation on 3D Silicon with the REVM06 Hybrid meta-GGA Method

```
Si EOS REVM06
CRYSTAL
0 0 1
227
5.420476
1
14  0.0000  0.0000  0.0000
EOS
PREOPTGEOM
END
END
14 12
0 0 6 2. 1.
```

```
     69379.2300          0.757000000E-03
     10354.9400          0.593200000E-02
     2333.87960          0.310880000E-01
     657.142950          0.124967000
     214.301130          0.386897000
     77.6291680          0.554888000
0 0 3 2. 1.
     77.6291680          0.177881000
     30.6308070          0.627765000
     12.8012950          0.247623000
0 0 1 2. 1.
     3.92686600           1.00000000
0 0 1 0. 1.
     1.45234300           1.00000000
0 0 1 0. 1.
    0.256234000           1.00000000
0 0 1 0. 1.
    0.120000000           1.00000000
0 2 4 6. 1.
     335.483190          0.886600000E-02
     78.9003660          0.682990000E-01
     24.9881500          0.290958000
     9.21971100          0.732117000
0 2 2 2. 1.
     3.62114000          0.619879000
     1.45131000          0.439148000
0 2 1 0. 1.
    0.504977000           1.00000000
0 2 1 0. 1.
```

```
      0.250000000          1.00000000
0 2 1 0. 1.
      0.120000000          1.00000000
0 3 1 0. 1.
      0.450000000          1.00000000
99 0
END
DFT
REVM06
END
SHRINK
8 8
TOLINTEG
7 7 7 7 25
FMIXING
50
END
```

Polarizability Tensor Calculation on the 3D molecular crystal of m-nitroaniline with the Range-separated Hybrid HSE06 Method

```
mNA CPHF HSE06
CRYSTAL
1 0 0
P b c 21
6.499 19.369 5.084
16
6      2.248514453420E-01 -3.774136729596E-01 -2.739677961668E-01
6     -4.589413860115E-01 -3.461003080877E-01 -4.859655524735E-01
6      3.886395189496E-01 -3.320624201184E-01 -2.994868292382E-01
```

```
1      3.969932439533E-01 -2.868836567859E-01 -1.765591708395E-01
6     -4.791174315484E-01 -4.055040381485E-01  3.581243494293E-01
1     -3.621689659282E-01 -4.163876571834E-01  2.131850563172E-01
7     -2.964921175350E-01 -3.009188086251E-01  4.766613148214E-01
1     -1.694506887317E-01 -3.201675132057E-01  3.887552831168E-01
1     -2.672703031038E-01 -2.685339962400E-01 -3.716154685396E-01
7      6.896647410906E-02 -3.630440284319E-01 -7.409985241059E-02
8     -7.283658361658E-02 -4.043735474013E-01 -4.846502422240E-02
6      2.031694662423E-01 -4.364178663353E-01 -4.262903051719E-01
1      7.495645192172E-02 -4.708001105160E-01 -3.989948770684E-01
8      8.572268901539E-02 -3.108792399116E-01  6.088388406456E-02
6      3.548612556717E-01 -4.496683640080E-01  3.885177238157E-01
1      3.443305933233E-01 -4.954116909297E-01  2.677172645658E-01
CPHF
TOLALPHA
3
THIRD
MAXCYCLE
250
END
ENDG
6 7
0 0 6 2.0 1.00
  0.4563240000D+04  0.1966650000D-02
  0.6820240000D+03  0.1523060000D-01
  0.1549730000D+03  0.7612690000D-01
  0.4445530000D+02  0.2608010000D+00
  0.1302900000D+02  0.6164620000D+00
  0.1827730000D+01  0.2210060000D+00
```

```
0 1 3 4.0 1.00
  0.2096420000D+02  0.1146600000D+00  0.4024870000D-01
  0.4803310000D+01  0.9199990000D+00  0.2375940000D+00
  0.1459330000D+01 -0.3030680000D-02  0.8158540000D+00
0 1 1 0.0 1.00
  0.4834560000D+00  0.1000000000D+01  0.1000000000D+01
0 1 1 0.0 1.00
  0.1455850000D+00  0.1000000000D+01  0.1000000000D+01
0 3 1 0.0 1.00
       1.25200000          1.00000000
0 3 1 0.0 1.00
      0.313000000          1.00000000
0 4 1 0.0 1.00
      0.800000000          1.00000000
8 7
0 0 6 2.0 1.00
  0.8588500000D+04  0.1895150000D-02
  0.1297230000D+04  0.1438590000D-01
  0.2992960000D+03  0.7073200000D-01
  0.8737710000D+02  0.2400010000D+00
  0.2567890000D+02  0.5947970000D+00
  0.3740040000D+01  0.2808020000D+00
0 1 3 6.0 1.00
  0.4211750000D+02  0.1138890000D+00  0.3651140000D-01
  0.9628370000D+01  0.9208110000D+00  0.2371530000D+00
  0.2853320000D+01 -0.3274470000D-02  0.8197020000D+00
0 1 1 0.0 1.00
  0.9056610000D+00  0.1000000000D+01  0.1000000000D+01
0 1 1 0.0 1.00
```

```
  0.2556110000D+00  0.1000000000D+01  0.1000000000D+01
0 3 1 0.0 1.00
       2.58400000          1.00000000
0 3 1 0.0 1.00
      0.646000000          1.00000000
0 4 1 0.0 1.00
       1.40000000          1.000000
7 7
0 0 6 2.0 1.00
  0.6293480000D+04  0.1969790000D-02
  0.9490440000D+03  0.1496130000D-01
  0.2187760000D+03  0.7350060000D-01
  0.6369160000D+02  0.2489370000D+00
  0.1882820000D+02  0.6024600000D+00
  0.2720230000D+01  0.2562020000D+00
0 1 3 5.0 1.00
  0.3063310000D+02  0.1119060000D+00  0.3831190000D-01
  0.7026140000D+01  0.9216660000D+00  0.2374030000D+00
  0.2112050000D+01 -0.2569190000D-02  0.8175920000D+00
0 1 1 0.0 1.00
  0.6840090000D+00  0.1000000000D+01  0.1000000000D+01
0 1 1 0.0 1.00
  0.2008780000D+00  0.1000000000D+01  0.1000000000D+01
0 3 1 0.0 1.00
       1.82600000          1.00000000
0 3 1 0.0 1.00
      0.456500000          1.00000000
0 4 1 0.0 1.00
       1.00000000          1.00000000
```

```
1 6
0 0 3 1.0 1.00
  0.3386500000D+02  0.2549380000D-01
  0.5094790000D+01  0.1903730000D+00
  0.1158790000D+01  0.8521610000D+00
0 0 1 0.0 1.00
  0.3258400000D+00  0.1000000000D+01
0 0 1 0.0 1.00
  0.1027410000D+00  0.1000000000D+01
0 2 1 0.0 1.00
       1.50000000          1.00000000
0 2 1 0.0 1.00
      0.375000000          1.00000000
0 3 1 0.0 1.00
       1.00000000          1.00000000
99 0
ENDBS
DFT
HSE06
XLGRID
END
TOLINTEG
7 7 7 7 25
SCFDIR
SHRINK
4 4
FMIXING
50
TOLDEE
```

```
8
LEVSHIFT
6 1
MAXCYCLE
100
ENDSCF
```

VSCF and VCI Anharmonic Calculation on Molecular Water

```
Water
MOLECULE
13
2
8   1.548709341513E-01  0.000000000000E+00  0.000000000000E+00
1   6.811665329244E-01  0.000000000000E+00  7.902246287948E-01
FREQCALC
NUMDERIV
2
ANHAPES
3
7 8 9
2 0.5
VSCF
VSCFTOL
4
VSCFMIX
70
VCI
5 3
0
```

```
END
END
8 4
0 0  6  2. 1.00
   5484.6717000              0.0018311
    825.2349500              0.0139501
    188.0469600              0.0684451
     52.9645000              0.2327143
     16.8975700              0.4701930
      5.7996353              0.3585209
0 1 3 6. 1.
     15.5396160             -0.1107775              0.0708743
      3.5999336             -0.1480263              0.3397528
      1.0137618              1.1307670              0.7271586
0 1 1 0. 1.
      0.2700058              1.0000000              1.0000000
0 1 1 0. 1.
      0.0845000              1.0000000              1.0000000
1 2
0 0 3 1. 1.
     18.7311370              0.03349460
      2.8253937              0.23472695
      0.6401217              0.81375733
0 0 1 0. 1.
      0.1612778              1.0000000
99 0
END
TOLINTEG
10 10 10 15 30
```

```
FMIXING
30
TOLDEE
12
END
```

VSCF and VCI Anharmonic Calculation on Molecular Methane

```
CH4 Anharmonicity
MOLECULE
44
2
6   0.      0.      0.
1   0.629   0.629   0.629
FREQCALC
NUMDERIV
2
ANHAPES
9
7 8 9 10 11 12 13 14 15
3 0.9
VSCF
VCI
5 3
0
END
END
6 2
1 0 3 2. 0.
1 1 3 4. 0.
```

```
1 1
1 0 3 1. 0.
99 0
END
TOLINTEG
10 10 10 15 30
FMIXING
30
TOLDEE
12
END
```

Thermoelastic Calculation on 3D MgO for C_{44} at 10 K

```
MgO Thermoelasticity
CRYSTAL
0 0 0
225
4.26656466
2
 12 0.    0.    0.
  8 0.5   0.5   0.5
ELASTCON
PRINT
HNUMDERIV
2
THERMOELAST
10.
0 0 0 1 0 0
END
```

```
END
12 4
0 0 8 2.0 1.0
 68370.0 0.0002226
 9661.0 0.001901
 2041.0 0.011042
 529.6 0.05005
 159.17 0.1690
 54.71 0.36695
 21.236 0.4008
 8.791 0.1487
0 1 5 8.0 1.0
 143.7 -0.00671 0.00807
 31.27 -0.07927 0.06401
 9.661 -0.08088 0.2092
 3.726 0.2947 0.3460
 1.598 0.5714 0.3731
0 1 1 2.0 1.0
 0.688 1.0 1.0
0 1 1 0.0 1.0
 0.28 1.0 1.0
8 5
0 0 8 2. 1.
8020.  0.00108
1338.  0.00804
255.4  0.05324
69.22  0.1681
23.90  0.3581
9.264  0.3855
```

```
3.851  0.1468
1.212  0.0728
0 1 4 6. 1.
49.43 -0.011 0.0097
10.47 -0.091 0.069
3.235 -0.039 0.207
1.22 0.379 0.347
0 1 1 0. 1.
0.459019090935  1. 1.
0 1 1 0. 1.
0.166171528548 1. 1.
0 3 1 0. 1.
0.883705090207  1.
99 0
END
DFT
EXCHANGE
PBE
CORRELAT
PBE
END
SHRINK
6 6
LEVSHIFT
8 1
FMIXING
40
END
```

SCF Calculation on a Two-Walled 1D Carbon Nanotube

```
CARBON-NANOTUBE-ARMCHAIR
CRYSTAL
0 0 0
186
2.47   6.70
2
6  0.00000 0.00000 0.00000
6  0.33333 0.66667 0.00000
SLAB
0 0 1
1 1
NANOMULTI
2
WALL
1
ROLLINGV
8 0
WALL
2
ROLLINGV
12 0
ROTWALL
0.71
ENDWALL
END
END
6 4
0 0 6 2.0 1.0
 3048.0 0.001826
```

```
 456.4 0.01406
 103.7 0.06876
 29.23 0.2304
 9.349 0.4685
 3.189 0.3628
0 1 2 4.0 1.0
 3.665 -0.3959 0.2365
 0.7705 1.216 0.8606
0 1 1 0.0 1.0
 0.1959 1.0 1.0
0 3 1 0.0 1.0
 0.8 1.0
99 0
ENDBS
DFT
PBE
XXLGRID
END
SCFDIR
TOLINTEG
8 8 8 8 16
SHRINK
8 8
MAXCYCLE
1000
END
```

SCF Calculation on a Three-Walled 1D BN Nanotube

```
NANOTUBE BN ARMCHAIR
```

```
CRYSTAL
0 0 0
187
2.49824 6.6357
4
5 0.0 0.0 0.5
5 0.3333 0.6667 0.0
7 0.0 0.0 0.0
7 0.3333 0.6667 0.5
SLAB
0 0 1
1 1
NANOMULTI
3
WALL
1
ROLLINGV
7 7
OPTWALL
WALL
2
ROLLINGV
12 12
OPTWALL
WALL
3
ROLLINGV
17 17
OPTWALL
```

```
OPTMULTI
ENDWALL
END
END
5 4
0 0 6 2.0 1.0
 2.082E+03 1.850E-03
 3.123E+02 1.413E-02
 7.089E+01 6.927E-02
 1.985E+01 2.324E-01
 6.292E+00 4.702E-01
 2.129E+00 3.603E-01
0 1 2 3.0 1.0
 2.282E+00 -3.687E-01 2.312E-01
 4.652E-01 1.199E+00 8.668E-01
0 1 1 0.0 1.0
 1.243E-01 1.000E+00 1.000E+00
0 3 1 0.0 1.0
 0.8 1.0
7 4
0 0 6 2.0 1.0
 4150.0 0.001845
 620.1 0.01416
 141.7 0.06863
 40.34 0.2286
 13.03 0.4662
 4.47 0.3657
0 1 2 5.0 1.0
 5.425 -0.4133 0.238
```

```
 1.149 1.224 0.859
0 1 1 0.0 1.0
 0.2832 1.0 1.0
0 3 1 0.0 1.0
 0.8 1.0 1.0
99 0
END
DFT
PBE
END
TOLINTEG
8 8 8 8 16
SHRINK
6 6
MAXCYCLE
500
FMIXING
80
END
```
